# A rechargeable molecular solar thermal system below 0 °C†

**DOI:** 10.1039/d2sc01873j

**Published:** 2022-05-16

**Authors:** Zhichun Shangguan, Wenjin Sun, Zhao-Yang Zhang, Dong Fang, Zhihang Wang, Si Wu, Chao Deng, Xianhui Huang, Yixin He, Ruzhu Wang, Tingxian Li, Kasper Moth-Poulsen, Tao Li

**Affiliations:** School of Chemistry and Chemical Engineering, Frontiers Science Center for Transformative Molecules, Shanghai Key Laboratory of Electrical Insulation and Thermal Aging, Key Laboratory of Thin Film and Microfabrication, Ministry of Education, Shanghai Jiao Tong University Shanghai 200240 China litao1983@sjtu.edu.cn; Department of Chemistry and Chemical Engineering, Chalmers University of Technology Gothenburg 41296 Sweden kasper.moth-poulsen@chalmers.se; Research Center of Solar Power & Refrigeration, School of Mechanical Engineering, Shanghai Jiao Tong University Shanghai 200240 China; College of Chemistry & Materials Engineering, Wenzhou University Wenzhou 325027 Zhejiang China; The Institute of Materials Science of Barcelona, ICMAB-CSIC 08193 Bellaterra Barcelona Spain; Catalan Institution for Research & Advanced Studies, ICREA Pg. Lluís Companys 23 Barcelona Spain

## Abstract

An optimal temperature is crucial for a broad range of applications, from chemical transformations, electronics, and human comfort, to energy production and our whole planet. Photochemical molecular thermal energy storage systems coupled with phase change behavior (MOST-PCMs) offer unique opportunities to capture energy and regulate temperature. Here, we demonstrate how a series of visible-light-responsive azopyrazoles couple MOST and PCMs to provide energy capture and release below 0 °C. The system is charged by blue light at −1 °C, and discharges energy in the form of heat under green light irradiation. High energy density (0.25 MJ kg^−1^) is realized through co-harvesting visible-light energy and thermal energy from the environment through phase transitions. Coatings on glass with photo-controlled transparency are prepared as a demonstration of thermal regulation. The temperature difference between the coatings and the ice cold surroundings is up to 22.7 °C during the discharging process. This study illustrates molecular design principles that pave the way for MOST-PCMs that can store natural sunlight energy and ambient heat over a wide temperature range.

## Abstract 2

We demonstrate rationally designed arylazopyrazoles as MOST-PCM that can be circularly charged and discharged below 0 °C with visible light.

## Graphical abstract

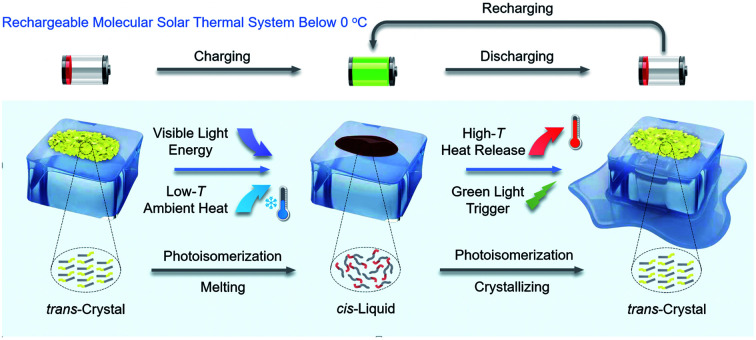

## Introduction

Thermal management is crucial in our modern society, regardless of whether we are considering chemical transformations, electronics, human comfort, energy production or our whole planet. Thermal management materials based on specific heat capacity or phase change are seeing increased use in applications such as electronics, and domestic and industrial heat management.^1–5^ Phase change materials (PCMs) are a broad class of materials whose latent heat during a phase transition from solid-to-liquid can be used for energy storage applications. Latent heat storage offers a significant advantage if the application involves temperature cycles close to the melting point since in those cases, the corresponding storage density of sensible thermal storage is small. In building applications, phase change materials made from paraffins, salt hydrates, fatty acids or ice can be used as central heat sinks^6^ and also in floors, windows or walls.^7,8^ Common to all “traditional” thermal energy storage materials is that they operate *via* heat transfer, both in energy input and energy output.^9^ This leads to design challenges and scaling factors that restrict practical performance and implementation.

Molecular solar thermal (MOST) systems have been recognized as a promising avenue to harvest and store thermal energy.^10–15^ In the charging process, a stable isomer of a photochromic molecule absorbs photon energy and is converted into a high-energy metastable isomer, thereby storing solar energy in chemical bonds. The MOST system is discharged when the metastable isomer switches back to the stable isomer by external stimuli, with the release of stored energy in the form of heat. While the MOST system shares some properties with PCMs, the process of energy storage and release in the MOST system is controlled by photons and molecular thermodynamics,^16,17^ whereas in PCMs it is controlled by heat transfer. Recently, combining the functions of MOST and PCMs into a single component material (MOST-PCM) has been utilized to add storage capacity to the MOST system since the charging of the system is not only happening *via* solar irradiation but also by taking energy directly from the environment.^18–21^ This dual input leads to an increased energy density by almost 100%.^19^ Another attractive feature is added to PCMs; since the solidification of the *cis* liquid is not happening spontaneously, the phase change is locked by the photochemical system. This feature dramatically extends the functionality of the MOST-PCM combination since the phase change is controlled by external stimuli and no insulation is needed to hold the latent heat.

However, a severe limitation of MOST-PCMs based on azo-molecules studied until now is their inability to be charged and discharged in the solid state in cold environments, especially below 0 °C, because of the high melting point (*T*_m_) of *cis*-isomers. This is a critical condition since many applications such as thermo regulated fabrics,^22^ or functional coatings will need to be able to function at that temperature.^23^ Generally, the *trans*–*cis* photoisomerization of azo molecules requires a large free volume^24,25^ and can only occur in the surface layers of *trans*-crystals, thus preventing the charging process in the neat solid state. If the ambient temperature exceeds the *cis*-isomer *T*_m_, the generated *cis*-isomer melts into a liquid and exposes new *trans*-crystal surfaces, and finally the *trans*-crystals are entirely transformed into *cis*-liquids. But most reported *T*_m_ values of *cis*-isomers are in the range of 20–200 °C,^26^ which means that their photoisomerization from *trans*-crystals to *cis*-liquid cannot occur at low ambient temperatures. On the other hand, although some *cis*-isomers can maintain liquid states below 0 °C due to their supercooling behavior to achieve discharging at low temperature, the charging process is still at room temperature (27 °C), limiting the versatility of the system.^19,20^

Another challenge for the MOST-PCM is that the charging process generally needs UV light irradiation, since UV light causes damage to materials and the human body, and comprises a small fraction (4.5%) of the total solar spectrum,^27^ resulting in the low utilization efficiency of solar energy. To date, only one study has reported the utilization of *ortho*-functionalized azobenzene derivatives to store both visible light energy and room temperature ambient heat. However, this system could not be charged below 0 °C, and the energy density was in the 0.07–0.15 MJ kg^−1^ range.^28^*ortho*-Substitution can increase the energy of *trans*-isomers^29^ or decrease the energy of *cis*-isomers,^30^ so that the Δ*H*_iso_ of this type of azo molecule decreases to only 6–25 kJ mol^−1^ (0.01–0.05 MJ kg^−1^).^28^ Therefore, a reversibly charging/discharging and visible-light-energy storage MOST-PCM working at low-temperature remains to be explored.

**Fig. 1:**
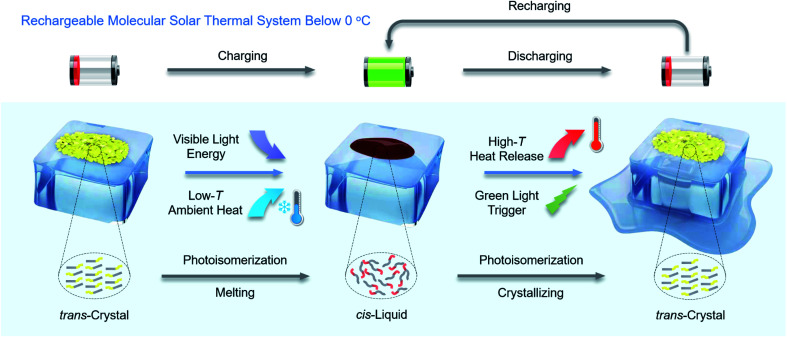
Schematic representation of a rechargeable molecular solar thermal system below 0 °C, which stores energy from both visible light energy and low-temperature ambient heat, and then discharges it on demand in the form of high-temperature heat.

Here, we report new arylazopyrazoles as MOST-PCMs, which are rechargeable below 0 °C by visible light, as illustrated in Fig. 1. In addition, by co-harvesting the visible light energy and low-temperature ambient heat, an energy density of 0.25 MJ kg^−1^ is achieved, which is an increase of 67% over previous comparable systems.^28^ Furthermore, the *cis*-isomer has a half-life of 22 days at 0 °C, demonstrating its stable energy storage capacity. The combination of high energy density, storage time, and the fact that the system can be charged at low temperatures provides the opportunity to explore the function of the material in a new type of optically regulated MOST-PCM window as a proof-of-concept study, designed to illustrate the function of the MOST-PCM system in a coating. Glass coated with arylazopyrazoles is prepared as a miniature energy storage window. One point to highlight is that the novel windows can be charged and discharged at −1 °C by 400 nm blue light and 532 nm green light, respectively. During the discharging process, the surface temperature of the window can reach from −1 °C up to 21.7 °C (a temperature increase of 22.7 °C), corresponding to a thermal power output of 256.2 W m^−2^ during a continuous period of 60 s. Charging and discharging energy at low temperatures has potential implications for functional clothing,^31^ advanced sunglasses, deicing^23^ and home heating^32^ under ice-cold conditions, thereby increasing thermal comfort and reducing the energy consumption of conventional heating.

## Results and discussion

### Synthesis and UV-Vis absorption spectra of bidirectional visible-light-driven photoswitches

**Scheme 1:**
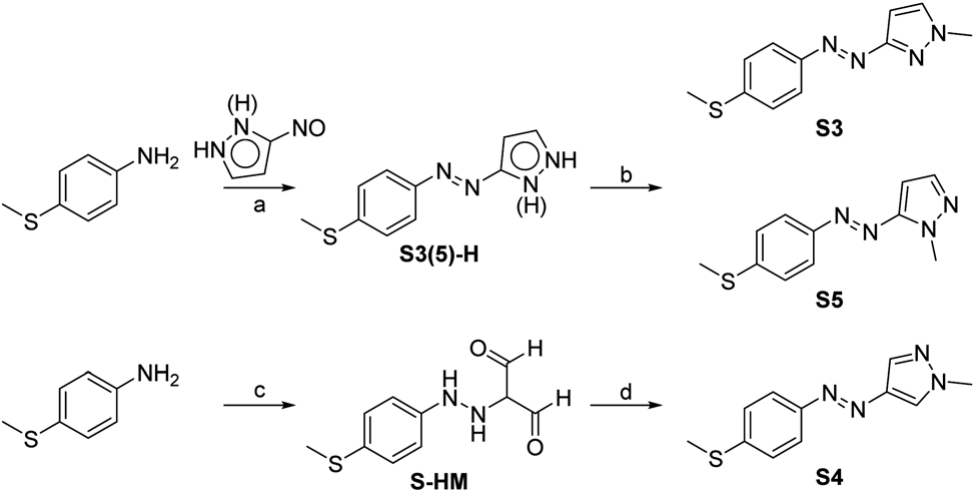
Synthesis routes of 4-methylthioarylazopyrazoles. Reagent and conditions. (a) AcOH, room temperature, 58%. (b) CH_3_I, CS_2_CO_3_, DMF, room temperature, 89%. (c) AcOH, concentrated HCl, aqueous NaNO_2_, 0–5 °C, aqueous KOAc, malonaldehyde sodium salt (MDA-Na), 0–5 °C, 54%. (d) Methylhydrazine sulfate, KOAc, EtOH, reflux, 74%.

The fundamental principle to realizing low-temperature working and visible-light energy storing MOST-PCM systems is to design photochromic molecules that drive phase transition with visible light at low temperature. The introduction of a 4-thiomethyl group and changing the bridging positions between the azo group and pyrazole ring was expected to achieve bidirectional visible light switching through extending the π-conjugation. Accordingly, three 4-methylthioarylazopyrazoles (S3, S4, and S5) were designed and their synthetic routes are shown in Scheme 1.

In previous studies,^33^ arylazopyrazoles were usually prepared by the Mills reaction of nitrosobenzene analogs and aminopyrazoles, but in this method it was difficult to synthesize arylazopyrazoles with electron-donating groups because the electron-donating groups would destabilize nitrosobenzene analogs.^34^ Hence, we first prepared 3(5)-nitroso-1*H*-pyrazole, which was then coupled with aniline analogs to give S3(5)-H. S3 and S5 were subsequently produced in one pot by *N*-methylation at two selectable positions, benefitting from the proton transfer and tautomerism of 1*H*-pyrazole. S4 was formed *via* diazo-coupling and cyclization reactions.

**Fig. 2:**
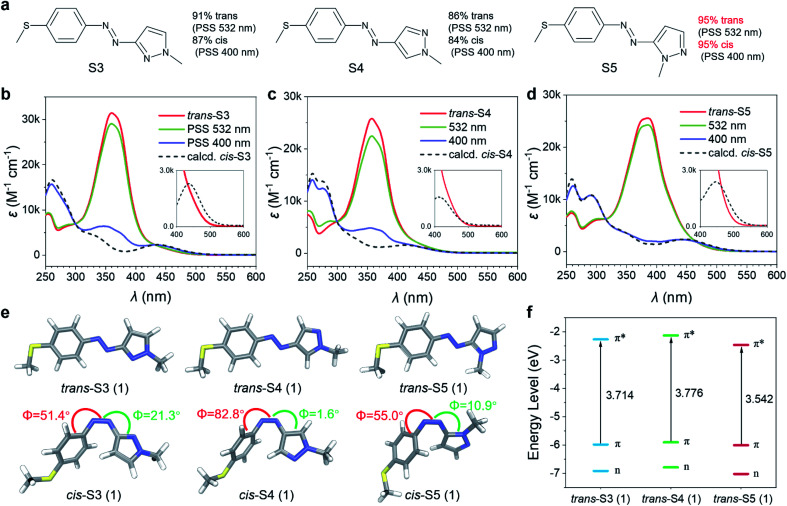
(a) Structures of 4-methoxyarylazopyrazole and their PSSs at 400 nm and 532 nm. Photoisomerization of (b) S3, (c) S4, and (d) S5 measured in acetonitrile, and the insets show the zoomed-in spectra of n–π* regions. (e) The geometrically optimized structure of 4-methoxyarylazopyrazole (calculations were performed at the PBE0-D3(BJ)/6-31G** level of theory in gas). (f) Calculated energy diagram of the π, n, and π* orbitals.

Their photoisomerization behaviors were studied using UV-Vis absorption spectra in an acetonitrile solution. As shown in Fig. 2b–d, all *trans*-isomers exhibited single and intense absorption bands in the 350–400 nm region (*ε*_max_ = 25–32 × 10^3^ M^−1^ cm^−1^) due to π–π* transition. Compared to the reported 4-methoxyarylazopyrazole (O4, 342 nm),^35^ the π–π* *λ*_max_ of S3 and S4 were red-shifted to about 360 nm, and further to 385 nm for S5 (Table S1†). The *trans*/*cis* relative absorption of S3, S4 and S5 at 400 nm was strong (Table S1†), and hence it was possible to realize *trans*–*cis* isomerization using visible light. The photostationary states (PSSs) at different wavelengths (from 365 to 532 nm) were studied (Fig. S1†), and the isomeric compositions are presented in Table S2.† As a result, 400 nm blue light induced a near-quantitative yield (>95%) of *trans*–*cis* isomerization for S5, while only ∼85% for S3 and S4. The higher *trans*–*cis* photoconversion of S5 was attributed to its π–π* *λ*_max_ closer to 400 nm and stronger *trans*/*cis* relative absorption at *λ* = 400 nm. Exciting the tail of n–π* bands of three *cis*-isomers using green light (532 nm) resulted in *cis*–*trans* isomerization. The high overlap between the n–π* band of *cis*-S4 and the long-wavelength absorption band of *trans*-S4 led to a relatively low *cis*–*trans* conversion (85%). In contrast to *cis*-S4, *cis*-S3 and *cis*-S5 exhibited n–π* transitions red-shifted to 25 nm and 36 nm (Table S1†), respectively, which led to partial separation of the n–π* bands of the *cis* and *trans* isomers, thereby inducing high (91% S3) to near-quantitative (>95% S5) isomerization.

### Theoretical analysis of bidirectional visible-light-driven photoswitches

The geometries of O4, S3, S4, and S5 and their electronic transition characteristics were calculated using density functional theory (DFT) modelling^36^ to evaluate the relationship between molecular structures and photophysical properties (see Section 3 in the ESI†). All *trans*-isomers exhibited a planar structure with a C–N–N–C dihedral angle of 180.0° (Fig. 2e and S5†), which resulted in symmetry-forbidden n–π* transitions (S_0_ → S_1_) with negligible oscillator strength (*f* = 0.00, Table S4†). Compared to *trans*-O4, the 4-methylthioarylazopyrazole series showed more effective extension of the π-conjugated system due to the increase of p–π conjugation by introducing the 4-SMe group,^37–39^ as indicated by their frontier molecular orbitals (Fig. S7–S10†). Consequently, the energy gap between the HOMO (π orbital) and LUMO (π* orbital) was smaller for the 4-methylthioarylazopyrazole series (3.714, 3.776, and 3.542 eV for S3, S4, and S5, respectively, as depicted in Fig. 2f), leading to a bathochromic shift of their π–π* transition (S_0_ → S_2_). The increased red-shift of S5 was caused by a “complete” conjugation pathway between 5-pyrazole and the azo group that further expands the π-conjugation of the system.^40,41^*cis*-S4 showed a nearly T-shaped conformation with a C–C–N–N dihedral angle of 82.8° (Fig. 2e and S5†), resulting in a weak n–π* transition with *f* of only 0.0048. In contrast, *cis*-S3 and *cis*-S5 were found to disfavor the T-shaped conformation owing to the presence of the *ortho* nitrogen atom (Fig. 2e and S5†). Therefore, their n–π* absorbance bands were remarkably enhanced (*f* = 0.1068 and 0.1096 for *cis*-S3 and *cis*-S5, respectively, Table S4†), and nearly quantitative conversions from *cis* to *trans* isomers were achieved.

### Design of low-temperature charging/discharging MOST-PCMs

**Fig. 3:**
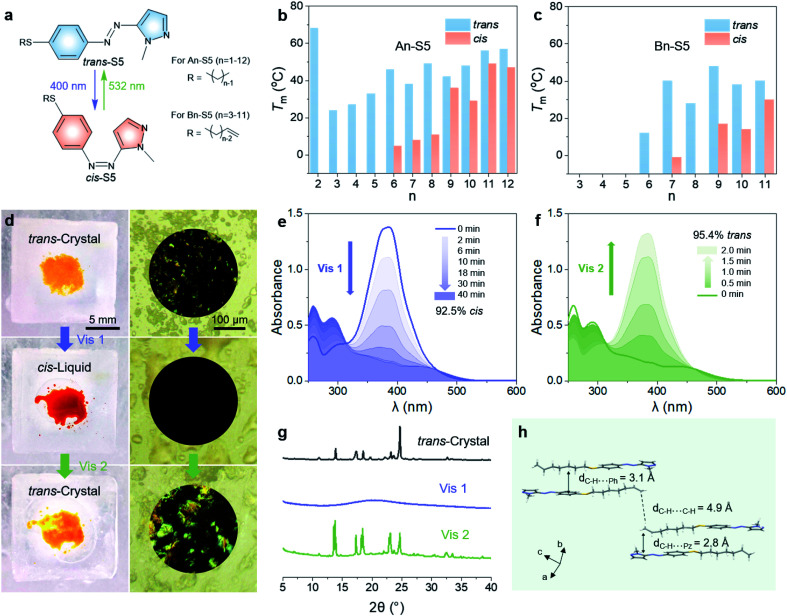
(a) The structures of An-S5 and Bn-S5. The *T*_m_ of *trans* and *cis* isomers of (b) An-S5 and (c) Bn-S5. (d) Photographs, optical microscopy images, and POM image of B7-S5 on the ice surface. From top to bottom, the panels are *trans*-crystals before irradiation, *cis*-liquids after 400 nm light (40 mW cm^−2^) irradiation, and regenerated *trans*-crystals after subsequent irradiation by 532 nm light (110 mW cm^−2^). The UV-Vis absorption spectra change during (e) 400 nm (40 mW cm^−2^) and (f) 532 nm (110 mW cm^−2^) light irradiation of neat B7-S5 at −1 °C. (g) XRD pattern change of B7-S5 on glass slides during the phase transition at −1 °C. (h) Crystal packing structure of *trans*-B7-S5.

Thus, all the above data indicate that S5 can be used as a bidirectional visible-light-driven photoswitch, which provided near-quantitative *trans*–*cis* and *cis*–*trans* photoconversions in acetonitrile solution. However, pristine S5 was not able to store both visible-light energy and phase transition latent heat at low temperature since the photoisomerization from *trans*-crystals to *cis*-liquid was inhibited even at room temperature. To charge and discharge energy below 0 °C, two additional principles are considered: (i) the *T*_m_ of the *cis*-isomer should be below 0 °C, thus forming amorphous *cis*-liquids to store photon energy and ambient heat; (ii) the *trans*-isomer should have a *T*_m_ and crystallization point (*T*_cry_) much higher than 0 °C, thus forming *trans*-crystals to release energy. In order to adjust the *T*_m_ of the *trans* and *cis* isomers, we varied the length of linear alkyl chains with or without a vinylic end group on the thioalkyl group of S5, denoted as An-S5 and Bn-S5 (Fig. 3a and Scheme S1†). Studying the phase behaviour of the systems using differential scanning calorimetry (DSC), we find that the *trans*-isomers with longer and intermediate alkyl chain lengths (*n* = 6–12 for An-S5, and *n* = 7, 9, and 11 for Bn-S5) showed *T*_m_ (40–60 °C) and *T*_cry_ above 0 °C (10–45 °C, Tables S5 and S6†). The *cis*-isomers with intermediate alkyl chain lengths had the lowest *T*_m_ (5 °C for A6-S5 and −1 °C for B7-S5), as shown in Fig. 3b, c and S13, S14.† Hence, B7-S5 could undergo a reversible visible-light-triggered *trans*-crystal ↔ *cis*-liquid transition in cold environments thanks to its low *cis*-isomer *T*_m_ (below 0 °C) and high *trans*-isomer *T*_cry_ (34 °C). This property illustrates, to the best of our knowledge, the first example of a functional low-temperature visible-light controlled energy storage MOST-PCM system.

### Charging/discharging below 0 °C

The charging and discharging processes of B7-S5 at −1 °C are discussed as follows. As shown in Fig. 3d and Video S1,† after 400 nm light (40 mW cm^−2^) irradiation, *trans*-B7-S5 in the orange crystal state lost birefringence and melted into red liquids, which then switched back to a crystal state by 532 nm light (110 mW cm^−2^) irradiation. UV-Vis spectroscopy was used to record the photoisomerization yields during irradiation of a neat sample of B7-S5 with a mass of 2 mg and a thickness of about 50 μm. During the charging process (Fig. 3e), the *trans*–*cis* isomerization of B7-S5 in a neat state proceeded easily and produced a high yield of photoisomerization (93%) at 40 min, slightly lower than that in dilute solutions (>95%). During the discharging process (Fig. 3f), the *trans*-isomer content of the sample increased exponentially, and a near-quantitative (95%) *cis*–*trans* isomerization was achieved within 2 min of irradiation. X-ray diffraction (XRD) analyses were carried out to further understand and verify the reversible *trans*-crystal ↔ *cis*-liquid behavior of B7-S5 (Fig. 3g). The sharp peaks at 2*θ* of 5–35° before irradiation corresponded to the regular stack of azo molecules in *trans*-crystals. After exposure to 400 nm light (40 mW cm^−2^), the peaks disappeared, indicating that *cis*-B7-S5 had an amorphous structure. Subsequently, XRD patterns were recovered when the sample was irradiated with 532 nm light (110 mW cm^−2^). The X-ray crystal structure of *trans*-B7-S5 reveals an antiparallel packing, and several weak contacts such as alkyl–alkyl, alkyl–phenyl, and alkyl–pyrazolyl dominating the intermolecular interactions (Fig. 3e). Presumably, the absence of strong intermolecular interactions in *trans*-B7-S5 offered enough flexibility to the system, which was beneficial for the charging process.^42,43^

### Energy storage time and energy density

**Fig. 4:**
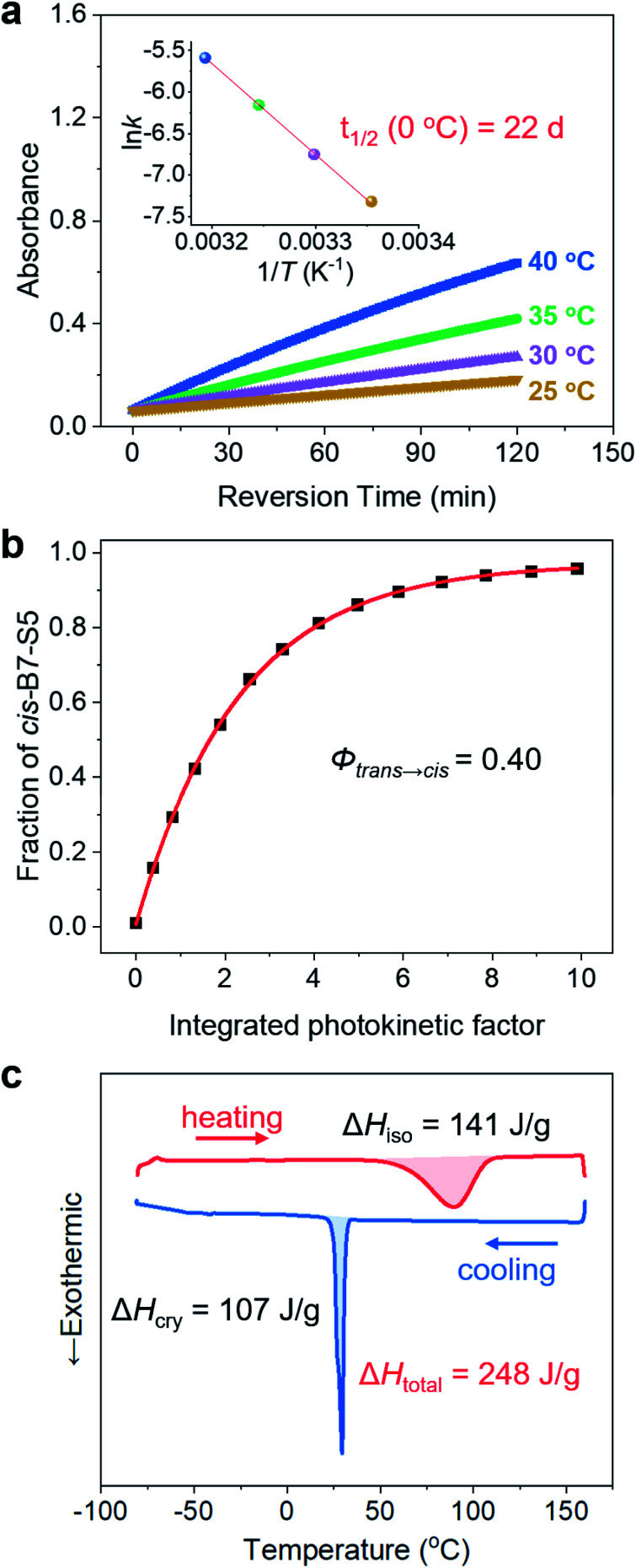
(a) Thermal *cis*–*trans* recovery of *cis*-B7-S5 in acetonitrile with time at different temperatures followed by the absorbance increase at *λ*_max_ = 385 nm. The inset shows the linear fit between ln *k* and 1/*T* according to the Arrhenius equation. (b) Exponential fitting of the fractions of *cis*-B7-S5 against the integrated photokinetic factor *x*(*t*). (c) First heating (red) and subsequent cooling DSC curves (blue) of *cis*-B7-S5.

The *cis*-isomers of azo molecules can thermally relax into *trans*-isomers in the dark spontaneously, which determines the energy storage stability of *cis*-B7-S5. The change of absorbance value at π–π* *λ*_max_ (385 nm) as a function of time was measured in acetonitrile solution between 25 and 40 °C (Fig. 4a and S15†). The thermal isomerization rate constants, *k*_*cis*→*trans*_ (Table S8†), were calculated based on the first-order reaction kinetics at 25 °C, 30 °C, 35 °C, and 40 °C, respectively. Based on the Arrhenius equation, *cis*-B7-S5 was found to have a half-life *t*_1/2_ of 22.4 days at 0 °C, indicating its stable thermal energy storage capacity at low temperatures. Thermal *cis* → *trans* kinetics was also studied in neat states (Fig. S16 and Table S9†), and *cis*-B7-S5 liquid still had a *t*_1/2_ as long as 6.3 days at 0 °C. It is ubiquitous to observe a lower *t*_1/2_ of photoswitches in condensed states than in solution. *Cis*-isomers tend to adopt fully relaxed geometries in solution, whereas in condensed states different geometries may be preferred due to intermolecular interactions, characterized by lower isomerization barriers.^44^ Additionally, B7-S5 demonstrated excellent photon-harvesting ability with a quantum yield *Φ*_*trans*→*cis*_ of 0.39 ± 0.01 for photoisomerization in acetonitrile solution (Fig. 4b and S17†), similar to other reported azopyrazoles compounds.^19,41,45^

To evaluate the energy density of the MOST-PCM, the isomerization enthalpy Δ*H*_iso_ of the thermally induced *cis*-liquid to *trans*-liquid reversion reaction and crystallization enthalpy Δ*H*_cry_ of the *trans*-liquid to *trans*-crystal transition were measured by DSC. As shown in Fig. 4c, the *cis*-B7-S5 liquid revealed a broad exothermic peak over 60–120 °C during the thermally activated *cis*–*trans* isomerization, and the integrated area under the peak represented a Δ*H*_iso_ of 0.14 MJ kg^−1^ (44 kJ mol^−1^). This result was consistent with our calculations (49 kJ mol^−1^, Table S7†) based on DFT and similar to the pristine azobenzene (<50 kJ mol^−1^). Furthermore, the DSC cooling curve displayed a sharp exothermic peak at around 33 °C with a Δ*H*_cry_ of 0.11 MJ kg^−1^ (35 kJ mol^−1^), which was due to the *trans*-liquid to *trans*-crystal transition. Therefore, the total thermal energy density of the MOST-PCM was 0.25 MJ kg^−1^ (79 kJ mol^−1^). According to *Φ*_*trans*→*cis*_ and Δ*H*_iso_, the solar efficiency *η* was estimated to be up to 1.3% (see Section 6 in the ESI†), which was one of the highest values reported for azo-based MOST systems (0.2–1.3%).^19,28^

### Rechargeable energy storage coatings

**Fig. 5:**
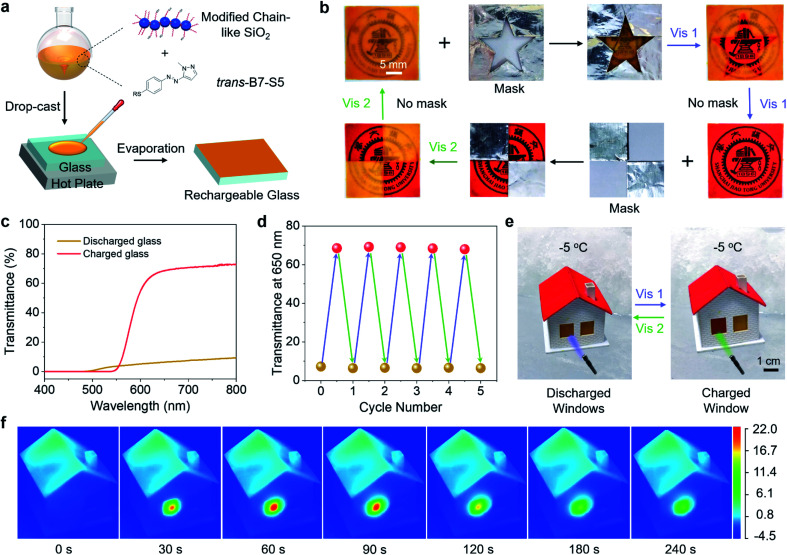
(a) Schematic illustration of the fabrication of rechargeable glass *via* drop-casting. (b) Photographs of the rechargeable glass sheet with photo-controlled transparency. (c) Transmittance spectra for charged and discharged glass. (d) The transmittance change of rechargeable glass at 650 nm during five charging/discharing cycles. (e) Photographs of the house model with rechargeable glass sheets installed as energy storage windows, and charing/discharing below 0 °C (Vis 1 = 400 nm light with an intensity of 40 mW cm^−2^ and Vis 2 = 532 nm light with an intensity of 110 mW cm^−2^). (f) Time-evolved infrared images of the charged windows under 532 nm light (110 mW cm^−2^) irradiation at −1 °C. A maximum heat release temperature up to 22.7 °C was recorded.

To illustrate the practical applications of B7-S5, *trans*-B7-S5 and diethoxydimethylsilane modified chain-like silica were co-dissolved in ethanol and isopropanol (1 : 1, v/v) solution, and then the mixture was drop-cast onto a glass substrate to form rechargeable coatings, as shown in Fig. 5a. The modified chain-like silica formed a transparent porous network structure^46^ on glass substrates to prevent the leakage of *cis*-liquid (Fig. S18†). Various patterns were created on the same rechargeable glass substrate through selectively writing/erasing processes (Fig. 5b). First, a mask was placed on the glass and irradiated with 400 nm blue light. The exposed area of the glass was transformed from the initial opacity to semi-transparency, and the color changed from orange to red. As a result, the target pattern was written on the glass substrate. Subsequently, the erasing process could happen when the substrate without a mask was exposed to 400 nm blue light irradiation, resulting in a globally semi-transparent glass. And then, this glass substrate could be further patterned *via* selectively irradiating with 532 nm green light. Finally, the glass recovered to its opaque state after exposure to 532 nm green light without a mask. The transmittance spectra of the rechargeable glass under different light irradiations were recorded (Fig. 5c). The glass sheet in a discharged state had a low transmittance of less than 10% in the wavelength range of 500–800 nm, and after exposure to 400 nm blue light, the glass sheet is charged with the transmittance up to ∼70% in the wavelength range of 650–800 nm. In addition, the rechargeable glass showed good durability under alternating blue light and green light irradiations (Fig. 5d).

Thanks to the high transmittance of the charged state glass sheet and the low-temperature phase transition of B7-S5, the rechargeable glass had the potential for use as a photochromic solar thermal energy storage window in daily life, especially in cold winters. As a proof-of-principle study, the rechargeable glass sheets were installed on a miniature house model as windows (size 10 by 12 mm and coating thickness ∼400 μm). The house model was placed in −5 °C surroundings to ensure that the surface temperature of the windows was around −1 °C. As shown in Fig. 5e, upon exposure to 400 nm blue light (40 mW cm^−2^), the window stored visible light energy and low-temperature ambient heat while transforming from opacity to semi-transparency. Then, by triggering it with 532 nm green light (110 mW cm^−2^), the stored energy was rapidly released on demand as high-temperature heat.

A high-resolution infrared thermal imaging camera was used to track the temperature changes of the window when exposed to 400 nm (40 mW cm^−2^) and 532 nm light (110 mW cm^−2^) (Fig. 5f, S19 and Videos S2–S5†). During the 400 nm light irradiation (charging process), the window exhibited a temperature difference of about 3 °C above the ambient temperature, indicating a weak photothermal effect. The charged window reached 21.7 °C at 60 s during the 532 nm light irradiation (discharging process), about 22.7 °C higher than the cold surroundings. A control experiment of irradiating the discharged window with 532 nm light showed a much lower temperature change (9.3 °C), which means that the temperature change between the charged window and environment was mainly due to *cis*–*trans* isomerization. Assuming that the *cis*–*trans* isomerization was fully completed at approximately 60 s, the corresponding thermal power output was estimated to be 256.2 W m^−2^. Such high-temperature heat release also means that the B7-S5 molecules on the surface of the window can act as a photon-driven molecular heat pump, upgrading thermal energy from low to high temperature. Furthermore, the optically controlled heat release makes it possible to reach about an order of magnitude higher temperature gradients than is possible with traditional MOST window coating concepts.^32^ These solar thermal energy storage coatings show unprecedented performances, including visible-light trigger/storage, high energy density, and recyclable ice-cold charging/discharging, thus holding great promise for future energy management systems.

## Conclusions

In conclusion, we have successfully designed a series of bidirectional visible-light switching azo molecules and applied them as MOST-PCMs for storing and releasing solar energy below 0 °C. The molecular design strategies are summarized as follows: (i) the 4-thioalkyl substituent on azo molecules shifts the π–π* absorption bands to long-wavelength, enabling bidirectional visible light photoisomerization; (ii) replacing one phenyl ring on the azo molecules with a pyrazole ring increases the half-life of the metastable *cis*-isomer; (iii) varying the length of thioalkyl chains changes the intermolecular forces, which could adjust the *T*_m_ of both *trans* and *cis* isomers. Eventually, reversible visible-light-triggered *trans*-crystal ↔ *cis*-liquid transitions are achieved below 0 °C. Accordingly, the azo molecules can simultaneously store visible-light energy and low-temperature ambient heat to achieve a high energy density (0.25 MJ kg^−1^).

Moreover, a rechargeable coating is prepared by drop-coating a solution containing azo photoswitches and modified chain-like silica on the glass surface. The coating shows potential as energy storage windows due to optical transmittance in the charged state, but it is also clear that more work is needed to increase the optical transmittance of the material. Future studies could focus on red-shifting *λ*_max_ of azo molecules to the near-infrared region to fabricate efficient semitransparent energy storage windows. Other possible application areas are functional coatings and fabrics with controllable heat release functions. We note that the structure–property relations derived from the chemical design provide a blue-print for how to design future MOST-PCM systems with tailored temperature functions and optimised optical properties. We envision that this work can open an avenue for the design of advanced MOST-PCM systems that store natural sunlight and ambient heat over a wide temperature range.

## Data availability

All experimental procedures and data related to this study can be found in the ESI.†

## Author contributions

T. L. and K. M. P. conceived the study and supervised the project. Z. S. was primarily responsible for the experiments. W. S., Z.-Y. Z. and D. F. helped synthesize the compounds. S. W., R.-Z. W. and T.-X. L. provided the infrared thermal imaging camera characterization and analysis. Z.-Y. Z., Z. W. and C. D. contributed to the visualization. X. H. performed the XRD data analysis. Y. H. contributed to DFT calculations. All authors contributed to the writing of the manuscript.

## Conflicts of interest

The authors declare no competing interests.

## Supplementary Material

SC-013-D2SC01873J-s001

SC-013-D2SC01873J-s002

SC-013-D2SC01873J-s003

SC-013-D2SC01873J-s004
