# Supplementary material for: A rechargeable molecular solar thermal system below 0 °C
Source: Chem Sci. 2022 May 16;13(23):6950–8. doi: 10.1039/d2sc01873j (PMC9200126; doi:10.1039/d2sc01873j)
Supplement: SC-013-D2SC01873J-s001 [file SC-013-D2SC01873J-s001.pdf]

Electronic Supplementary Material (ESI) for Chemical Science.
This journal is © The Royal Society of Chemistry 2022

Supporting Information

Rechargeable Molecular Solar Thermal System Below 0 °C

Zhichun Shangguan,[a] Wenjin Sun,[a] Zhao-Yang Zhang,[a] Dong Fang,[a] Zhihang Wang,[b] Si Wu,[c] Chao Deng,[d] Xianhui Huang,[a] Yixin He,[a] Ruzhu Wang,[c] Tingxian Li,[c] Kasper Moth-Poulsen*[b], Tao Li*[a]

[a]*School of Chemistry and Chemical Engineering, Frontiers Science Center for Transformative Molecules, Shanghai Key Laboratory of Electrical Insulation and Thermal Aging, Key Laboratory of Thin Film and Microfabrication (Ministry of Education), Shanghai Jiao Tong University, Shanghai 200240, China. *E-mail: litao1983@sjtu.edu.cn*

[b]*Department of Chemistry and Chemical Engineering, Chalmers University of Technology, Gothenburg 41296, Sweden. *E-mail: kasper.moth-poulsen@chalmers.se*

[c]*Research Center of Solar Power & Refrigeration, School of Mechanical Engineering, Shanghai Jiao Tong University, Shanghai 200240, China.*

[d]*College of Chemistry & Materials Engineering, Wenzhou University, Wenzhou 325027, Zhejiang, China.*

Table of contents

1. Synthesis 1
2. Photochemical characterization 8
3. Computational details 13
4. DSC results for An-S5 and Bn-S5 21
5. Thermal isomerization kinetics and half-life 26
6. Quantum yield and solar energy conversion efficiency 29
7. Preparation of rechargeable glass 30
8. Infrared image 31
9. Single crystal X-ray diffraction 32
10. NMR spectra 34
11. Cartesian coordinates for optimized geometries 62
12. References 78

1. Synthesis

1.1 General methods

All chemicals were purchased from Adamas-beta, Bide Pharmatech Ltd., Sigma-Aldrich and Sinopharm Chemical Reagent Co., Ltd. and used without further purification, unless otherwise mentioned. Malonaldehyde sodium salt (MDA-Na) was synthesized according to a previous literature.[1]

^{1}H and ^{13}C spectra were recorded on Bruker AVANCE III HD (400 MHz) spectrometer. Chemical shifts for protons are reported in parts per million (ppm) downfield from tetramethylsilane and are referenced to residual proton in the NMR solvent ($CHCl_3$: δ 7.26, CH_3CN 1.94, DMSO 2.50). Chemical shifts for carbon are reported in ppm downfield from tetramethylsilane and are referenced to the carbon resonances of the solvent ($CDCl_3$: δ 77.0, DMSO 39.5). Data are represented as follows: chemical shift, multiplicity (s = singlet, d = doublet, t = triplet, m = multiplet, br = broad), coupling constants in Hertz, and integration. **HRMS** data were obtained on Bruker Impact II quadrupole time of flight mass spectrometry instrument.

1.2 Synthetic procedures

3-nitroso-1H-pyrazole (1-2)

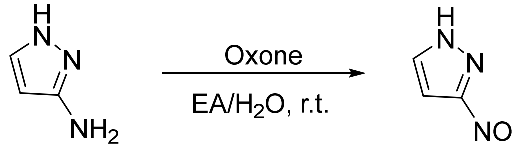

1-1 **1-2**

A mixture of compound **1-1** (4.15 g, 50 mmol, 1.0 equiv.) and $NaHCO_3$ (8.4 g, 100 mmol, 2.0 equiv.) in EtOAc (200 mL) and H_2O (400 mL) was stirred at room temperature, and then Oxone (30.74 g, 50 mmol, 1.0 equiv.) was added to the mixture in portions. The reaction was stirred at room temperature for 1 h and separated in an organic layer. The aqueous layer was extracted with EtOAc, and the combined organic layers were dried over Na_2SO_4, filtered, and concentrated by evaporation to give a gray green solid (2.50 g, 51.5% yield). The crude product **1-2** was directly used in the next step without future purification.

(*E*)-3-((4-(methylthio)phenyl)diazenyl)-1H-pyrazole (1-3)

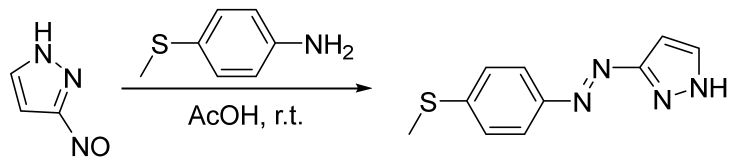

1-2 **1-3**

A mixture of compound **1-2** (500 mg, 5.15 mmol, 1.0 equiv.) and 4-(methylthio)aniline (716 mg, 5.15 mmol, 1.0 equiv.) in AcOH (20 mL) was stirred overnight at room temperature. The resulting mixture was concentrated by evaporation, and then recrystallized with EtOAc and hexane to afford the product **1-3** as yellow solids (651 mg, 58.0% yield). ^{1}H NMR (400 MHz, $CDCl_3$) δ 7.88 (d, J = 8.3 Hz, 2H), 7.66 (d, J = 2.4 Hz, 1H), 7.34 (d, J = 8.4 Hz, 2H), 6.76 (d, J = 2.5 Hz, 1H), 2.55 (s, 3H). ^{13}C NMR (101 MHz, $CDCl_3$) δ 161.71, 149.85, 143.63, 132.78, 125.85, 123.47, 97.36, 15.20. HRMS (ESI) *m/z* calculated for $[C_{10}H_{10}N_4S+H]^+$ 219.0699, found 219.0697.

(*E*)-1-methyl-3-((4-(methylthio)phenyl)diazenyl)-1H-pyrazole (S3)

(*E*)-1-methyl-5-((4-(methylthio)phenyl)diazenyl)-1H-pyrazole (S5)

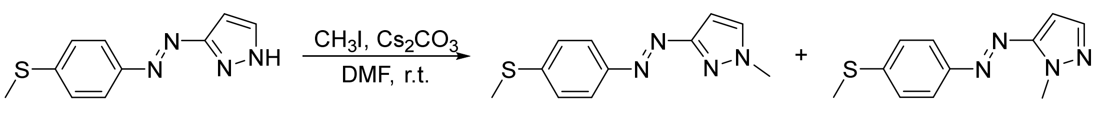

1-3 **S3** **S5**

A mixture of compound **1-3** (500 mg, 2.29 mmol, 1.0 equiv.), CH_3I (650 mg, 4.58 mmol, 2.0 equiv.) and Cs_2CO_3 (1.49 g, 4.58 mmol, 2.0 equiv.) in DMF (5 mL) was stirred at room temperature for 4 h then quenched with H_2O. The reaction mixture was extracted with EtOAc, dried over Na_2SO_4, filtered, and concentrated by evaporation. The crude residue was purified by silica gel chromatography (PE/EtOAc 8:1) to give pure product **S3** (228 mg, 42.9% yield) and **S5** (247 mg, 46.5% yield) as orange yellow solids.

S3: ^{1}H NMR (400 MHz, $CDCl_3$) δ 7.89 (d, *J* = 8.7 Hz, 2H), 7.38 (d, *J* = 2.4 Hz, 1H), 7.32 (d, *J* = 8.7 Hz, 2H), 6.63 (d, *J* = 2.4 Hz, 1H), 4.01 (s, 3H), 2.54 (s, 3H). ^{13}C NMR (101 MHz, $CDCl_3$) δ 163.71, 150.04, 142.99, 131.79, 125.85, 123.41, 96.15, 39.65, 15.24. HRMS (ESI) *m/z* calculated for $[C_{11}H_{12}N_4S+H]^+$ 233.0855, found 233.0856.

S5: ^{1}H NMR (400 MHz, $CDCl_3$) δ 7.83 (d, *J* = 8.6 Hz, 2H), 7.55 (d, *J* = 2.2 Hz, 1H), 7.33 (d, *J* = 8.6 Hz, 2H), 6.53 (d, *J* = 2.2 Hz, 1H), 4.22 (s, 3H), 2.56 (s, 3H). ^{13}C NMR (101 MHz, $CDCl_3$) δ 153.21, 150.18, 143.89, 139.17, 125.84, 123.35, 93.62, 36.08, 15.18. HRMS (ESI) *m/z* calculated for $[C_{11}H_{12}N_4S+H]^+$ 233.0855, found 233.0858.

2-(2-(4-(methylthio)phenyl)hydrazineyl)malonaldehyde (2-2)

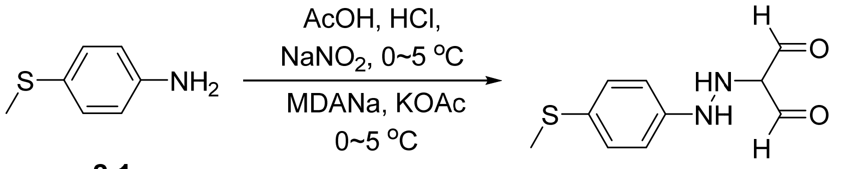

2-1 **2-2**

Compound **2-1** (1.39 g, 10 mmol, 1.0 equiv.) was dissolved in concentrated HCl (2.3 mL) and AcOH (15 mL) then the solution was cooled to 5-10 °C. $NaNO_2$ (1.04 g, 15 mmol, 1.5 equiv.) solution was added dropwise and kept the temperature of the reaction mixture below 10 °C. After stirred for 30 min at 5 °C, a cold solution of KOAc (2.94 g, 30 mmol, 3.0 equiv.) and MDA-Na (1.43 g, 15 mmol, 1.5 equiv.) was added in sequence then the mixture was stirred at 5 °C for another 2 h. The resulting precipitate was filtered, washed with water. The filter cake was dried under vacuum, affording the product **2-2** as an orange red solid (1.20 g, 54.1% yield). ^{1}H NMR (400 MHz, $CDCl_3$) δ 14.70 (s, 1H), 9.96 (s, 1H), 9.61 (s, 1H), 7.44 (d, *J* = 8.8 Hz, 2H), 7.31 (d, *J* = 8.8 Hz, 2H), 2.52 (s, 3H). ^{13}C NMR (101 MHz, $CDCl_3$) δ 189.60, 186.54, 138.25, 137.91, 133.21, 127.55, 117.38, 15.88. HRMS (ESI) *m/z* calculated for $[C_{10}H_{10}N_2O_2S+Na]^+$ 245.0355, found 245.0355.

(*E*)-1-methyl-4-((4-(methylthio)phenyl)diazenyl)-1H-pyrazole (S4)

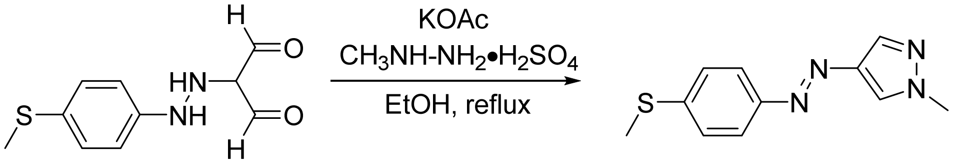

2-2 **S4**

A mixture of compound **2-2** (1.00 g, 4.5 mmol, 1.0 equiv.), methylhydrazine sulfate (778 mg, 5.4 mmol, 1.2 equiv.) and KOAc (1.06 g, 10.8 mmol, 2.4 equiv.) in EtOH (50 mL) was stirred for 4 h

at reflux, then quenched with water and then extracted with EtOAc. The organic phase was washed with brine, dried over Na_2SO_4, filtered, and concentrated by evaporation. The crude residue was purified by silica gel chromatography (PE/EtOAc 5:1) to give pure product **S4** as a yellow solid (769 mg, 73.7% yield). ^{1}H NMR (400 MHz, $CDCl_3$) δ 8.00 (s, 1H), 7.94 (s, 1H), 7.73 (d, J = 8.6 Hz, 2H), 7.31 (d, J = 8.6 Hz, 2H), 3.97 (s, 3H), 2.54 (s, 3H). ^{13}C NMR (101 MHz, $CDCl_3$) δ 150.27, 141.91, 141.74, 133.38, 126.56, 126.12, 122.71, 39.61, 15.41. HRMS (ESI) *m/z* calculated for $[C_{11}H_{12}N_4S+H]^+$ 233.0855, found 233.0854.

Synthesis routes of An-S5 and Bn-S5

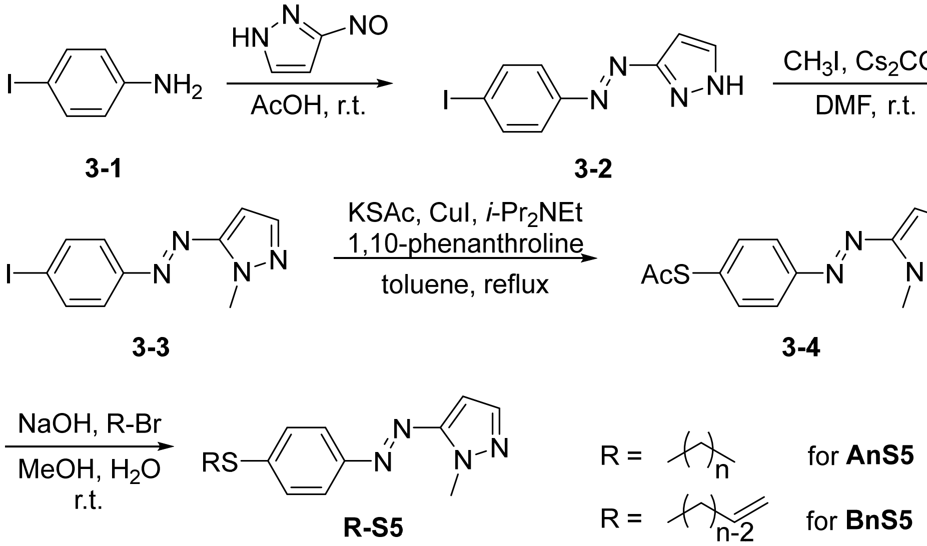

Scheme S1. Synthesis routes of An-S5 and Bn-S5.

(*E*)-3-((4-iodophenyl)diazenyl)-1H-pyrazole (3-2)

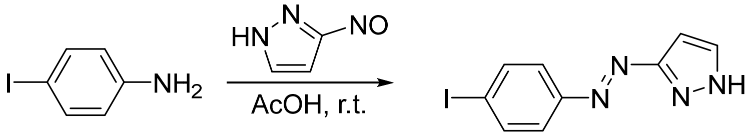

3-1 **3-2**

A mixture of compound **3-1** (4.15 g, 50 mmol, 1.0 equiv.) and 4-iodoaniline (8.4 g, 100 mmol, 2.0 equiv.) in AcOH (100 mL) was stirred overnight at room temperature. The resulting precipitate was filtered, washed with water. The filter cake was dried under vacuum, affording the product **3-2** as a yellow solid (7.39 g, 49.6%). ^{1}H NMR (400 MHz, DMSO) δ 13.54 (s, 1H), 7.97 (d, J = 8.5 Hz, 2H), 7.90 (s, 1H), 7.64 (d, J = 8.6 Hz, 2H), 6.58 (s, 1H). ^{13}C NMR (101 MHz, DMSO) δ 163.78, 151.49, 138.39, 130.77, 124.11, 98.44, 93.61. HRMS (ESI) *m/z* calculated for $[C_9H_7IN_4+H]^+$ 298.9788, found 298.9784.

(*E*)-5-((4-iodophenyl)diazenyl)-1-methyl-1H-pyrazole (3-3)

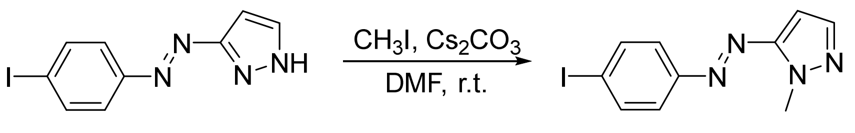

3-2 **3-3**

A mixture of compound **3-2** (5.0 g, 23.6 mmol, 1.0 equiv.), CH_3I (6.70 g, 47.2 mmol, 2.0 equiv.) and Cs_2CO_3 (6.5 g, 47.2 mmol, 2.0 equiv.) in DMF (50 mL) was stirred at room temperature for 4 h then quenched with H_2O. The reaction mixture was extracted with EtOAc, dried over Na_2SO_4,

filtered, and concentrated by evaporation. The crude residue was purified by silica gel chromatography (PE/EtOAc 10:1) to give pure product **3-3** as an orange yellow solid (3.48 g, 47.2% yield). ^{1}H NMR (400 MHz, $CDCl_3$) δ 7.86 (d, *J* = 8.6 Hz, 2H), 7.62 (d, *J* = 8.7 Hz, 2H), 7.56 (d, *J* = 2.2 Hz, 1H), 6.56 (d, *J* = 2.3 Hz, 1H), 4.23 (s, 3H). ^{13}C NMR (101 MHz, $CDCl_3$) δ 152.87, 152.00, 139.15, 138.34, 124.25, 98.27, 94.01, 36.10. HRMS (ESI) *m/z* calculated for $[C_{10}H_9IN_4+H]^+$ 312.9945, found 312.9942.

(*E*)-S-(4-((1-methyl-1H-pyrazol-5-yl)diazenyl)phenyl) ethanethioate (3-4)

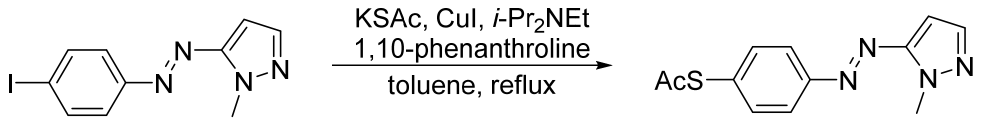

A mixture of compound **3-3** (3.12 g, 10 mmol, 1.0 equiv.), KSAc (1.37 g, 12 mmol, 1.2 equiv.), 1,10-phenanthroline (360 mg, 2 mmol, 0.2 equiv.) and CuI (190 mg, 1 mmol, 0.1 equiv.) in toluene (50 mL) and N,N-Diisopropylethylamine (5 mL) was stirred overnight at reflux under a nitrogen atmosphere. The reaction mixture was filtered, and the filtrate was concentrated under reduced pressure. The crude residue was purified by silica gel chromatography (PE/EtOAc 5:1) to give pure product **3-4** as an orange yellow solid (1.73 g, 66.5% yield). ^{1}H NMR (400 MHz, $CDCl_3$) δ 7.91 (d, *J* = 8.7 Hz, 2H), 7.59 – 7.52 (m, 3H), 6.57 (d, *J* = 2.3 Hz, 1H), 4.23 (s, 3H), 2.46 (s, 3H). ^{13}C NMR (101 MHz, $CDCl_3$) δ 193.08, 153.09, 152.94, 139.25, 134.95, 131.62, 123.34, 94.08, 36.17, 30.40. HRMS (ESI) *m/z* calculated for $[C_{10}H_9IN_4+H]^+$ 261.0805, found 261.0802.

(*E*)-5-((4-(alkylthio)phenyl)diazenyl)-1-methyl-1H-pyrazole (An-S5)

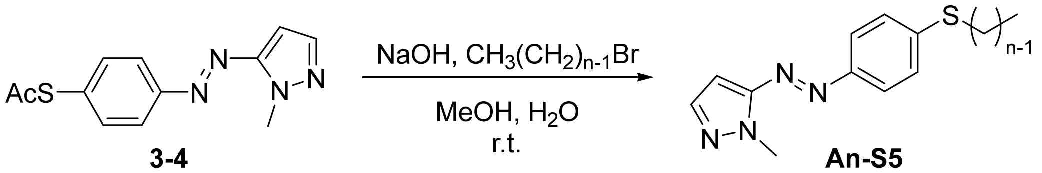

A mixture of compound **3-4** (260 mg, 1 mmol, 1.0 equiv.) and NaOH (400 mg, 10 mmol, 10.0 equiv.) in MeOH (60 mL) and H_2O (5 mL) was stirred at room temperature for 30 min, and then R-Br (2 mmol, 2.0 equiv.) was stirred to the mixture. After stirred overnight, water was added to quench the reaction, and then extracted with DCM. The organic phase was washed with brine, dried over Na_2SO_4, filtered, and concentrated by evaporation. The crude residue was purified by silica gel chromatography (PE/EtOAc 8:1) to give pure product as orange yellow solids (88 – 92% yield).

A2-S5: ^{1}H NMR (400 MHz, $CDCl_3$) δ 7.82 (d, *J* = 8.5 Hz, 2H), 7.55 (d, *J* = 2.3 Hz, 1H), 7.37 (d, *J* = 8.6 Hz, 2H), 6.53 (d, *J* = 2.2 Hz, 1H), 4.22 (d, *J* = 1.1 Hz, 3H), 3.04 (q, *J* = 7.4 Hz, 2H), 1.39 (t, *J* = 7.3 Hz, 3H). ^{13}C NMR (101 MHz, $CDCl_3$) δ 153.19, 150.41, 142.53, 139.16, 127.36, 123.35, 93.64, 36.07, 26.56, 14.03. HRMS (ESI) *m/z* calculated for $[C_{12}H_{14}N_4S+H]^+$ 247.1012, found 247.1013.

A3-S5: ^{1}H NMR (400 MHz, $CDCl_3$) δ 7.81 (d, *J* = 8.7 Hz, 2H), 7.54 (d, *J* = 2.2 Hz, 1H), 7.36 (d, *J* = 8.7 Hz, 2H), 6.53 (d, *J* = 2.2 Hz, 1H), 4.21 (s, 3H), 2.99 (t, *J* = 7.4 Hz, 2H), 1.82 – 1.68 (m, 2H), 1.07 (t, *J* = 7.4 Hz, 3H). ^{13}C NMR (101 MHz, $CDCl_3$) δ 153.18, 150.35, 142.84, 139.13, 127.32, 123.32, 93.63, 36.05, 34.43, 22.26, 13.50. HRMS (ESI) *m/z* calculated for $[C_{13}H_{16}N_4S+H]^+$ 261.1168, found 261.1169.

A4-S5: ^{1}H NMR (400 MHz, $CDCl_3$) δ 7.81 (d, *J* = 8.7 Hz, 2H), 7.54 (d, *J* = 2.2 Hz, 1H), 7.36 (d, *J* = 8.6 Hz, 2H), 6.53 (d, *J* = 2.2 Hz, 1H), 4.21 (s, 3H), 3.01 (t, *J* = 7.4 Hz, 2H), 1.77 – 1.65 (m, 2H), 1.54 – 1.42

(m, 2H), 0.96 (t, *J* = 7.3 Hz, 3H). ^{13}C NMR (101 MHz, $CDCl_3$) δ 153.20, 150.34, 142.95, 139.14, 127.26, 123.34, 93.63, 36.05, 32.14, 30.89, 22.04, 13.64. HRMS (ESI) *m/z* calculated for $[C_{14}H_{18}N_4S+H]^+$ 275.1325, found 275.1324.

A5-S5: ^{1}H NMR (400 MHz, $CDCl_3$) δ 7.81 (d, *J* = 8.7 Hz, 2H), 7.54 (d, *J* = 2.2 Hz, 1H), 7.36 (d, *J* = 8.6 Hz, 2H), 6.53 (d, *J* = 2.2 Hz, 1H), 4.21 (s, 3H), 3.00 (t, *J* = 7.4 Hz, 2H), 1.76 – 1.68 (m, 2H), 1.51 – 1.31 (m, 4H), 0.92 (t, *J* = 7.2 Hz, 3H). ^{13}C NMR (101 MHz, $CDCl_3$) δ 153.19, 150.33, 142.94, 139.14, 127.25, 123.33, 93.62, 36.06, 32.42, 31.06, 28.53, 22.24, 13.95. HRMS (ESI) *m/z* calculated for $[C_{15}H_{20}N_4S+H]^+$ 289.1481, found 289.1479.

A6-S5: ^{1}H NMR (400 MHz, $CDCl_3$) δ 7.81 (d, *J* = 8.7 Hz, 2H), 7.54 (d, *J* = 2.2 Hz, 1H), 7.36 (d, *J* = 8.6 Hz, 2H), 6.53 (d, *J* = 2.2 Hz, 1H), 4.21 (s, 3H), 3.01 (t, *J* = 7.4 Hz, 2H), 1.78 – 1.66 (m, 2H), 1.51 – 1.42 (m, 2H), 1.29 – 1.35 (m, 4H), 0.90 (t, *J* = 7.2 Hz, 3H). ^{13}C NMR (101 MHz, $CDCl_3$) δ 153.20, 150.34, 142.95, 139.15, 127.26, 123.33, 93.63, 36.07, 32.47, 31.34, 28.80, 28.59, 22.52, 14.01. HRMS (ESI) *m/z* calculated for $[C_{16}H_{22}N_4S+H]^+$ 303.1638, found 303.1637.

A7-S5: ^{1}H NMR (400 MHz, $CDCl_3$) δ 7.81 (d, *J* = 8.6 Hz, 2H), 7.54 (d, *J* = 2.2 Hz, 1H), 7.36 (d, *J* = 8.7 Hz, 2H), 6.53 (d, *J* = 2.2 Hz, 1H), 4.21 (s, 3H), 3.00 (t, *J* = 7.4 Hz, 2H), 1.75 – 1.68 (m, 2H), 1.51 – 1.41 (m, 2H), 1.37 – 1.22 (m, 6H), 0.89 (t, *J* = 7.4 Hz, 3H). ^{13}C NMR (101 MHz, $CDCl_3$) δ 153.19, 150.34, 142.95, 139.14, 127.25, 123.33, 93.62, 36.06, 32.46, 31.68, 28.87, 28.84, 28.83, 22.58, 14.07. HRMS (ESI) *m/z* calculated for $[C_{17}H_{24}N_4S+H]^+$ 317.1794, found 317.1794.

A8-S5: ^{1}H NMR (400 MHz, $CDCl_3$) δ 7.82 (d, *J* = 8.6 Hz, 2H), 7.55 (d, *J* = 2.2 Hz, 1H), 7.36 (d, *J* = 8.6 Hz, 2H), 6.53 (d, *J* = 2.2 Hz, 1H), 4.22 (s, 3H), 3.01 (t, *J* = 7.4 Hz, 2H), 1.76 – 1.68 (m, 2H), 1.54 – 1.41 (m, 2H), 1.34 – 1.24 (m, 8H), 0.88 (t, *J* = 7.4 Hz, 3H). ^{13}C NMR (101 MHz, $CDCl_3$) δ 153.21, 150.36, 142.96, 139.16, 127.28, 123.34, 93.63, 36.07, 32.49, 31.79, 29.15, 29.13, 28.92, 28.85, 22.64, 14.10. HRMS (ESI) *m/z* calculated for $[C_{18}H_{26}N_4S+H]^+$ 331.1951, found 331.1952.

A9-S5: ^{1}H NMR (400 MHz, $CDCl_3$) δ 7.81 (d, *J* = 8.7 Hz, 2H), 7.54 (d, *J* = 2.2 Hz, 1H), 7.36 (d, *J* = 8.7 Hz, 2H), 6.53 (d, *J* = 2.2 Hz, 1H), 4.21 (s, 3H), 3.00 (t, *J* = 7.4 Hz, 2H), 1.75 – 1.68 (m, 2H), 1.51 – 1.41 (m, 2H), 1.34 – 1.20 (m, 10H), 0.88 (t, *J* = 7.4 Hz, 3H). ^{13}C NMR (101 MHz, $CDCl_3$) δ 153.20, 150.35, 142.96, 139.16, 127.27, 123.34, 93.63, 36.07, 32.48, 31.85, 29.45, 29.24, 29.17, 28.91, 28.84, 22.66, 14.11. HRMS (ESI) *m/z* calculated for $[C_{19}H_{28}N_4S+H]^+$ 345.2107, found 345.2106.

A10-S5: ^{1}H NMR (400 MHz, $CDCl_3$) δ 7.81 (d, *J* = 8.6 Hz, 2H), 7.54 (d, *J* = 2.2 Hz, 1H), 7.36 (d, *J* = 8.6 Hz, 2H), 6.53 (d, *J* = 2.2 Hz, 1H), 4.21 (s, 3H), 3.01 (t, *J* = 7.4 Hz, 2H), 1.75 – 1.68 (m, 2H), 1.51 – 1.40 (m, 2H), 1.34 – 1.20 (m, 12H), 0.88 (t, *J* = 6.8 Hz, 3H). ^{13}C NMR (101 MHz, $CDCl_3$) δ 153.20, 150.35, 142.96, 139.16, 127.27, 123.34, 93.63, 36.07, 32.48, 31.88, 29.53, 29.49, 29.30, 29.16, 28.91, 28.84, 22.68, 14.12. HRMS (ESI) *m/z* calculated for $[C_{20}H_{30}N_4S+H]^+$ 359.2264, found 359.2260.

A11-S5: ^{1}H NMR (400 MHz, $CDCl_3$) δ 7.82 (d, *J* = 8.7 Hz, 2H), 7.55 (d, *J* = 2.2 Hz, 1H), 7.36 (d, *J* = 8.7 Hz, 2H), 6.53 (d, *J* = 2.2 Hz, 1H), 4.22 (s, 3H), 3.01 (t, *J* = 7.4 Hz, 2H), 1.75 – 1.68 (m, 2H), 1.51 – 1.40 (m, 2H), 1.34 – 1.20 (m, 14H), 0.88 (t, *J* = 6.7 Hz, 3H). ^{13}C NMR (101 MHz, $CDCl_3$) δ 153.21, 150.36, 142.96, 139.16, 127.28, 123.34, 93.63, 36.07, 32.48, 31.90, 29.60, 29.58, 29.49, 29.33, 29.16, 28.91, 28.85, 22.69, 14.12. HRMS (ESI) *m/z* calculated for $[C_{21}H_{32}N_4S+H]^+$ 373.2420, found 373.2412.

A12-S5: ^{1}H NMR (400 MHz, $CDCl_3$) δ 7.82 (d, *J* = 8.7 Hz, 2H), 7.55 (d, *J* = 2.2 Hz, 1H), 7.36 (d, *J* = 8.6 Hz, 2H), 6.53 (d, *J* = 2.2 Hz, 1H), 4.22 (s, 3H), 3.01 (t, *J* = 7.4 Hz, 2H), 1.75 – 1.68 (m, 2H), 1.51 – 1.40 (m, 2H), 1.34 – 1.20 (m, 16H), 0.88 (t, *J* = 6.7 Hz, 3H). ^{13}C NMR (101 MHz, $CDCl_3$) δ 153.21, 150.36, 142.96, 139.16, 127.28, 123.34, 93.63, 36.07, 32.48, 31.91, 29.65, 29.63, 29.58, 29.49, 29.35, 29.16, 28.91, 28.85, 22.69, 14.12. HRMS (ESI) *m/z* calculated for $[C_{22}H_{34}N_4S+H]^+$ 387.2577, found

387.2575.

(*E*)-5-((4-(alkenylthio)phenyl)diazenyl)-1-methyl-1H-pyrazole (Bn-S5)

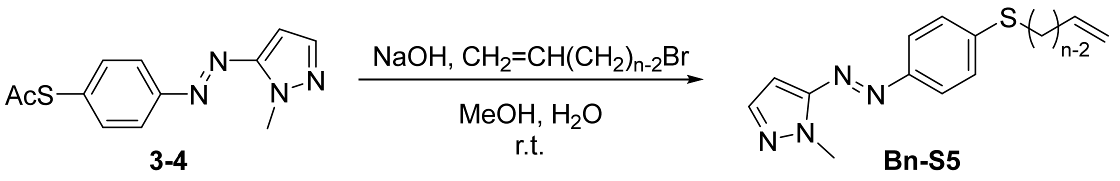

B3-S5: ^{1}H NMR (400 MHz, $CDCl_3$) δ 7.81 (d, *J* = 8.7 Hz, 2H), 7.55 (d, *J* = 2.2 Hz, 1H), 7.39 (d, *J* = 8.7 Hz, 2H), 6.53 (d, *J* = 2.2 Hz, 1H), 5.92 (ddt, *J* = 16.8, 10.0, 6.7 Hz, 1H), 5.20 – 5.04 (m, 2H), 4.21 (s, 3H), 3.66 (dt, *J* = 6.7, 1.3 Hz, 2H). ^{13}C NMR (101 MHz, $CDCl_3$) δ 153.16, 150.65, 141.58, 139.16, 132.79, 128.22, 123.26, 118.41, 93.69, 35.93. HRMS (ESI) *m/z* calculated for $[C_{13}H_{14}N_4S+H]^+$ 259.1012, found 259.1013.

B4-S5: ^{1}H NMR (400 MHz, $CDCl_3$) δ 7.82 (d, *J* = 8.6 Hz, 2H), 7.55 (d, *J* = 2.2 Hz, 1H), 7.38 (d, *J* = 8.6 Hz, 2H), 6.54 (d, *J* = 2.2 Hz, 1H), 5.88 (ddt, *J* = 16.9, 10.2, 6.6 Hz, 1H), 5.20 – 5.04 (m, 2H), 4.22 (s, 3H), 3.08 (t, *J* = 7.4 Hz, 2H), 2.53 – 2.42 (m, 2H). ^{13}C NMR (101 MHz, $CDCl_3$) δ 153.20, 150.52, 142.26, 139.18, 135.95, 127.62, 123.38, 116.68, 93.68, 36.09, 33.02, 31.88. HRMS (ESI) *m/z* calculated for $[C_{14}H_{16}N_4S+H]^+$ 273.1168, found 273.1167.

B5-S5: ^{1}H NMR (400 MHz, $CDCl_3$) δ 7.82 (d, *J* = 8.7 Hz, 2H), 7.55 (d, *J* = 2.3 Hz, 1H), 7.37 (d, *J* = 8.7 Hz, 2H), 6.54 (d, *J* = 2.2 Hz, 1H), 5.81 (ddt, *J* = 17.0, 10.2, 6.7 Hz, 1H), 5.13 – 4.99 (m, 2H), 4.22 (s, 3H), 3.02 (t, *J* = 7.3 Hz, 2H), 2.24 (dtd, *J* = 7.7, 6.7, 1.4 Hz, 2H), 1.90 – 1.76 (m, 2H). ^{13}C NMR (101 MHz, $CDCl_3$) δ 153.19, 150.43, 142.55, 139.16, 137.26, 127.46, 123.35, 115.74, 93.65, 36.07, 32.72, 31.78, 27.97. HRMS (ESI) *m/z* calculated for $[C_{15}H_{18}N_4S+H]^+$ 287.1325, found 287.1327.

B6-S5: ^{1}H NMR (400 MHz, $CDCl_3$) δ 7.81 (d, *J* = 8.7 Hz, 2H), 7.54 (d, *J* = 2.2 Hz, 1H), 7.36 (d, *J* = 8.7 Hz, 2H), 6.53 (d, *J* = 2.2 Hz, 1H), 5.80 (ddt, *J* = 16.9, 10.2, 6.7 Hz, 1H), 5.08 – 4.91 (m, 2H), 4.21 (s, 3H), 3.01 (t, *J* = 7.3 Hz, 2H), 2.15 – 2.04 (m, 2H), 1.78 – 1.70 (m, 2H), 1.63 – 1.51 (m, 2H). ^{13}C NMR (101 MHz, $CDCl_3$) δ 153.19, 150.38, 142.75, 139.15, 138.21, 127.33, 123.34, 114.93, 93.64, 36.06, 33.19, 32.31, 28.26, 28.04. HRMS (ESI) *m/z* calculated for $[C_{16}H_{20}N_4S+H]^+$ 301.1481, found 301.1475.

B7-S5: ^{1}H NMR (400 MHz, $CDCl_3$) δ 7.81 (d, *J* = 8.6 Hz, 2H), 7.55 (d, *J* = 2.2 Hz, 1H), 7.36 (d, *J* = 8.7 Hz, 2H), 6.53 (d, *J* = 2.2 Hz, 1H), 5.80 (ddt, *J* = 16.9, 10.1, 6.7 Hz, 1H), 5.06 – 4.90 (m, 2H), 4.21 (s, 3H), 3.01 (t, *J* = 7.4 Hz, 2H), 2.11 – 2.01 (m, 2H), 1.78 – 1.67 (m, 2H), 1.55 – 1.38 (m, 4H). ^{13}C NMR (101 MHz, $CDCl_3$) δ 153.20, 150.38, 142.81, 139.16, 138.64, 127.32, 123.34, 114.59, 93.64, 36.07, 33.57, 32.42, 28.68, 28.38, 28.33. HRMS (ESI) *m/z* calculated for $[C_{17}H_{22}N_4S+H]^+$ 315.1638, found 315.1638.

B8-S5: ^{1}H NMR (400 MHz, $CDCl_3$) δ 7.82 (d, *J* = 8.6 Hz, 2H), 7.55 (d, *J* = 2.2 Hz, 1H), 7.36 (d, *J* = 8.6 Hz, 2H), 6.53 (d, *J* = 2.2 Hz, 1H), 5.80 (ddt, *J* = 16.9, 10.1, 6.6 Hz, 1H), 5.04 – 4.90 (m, 2H), 4.22 (s, 3H), 3.01 (t, *J* = 7.4 Hz, 2H), 2.11 – 2.00 (m, 2H), 1.78 – 1.67 (m, 2H), 1.53 – 1.32 (m, 6H). ^{13}C NMR (101 MHz, $CDCl_3$) δ 153.20, 150.37, 142.87, 139.16, 138.90, 127.32, 123.34, 114.39, 93.63, 36.07, 33.66, 32.46, 28.78, 28.72, 28.61. HRMS (ESI) *m/z* calculated for $[C_{18}H_{24}N_4S+H]^+$ 329.1794, found 329.1793.

B9-S5: ^{1}H NMR (400 MHz, $CDCl_3$) δ 7.82 (d, *J* = 8.6 Hz, 2H), 7.55 (d, *J* = 2.3 Hz, 1H), 7.36 (d, *J* = 8.6 Hz, 2H), 6.53 (d, *J* = 2.2 Hz, 1H), 5.80 (ddt, *J* = 16.9, 10.1, 6.7 Hz, 1H), 5.05 – 4.89 (m, 2H), 4.21 (s, 3H), 3.01 (t, *J* = 7.3 Hz, 2H), 2.11 – 1.97 (m, 2H), 1.80 – 1.66 (m, 2H), 1.53 – 1.28 (m, 8H). ^{13}C NMR (101 MHz, $CDCl_3$) δ 153.20, 150.36, 142.90, 139.16, 139.05, 127.30, 123.34, 114.27, 93.63, 36.07, 33.73, 32.47, 28.99, 28.93, 28.83, 28.81. HRMS (ESI) *m/z* calculated for $[C_{19}H_{26}N_4S+H]^+$ 343.1951, found

343.1949.

B10-S5: ^{1}H NMR (400 MHz, $CDCl_3$) δ 7.81 (d, *J* = 8.7 Hz, 2H), 7.54 (d, *J* = 2.1 Hz, 1H), 7.35 (d, *J* = 8.6 Hz, 2H), 6.53 (d, *J* = 2.1 Hz, 1H), 5.80 (ddt, *J* = 16.9, 10.1, 6.6 Hz, 1H), 5.04 – 4.87 (m, 2H), 4.21 (d, *J* = 1.7 Hz, 3H), 3.00 (t, *J* = 7.4 Hz, 2H), 2.09 – 1.98 (m, 2H), 1.78 – 1.65 (m, 2H), 1.52 – 1.28 (m, 10H). ^{13}C NMR (101 MHz, $CDCl_3$) δ 153.19, 150.34, 142.92, 139.14, 139.11, 127.26, 123.33, 114.19, 93.62, 36.05, 33.76, 32.45, 29.30, 29.09, 29.01, 28.87, 28.81. HRMS (ESI) *m/z* calculated for $[C_{20}H_{28}N_4S+H]^+$ 357.2107, found 357.2107.

B11-S5: ^{1}H NMR (400 MHz, $CDCl_3$) δ 7.81 (d, *J* = 8.6 Hz, 2H), 7.54 (d, *J* = 2.2 Hz, 1H), 7.36 (d, *J* = 8.6 Hz, 2H), 6.53 (d, *J* = 2.2 Hz, 1H), 5.81 (ddt, *J* = 16.9, 10.1, 6.7 Hz, 1H), 5.05 – 4.88 (m, 2H), 4.21 (s, 3H), 3.00 (t, *J* = 7.4 Hz, 2H), 2.10 – 1.99 (m, 2H), 1.80 – 1.66 (m, 2H), 1.47 – 1.26 (m, 12H). ^{13}C NMR (101 MHz, $CDCl_3$) δ 153.20, 150.35, 142.94, 139.17, 139.15, 127.27, 123.33, 114.16, 93.63, 36.06, 33.79, 32.47, 29.42, 29.39, 29.13, 29.08, 28.90, 28.89, 28.83. HRMS (ESI) *m/z* calculated for $[C_{21}H_{30}N_4S+H]^+$ 371.2264, found 371.2261.

2. Photochemical characterization

UV-Vis absorption spectra were recorded on a Shimadzu UV-2700 spectrophotometer with slit width of 2.0 nm. The light sources used for photoisomerization are: 365 nm LED lamp (Nichia NVSU233B chip, 6 W), LED lamps (Cree Q5 chip) with different wavelengths (400 nm, 412 nm, 420 nm, 450 nm, 470 nm, 500 nm), 532 nm laser (YZ532D200-CGS125-JYWK).

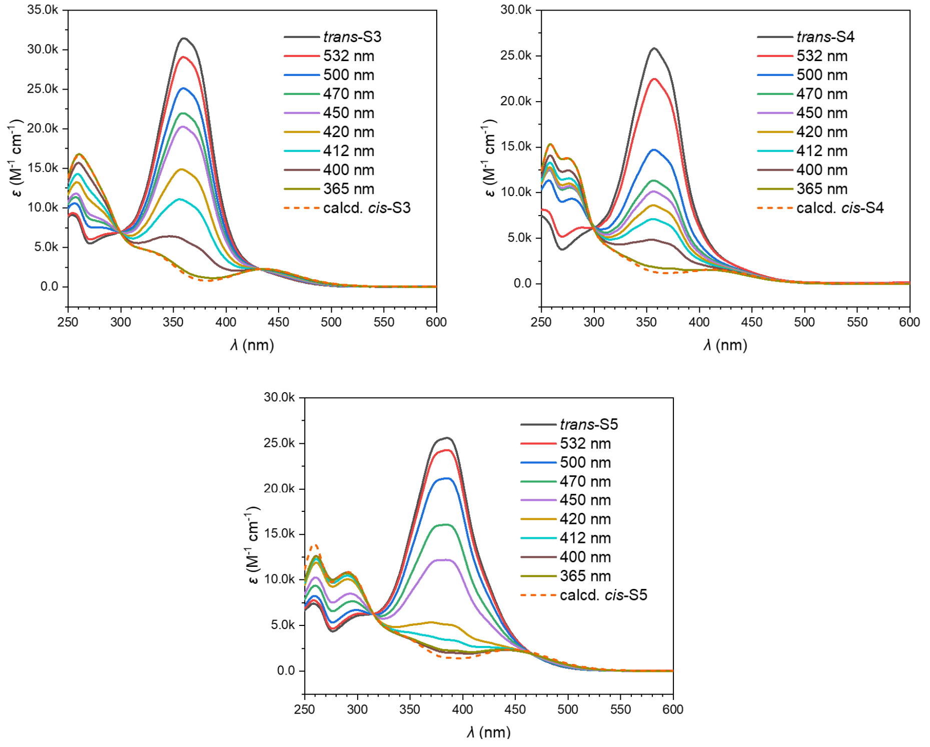

Figure S1. UV-Vis absorption spectra of **S3**, **S4**, an **S5** at different irradiation wavelengths in CH_3CN. The extracted spectra of *cis* isomers are shown as dotted lines.

Table S1. Spectroscopic data of **O4**, **S3**, **S4**, and **S5** in CH_3CN.

	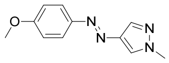 **O4**	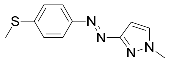 **S3**	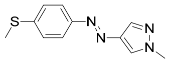 **S4**	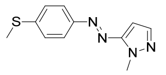 **S5**
trans π-π* λ_{max} (nm)	342.0	360.0	357.0	384.5
cis n-π* λ_{max} (nm)	415.0	436.0	412.5	446.0
$\varepsilon_{trans}/\varepsilon_{cis}$ at 400 nm	2.0	7.3	4.6	14.8

Quantification of PSS composition

First, the photostationary state (PSS) composition at 400 nm and 532 nm was quantified by integrating spectra peak areas of ^1H NMR spectra in CD_3CN of the *trans* and *cis* isomers. Then, the UV-Vis absorption spectra of the pure cis-isomers were calculated from the spectra of *trans*-isomers and 400 nm PSS. Finally, the isomer fractions of the other PSSs were obtained by spectral fitting using the *trans* and extracted *cis* isomers spectra, according to our previous work.[2-3]

Table S2. Calculated fraction of trans-isomers of **S3**, **S4**, and **S5** at different PSSs in CH_3CN.

	Fraction of *trans*-isomer at PSSs (%)		
	**S3**	**S4**	**S5**
365 nm	1.3	2.2	5.3
400 nm	13.6	17.2	4.8
412 nm	31.0	23.7	8.3
420 nm	44.0	29.8	15.1
450 nm	62.4	36.0	44.3
470 nm	68.2	40.9	60.4
500 nm	78.8	54.6	81.7
532 nm	90.9	85.5	95.2

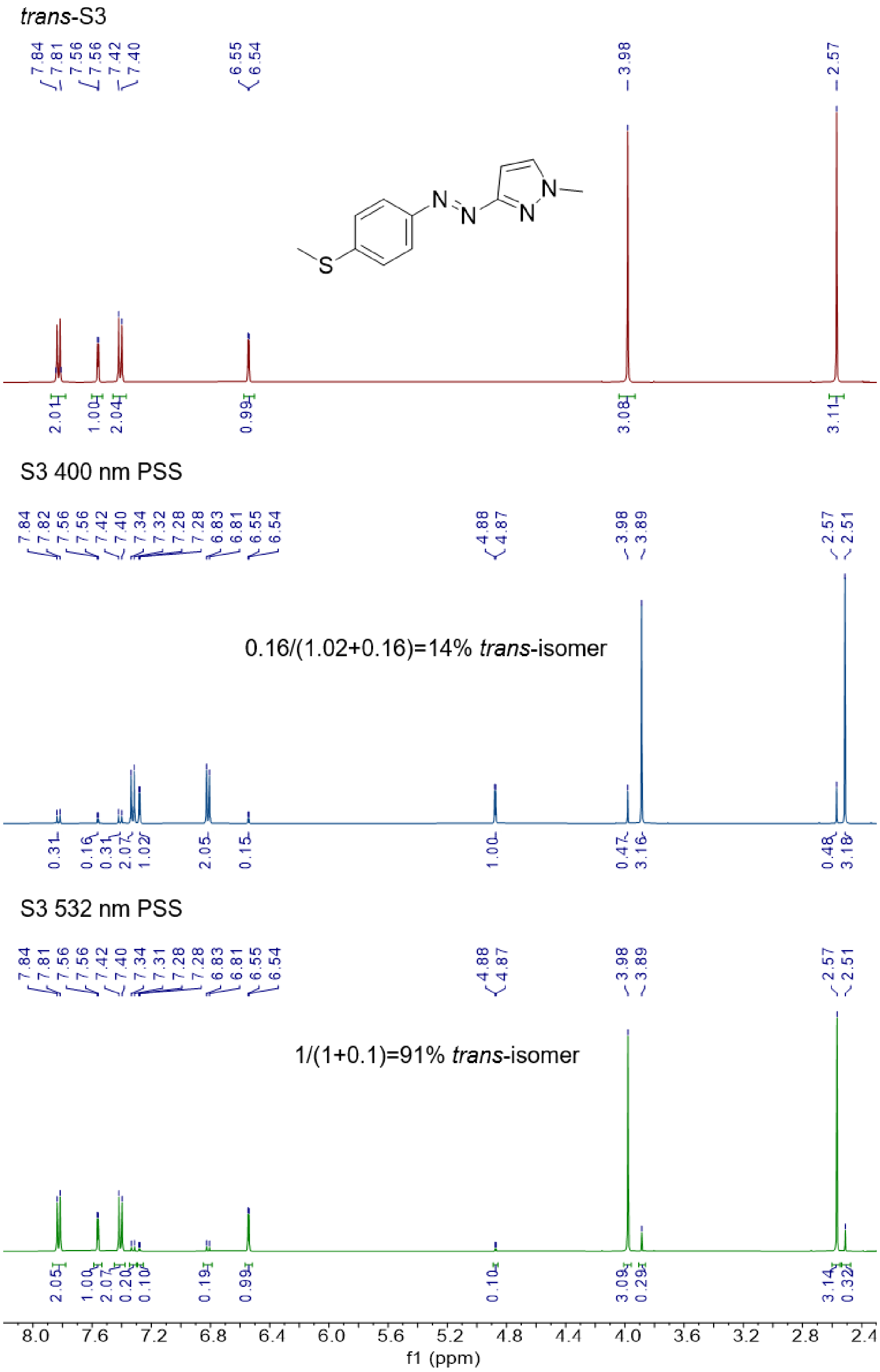

Figure S2. ^{1}H NMR spectra of *trans*-**S3** and its PSSs at 400 nm and 532 nm in CD_3CN.

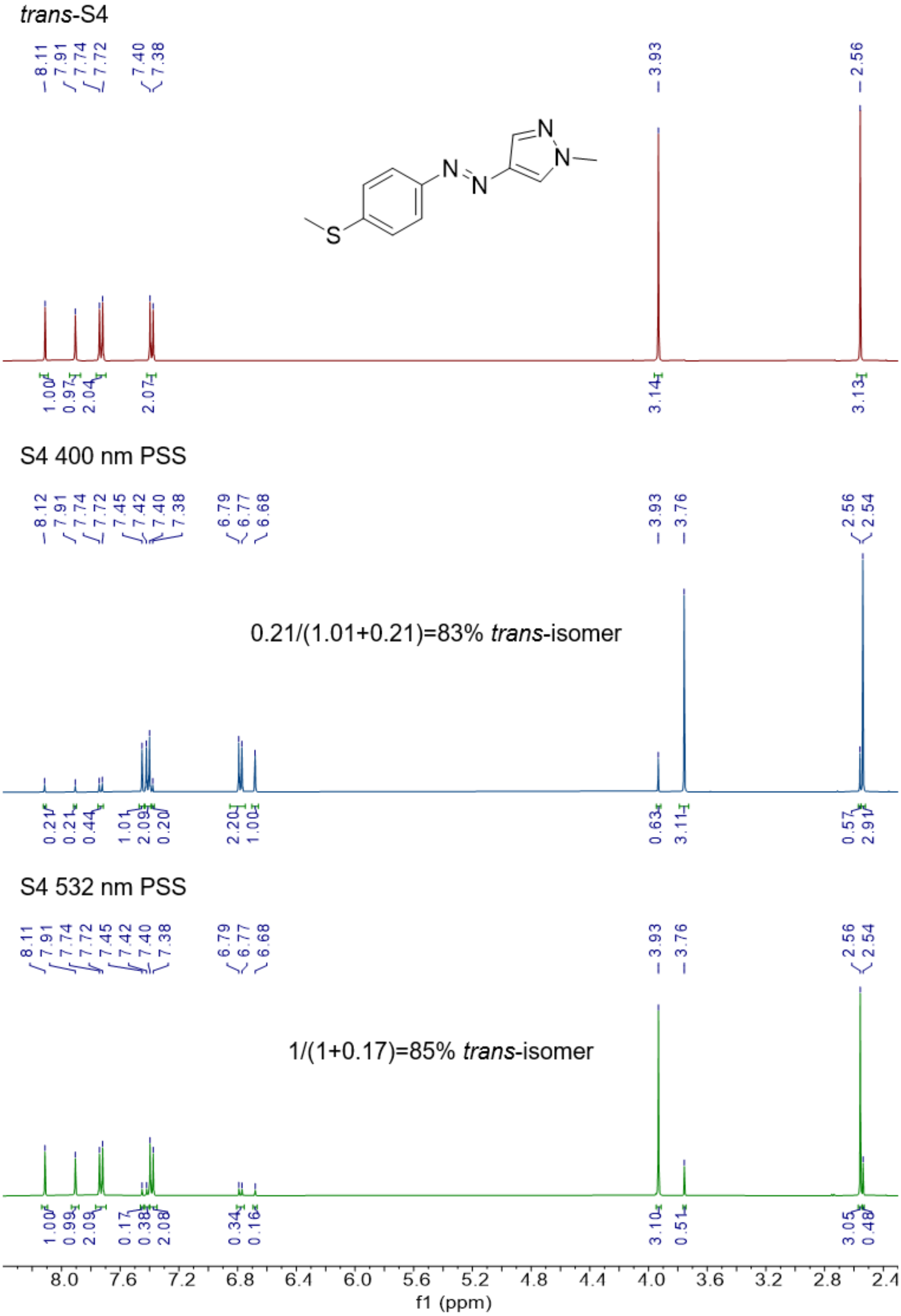

Figure S3. ^{1}H NMR spectra of *trans*-**S4** and its PSSs at 400 nm and 532 nm in CD_3CN.

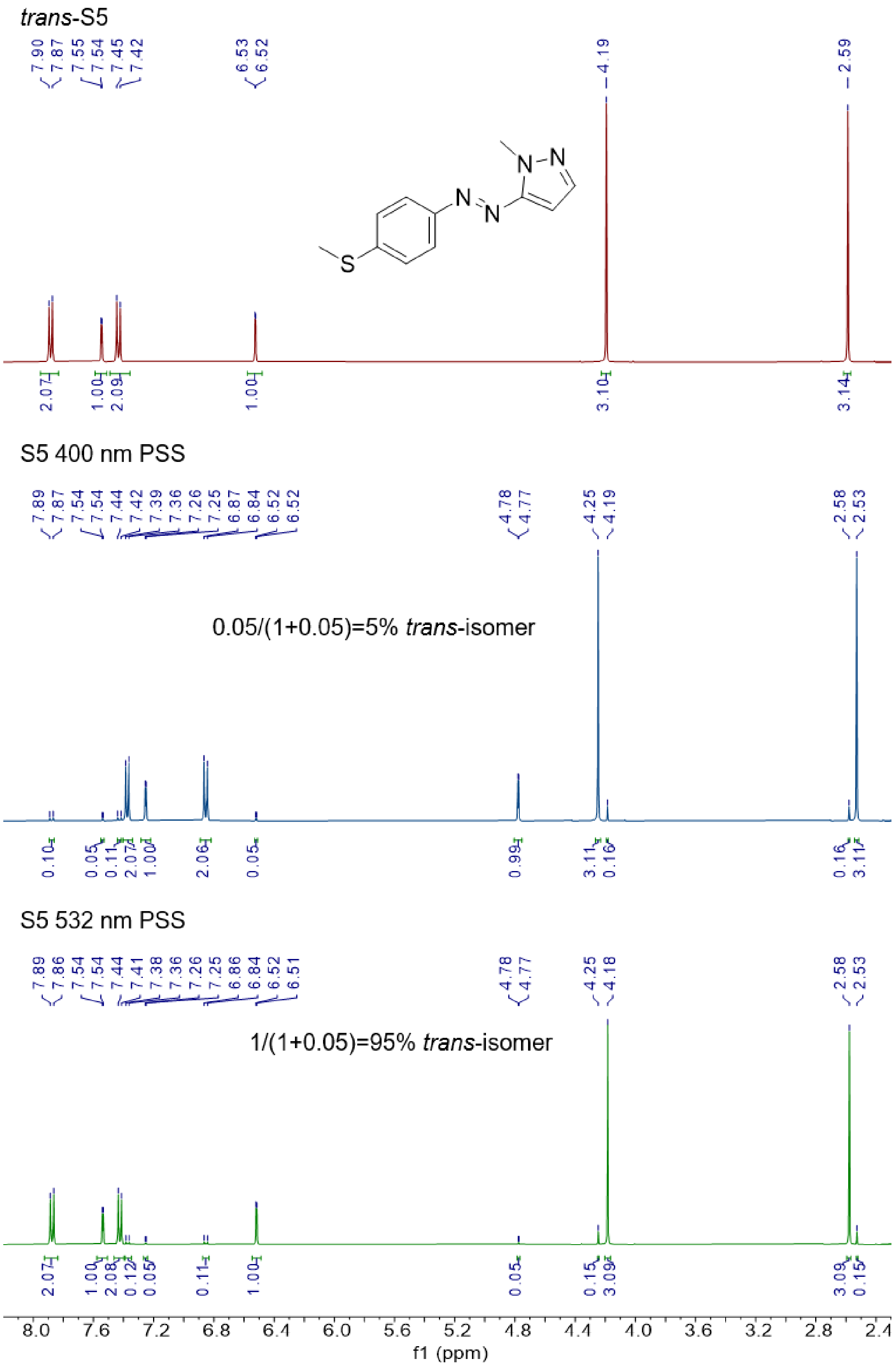

Figure S4. ^{1}H NMR spectra of *trans*-**S5** and its PSSs at 400 nm and 532 nm in CD_3CN.

3. Computational details

Since the aromatic ring rotates along C–N bonds linked to the azo group, two conformers were considered for all the molecules. The theoretical calculations were performed on ORCA 5.0 program.[4-5] The conformers for all azo molecules in the gas phase were optimized on the PBE0-D3(BJ)/def2-TZVP level of theory[6-8] with the RIJCOSX approximation[9], and then vibrational frequency analysis at the same theory level to ensure the reliability of the ground-state minimum geometry. The electronic energies of each conformer were computed at the PWPB95-D3(BJ)/def2-QZVPP level of theory[6-8] with the RIJCOSX approximation[9]. The solvation free energies (using SMD solvation model[10]) of each conformer in acetonitrile were computed at M06-2X/6-31G* level of theory[11-13]. Then, according to the relative Gibbs free energies, the Boltzmann distributions of each conformer in the gas phase and acetonitrile were obtained. The isomerization enthalpy energies between *trans* and *cis* isomers were calculated considering the conformation average. The vertical electronic excitation energies were calculated by time-dependent density functional theory (TDDFT)[14], and 20 lowest-lying singlet excited states were considered at PBE0-D3(BJ)/def2-TZVP level of theory[6-8] with the RIJCOSX approximation[9] (using SMD solvation model[10]) in acetonitrile. The frontier molecular orbitals (FMOs) and natural transition orbitals (NTOs) figures were rendered by Multiwfn[15] and VMD[16] program.

Table S3. Relative Gibbs free energies and Boltzmann distributions of **O4**, **S3**, **S4**, and **S5** in gas and CH_3CN.

Conformation		Rel. Ggas (kcal/mol)	Population (gas)	Rel. Gsoln (kcal/mol)	Population (acetonitrile)
trans-O4	1	0	58.77%	0	68.12%
	2	0.21	41.23%	0.45	31.88%
cis-O4	1	0	52.11%	0	60.40%
	2	0.05	47.89%	0.25	39.60%
trans-S3	1	0.00	99.34%	0.00	97.15%
	2	2.97	0.66%	2.09	2.85%
cis-S3	1	0.00	67.02%	0.00	85.68%
	2	0.42	32.98%	1.06	14.32%
trans-S4	1	0.00	59.99%	0.00	68.85%
	2	0.24	40.01%	0.47	31.15%
cis-S4	1	0.00	53.79%	0.00	67.39%
	2	0.09	46.21%	0.43	32.61%
trans-S5	1	0.00	97.24%	0.00	98.77%
	2	2.11	2.76%	2.60	1.23%
cis-S5	1	0.00	99.16%	0.00	98.40%
	2	2.83	0.84%	2.44	1.60%

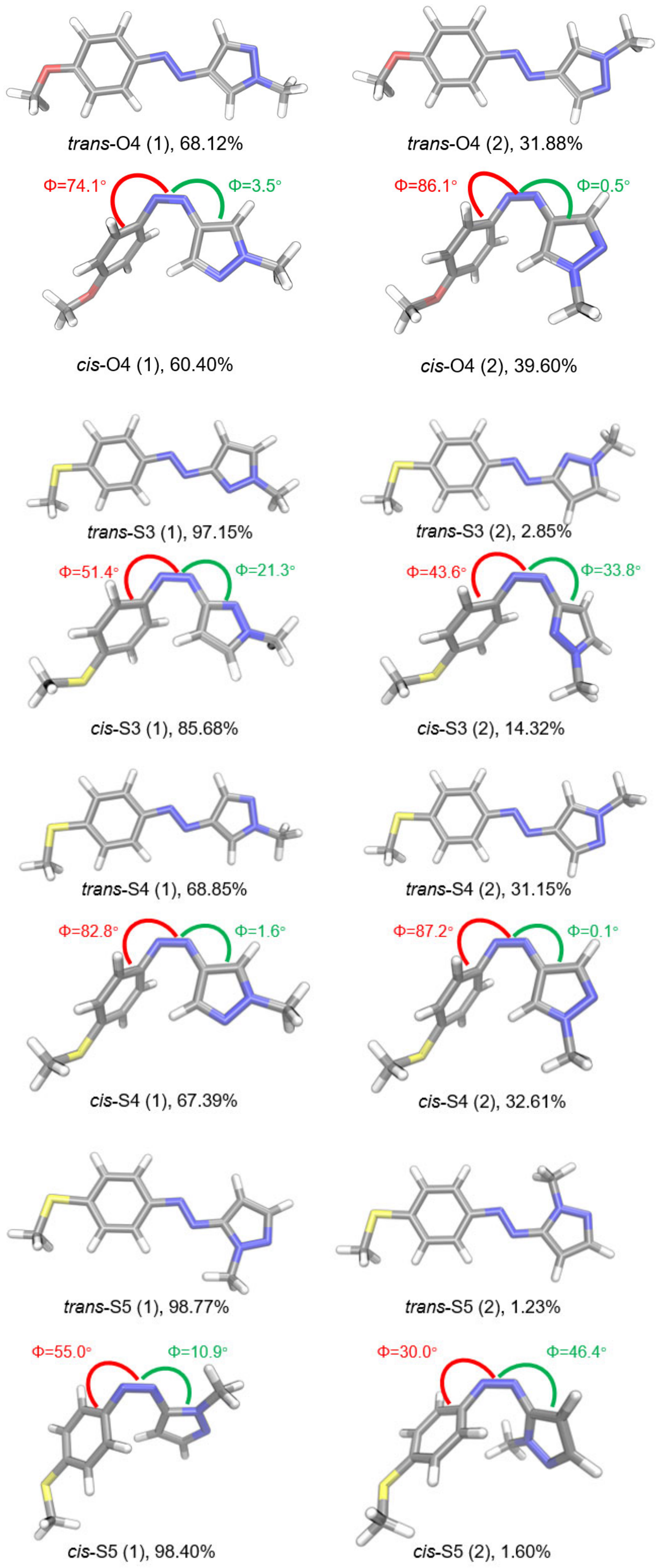

Figure S5. Optimized molecular geometries of **O4**, **S3**, **S4**, and **S5**.

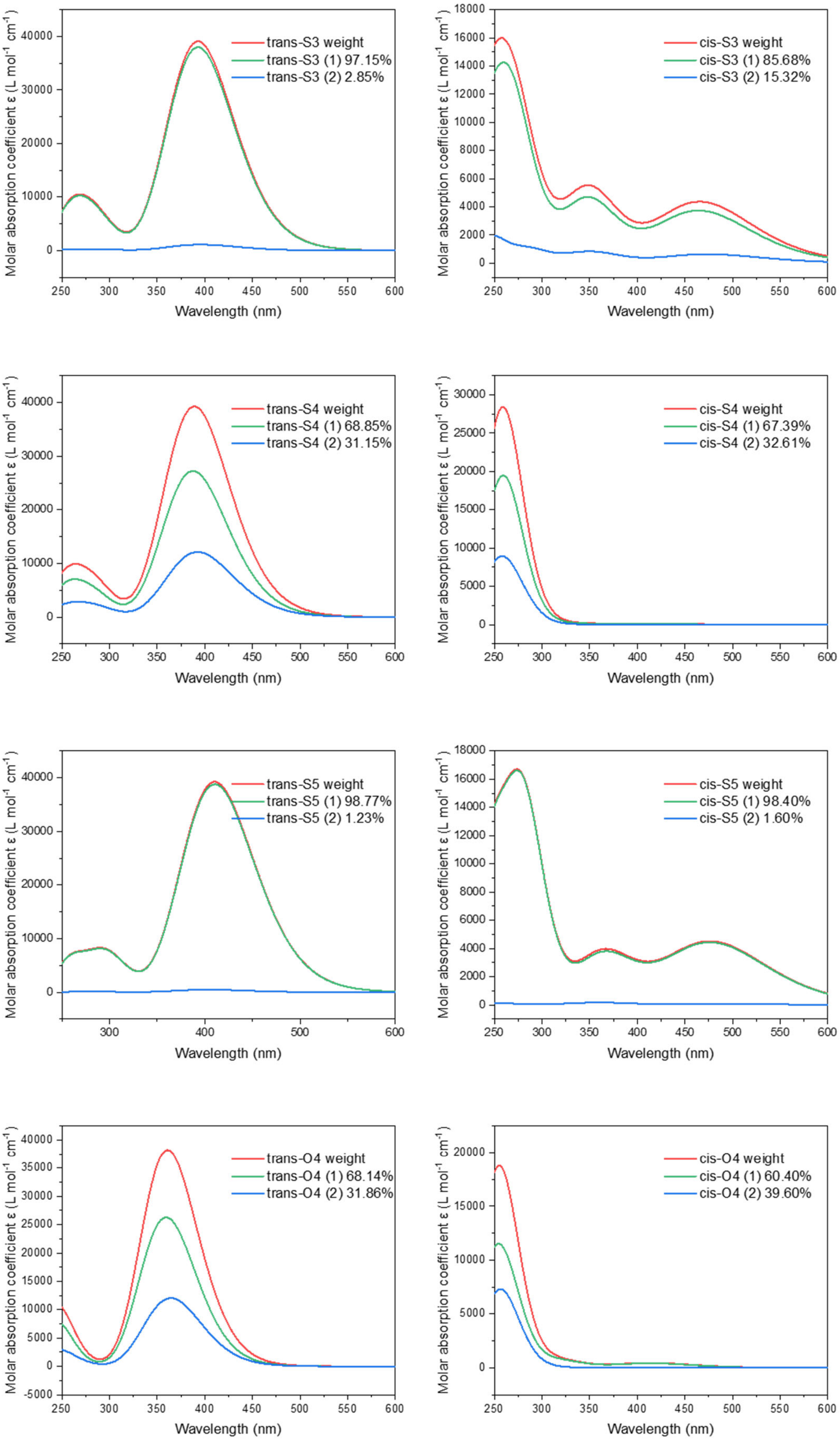

Figure S6. Calculated UV-Vis absorption spectra of **O4**, **S3**, **S4**, and **S5** in CH_3CN.

Table S4. Calculated spectroscopic data of **O4**, **S3**, **S4**, and **S5** in CH_3CN.

Conformation		$S_0 \rightarrow S_1$ Excitation (n-π*)			$S_0 \rightarrow S_2$ Excitation		
		Energy (ev)	λ (nm)	*f*	Energy (ev)	λ (nm)	*f*
trans-O4	1	2.9150	425.33	0.0000	3.4500	359.37	0.9536
	2	2.9090	426.21	0.0001	3.4020	364.45	0.9345
cis-O4	1	2.9900	414.66	0.0170	3.8890	318.81	0.0277
	2	2.9900	414.66	0.0021	3.8560	321.54	0.0021
trans-S3	1	2.8360	437.18	0.0000	3.1560	392.85	0.9669
	2	2.7400	452.50	0.0000	3.1360	395.36	0.9543
cis-S3	1	2.6610	465.93	0.1068	3.5370	350.53	0.1297
	2	2.6130	474.49	0.1090	3.5160	352.63	0.1384
trans-S4	1	2.8690	432.15	0.0000	3.2000	387.45	0.9776
	2	2.8640	432.91	0.0000	3.1590	392.48	0.9630
cis-S4	1	2.9920	414.39	0.0048	3.7030	334.82	0.0057
	2	2.9580	419.15	0.0017	3.6700	337.83	0.0018
trans-S5	1	2.7720	447.27	0.0000	3.0200	410.54	0.9708
	2	2.7320	453.82	0.0000	3.0710	403.73	0.9611
cis-S5	1	2.5890	478.89	0.1096	3.3800	366.82	0.0921
	2	2.5660	483.18	0.1152	3.4260	361.89	0.2605

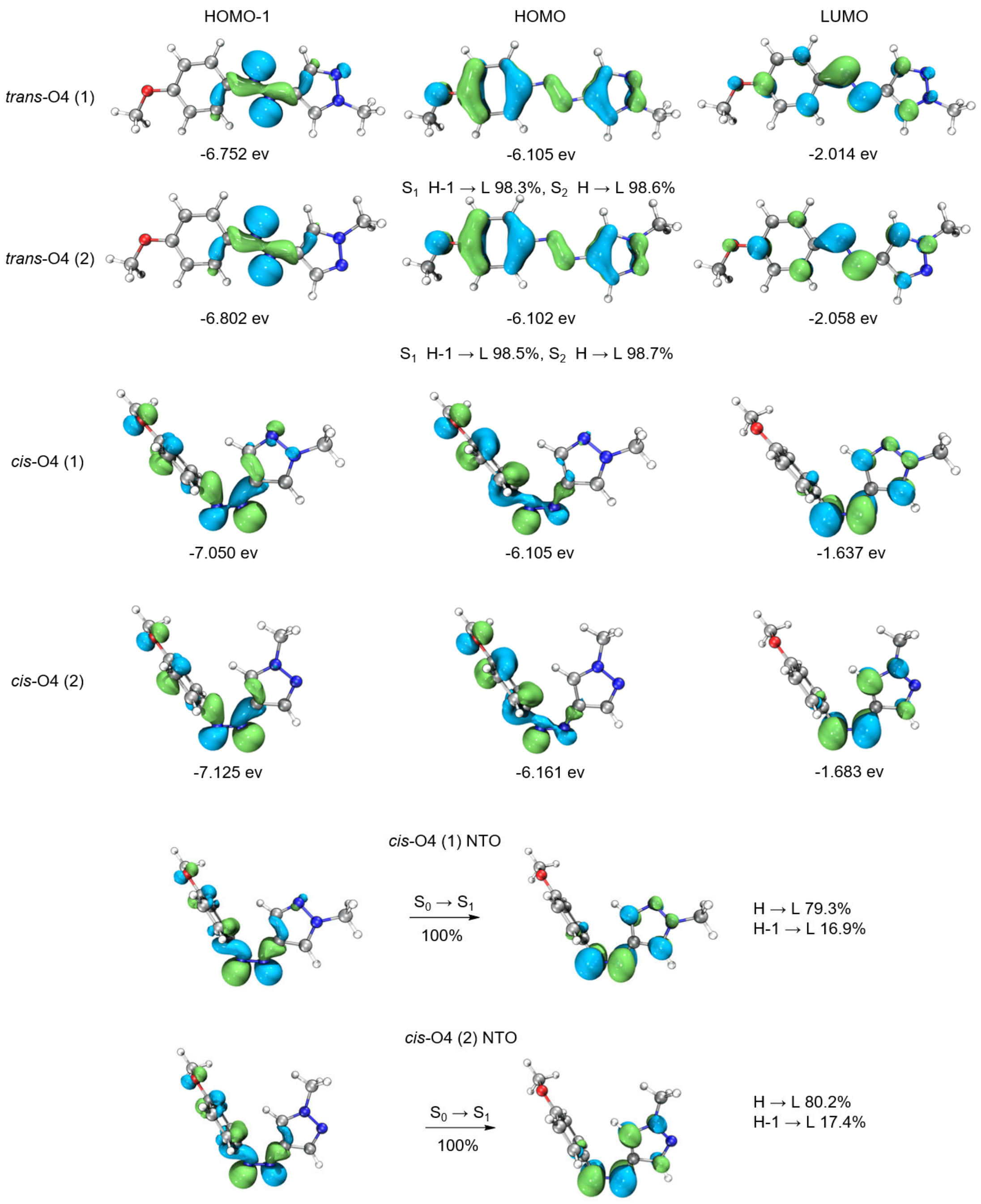

Figure S7. The frontier molecular orbitals (FMOs) and natural transition orbitals (NTOs) of **O4** (isosurface value of 0.05).

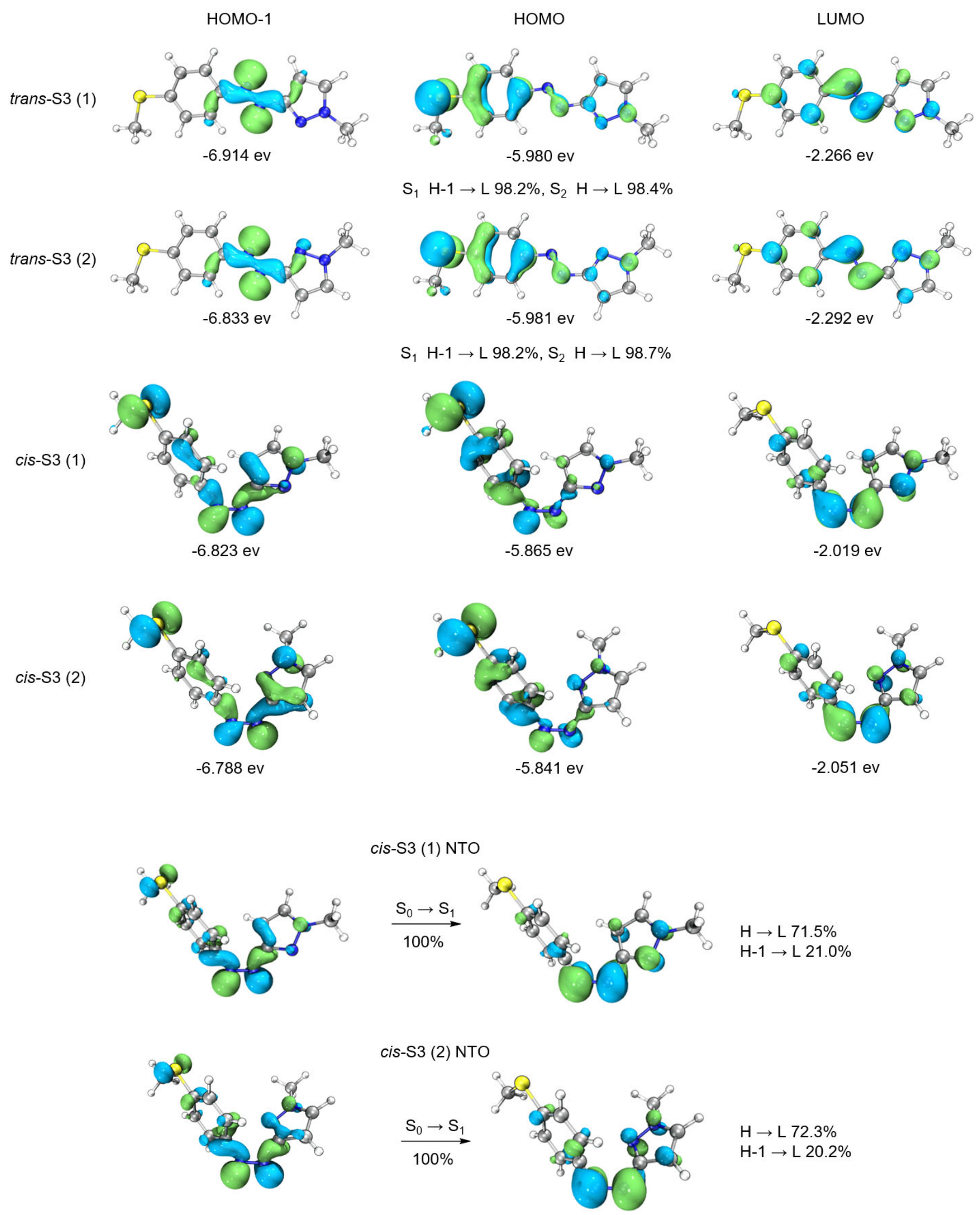

Figure S8. The frontier molecular orbitals (FMOs) and natural transition orbitals (NTOs) of **S3** (isosurface value of 0.05).

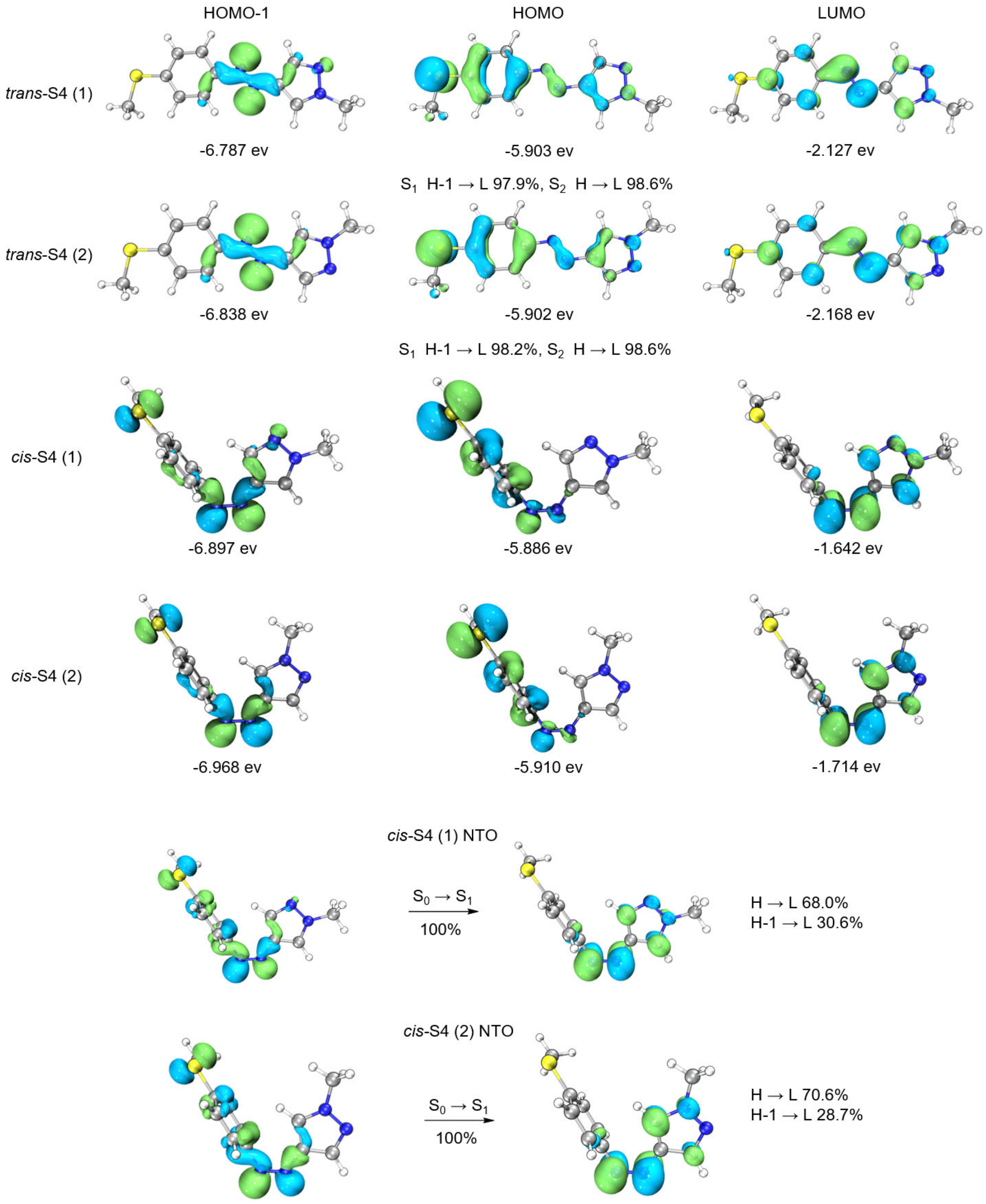

Figure S9. The frontier molecular orbitals (FMOs) and natural transition orbitals (NTOs) of **S4** (isosurface value of 0.05).

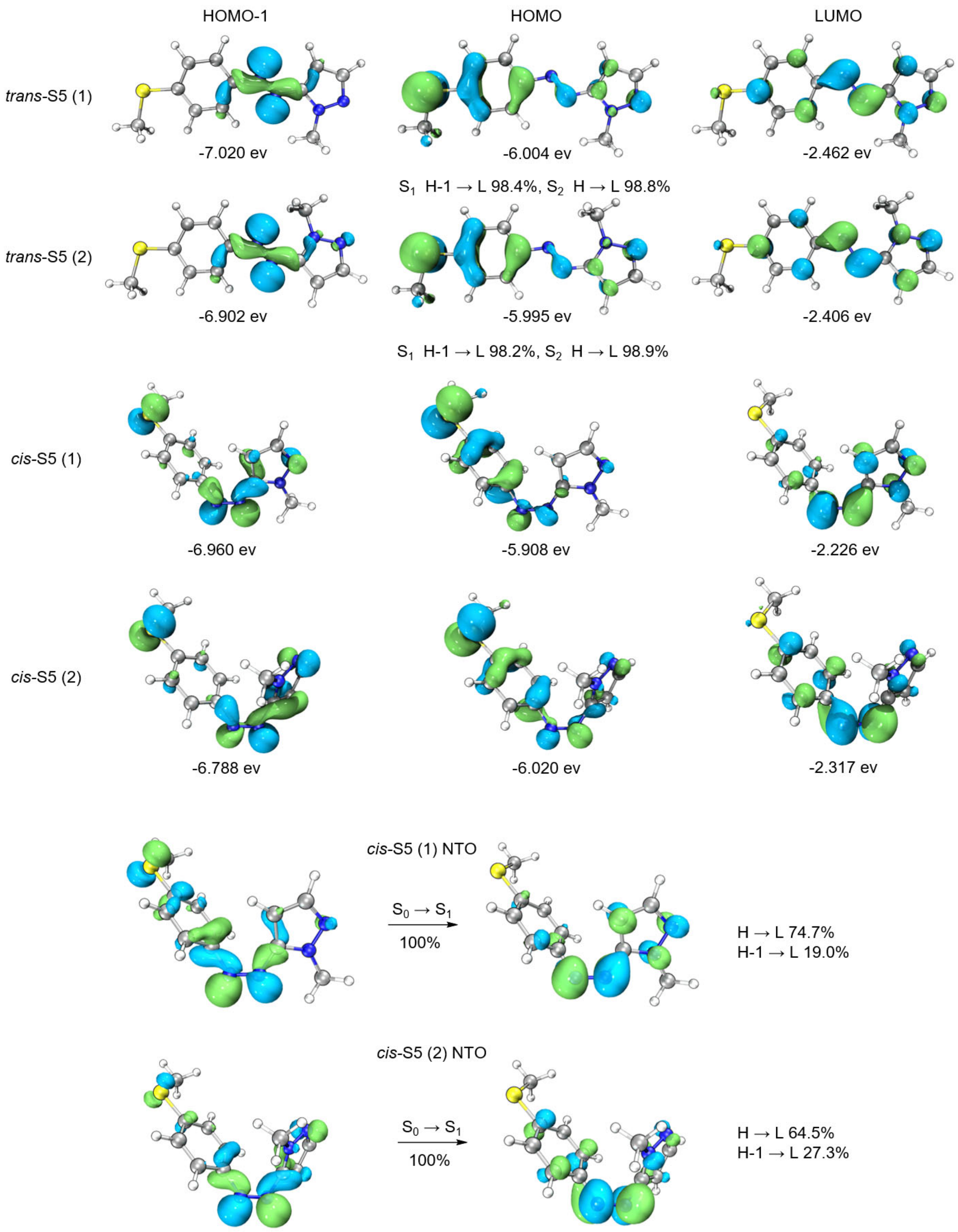

Figure S10. The frontier molecular orbitals (FMOs) and natural transition orbitals (NTOs) of **S5** (isosurface value of 0.05).

4. DSC results for An-S5 and Bn-S5

DSC curves were scanned on Q2000 calorimeter (TA) or DSC 2500 (TA) at a heating rate of 10 °C /min under N_2 atmosphere. The onset temperatures of melting (T_m) and crystallization (T_{cry}) were recorded from the DSC curves.

Table S5. T_m and T_{cry} of **An-S5**.

**An-S5**	*trans*			*cis*	
n	T_m (°C)	T_{cry} (°C)	$\Delta T = T_m - T_{cry}$ (°C)	T_m (°C)	T_{cry} (°C)
2	68.5	-13.0[a]	-	-	-
3	23.7	-0.2[a]	-	-	-
4	27.3	2.2[a]	-	-	-
5	32.7[b]	13.6[a][b]	-	-	-
6	45.7	33.6	12.1	4.6[c]	-23.8[a][c]
7	38.4	26.6	11.8	7.9[b]	-8.9[a][b]
8	49.2	42.6	6.6	11.4[b]	-6.4[a][b]
9	41.8	32.1	9.7	36.6	Not measured
10	48.4	22.4	26.0	28.7	Not measured
11	55.9	36.1	19.8	49.3	Not measured
12	56.6	27.1	29.5	47.0	Not measured

[a] Cold-crystallization temperature. [b] Crystal transformation occurred during the heating cycle, and the T_m and T_{cry} of the high-temperature crystal form were recorded. [c] The T_m of 75% *cis*-isomer content.

Table S6. T_m and T_{cry} of **Bn-S5**.

**Bn-S5**	*trans*			*cis*	
n	T_m (°C)	T_{cry} (°C)	$\Delta T = T_m - T_{cry}$ (°C)	T_m (°C)	T_{cry} (°C)
3	-	-	-	-	-
4	-	-	-	-	-
5	-	-	-	-	-
6	11.7	-7.3	-	-	-
7	40.0	33.7	6.3	-1.0[c]	-20.0[a][c]
8	22.4	-12.0, -49.8[a]	-	-	-
9	47.6	39.0	8.6	16.7[b]	-10.7[a][b]
10	37.9	1.1	36.8	14.2[b]	4.2[a][b]
11	39.8	11.3	28.5	30.0	Not measured

[a] Cold-crystallization temperature. [b] Crystal transformation occurred during the heating cycle, and the T_m and T_{cry} of the high-temperature crystal form were recorded. [c] The T_m of 75% *cis*-isomer content.

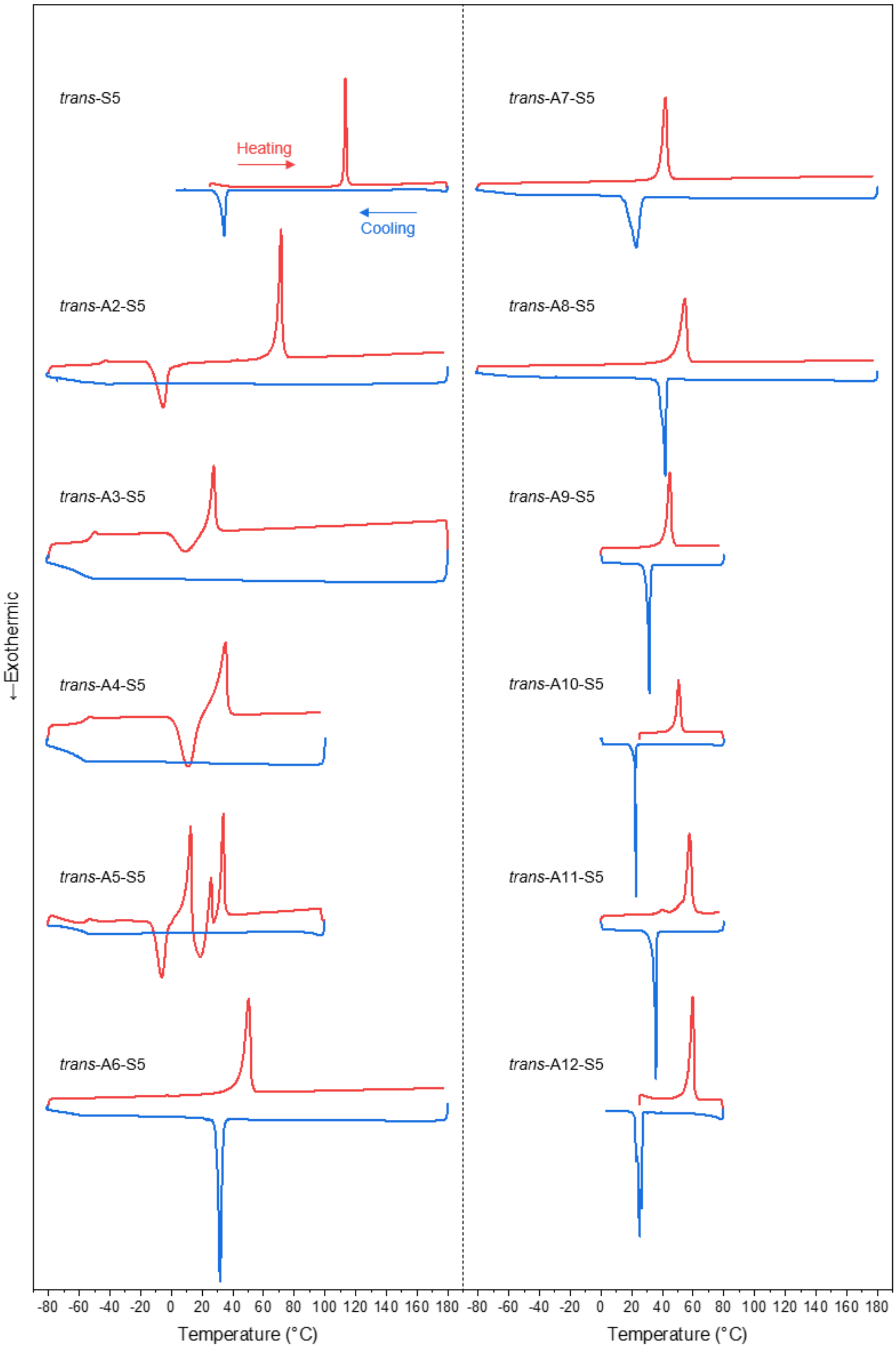

Figure S11. DSC curves of *trans*-**An-S5** measured at 10 °C/min.

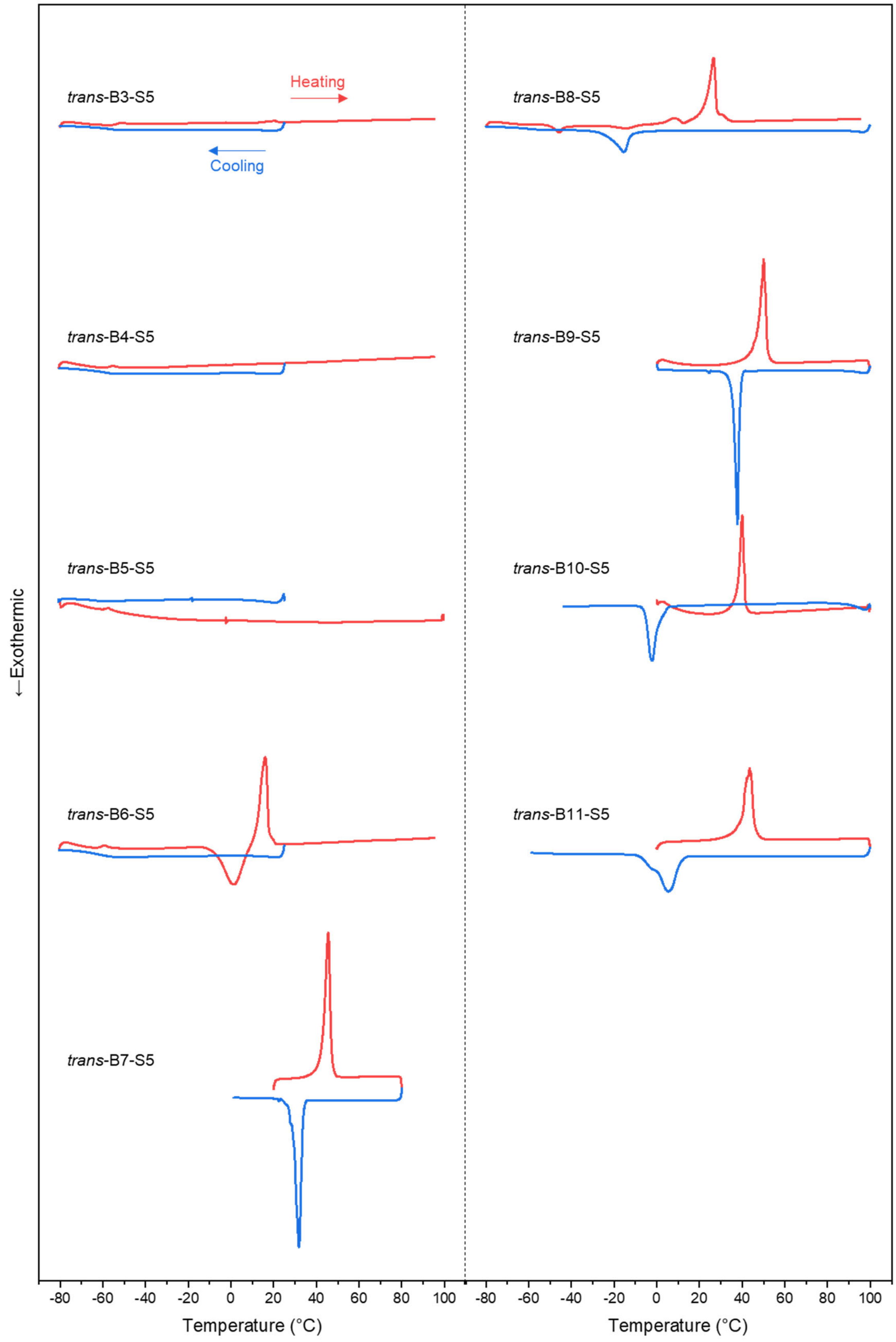

Figure S12. DSC curves of *trans*-**Bn-S5** measured at 10 °C/min.

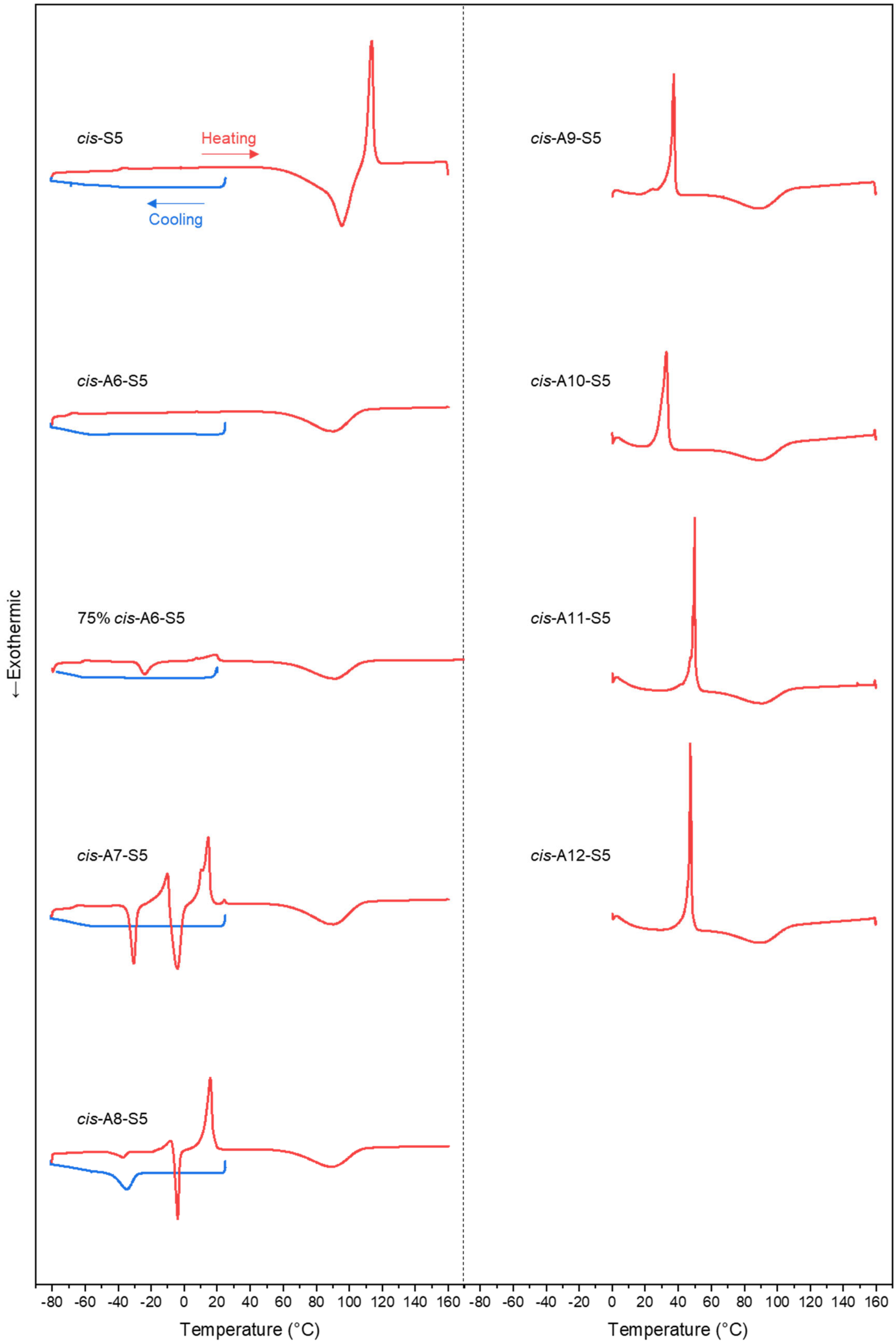

Figure S13. DSC curves of *cis*-**An-S5** measured at 10 °C/min.

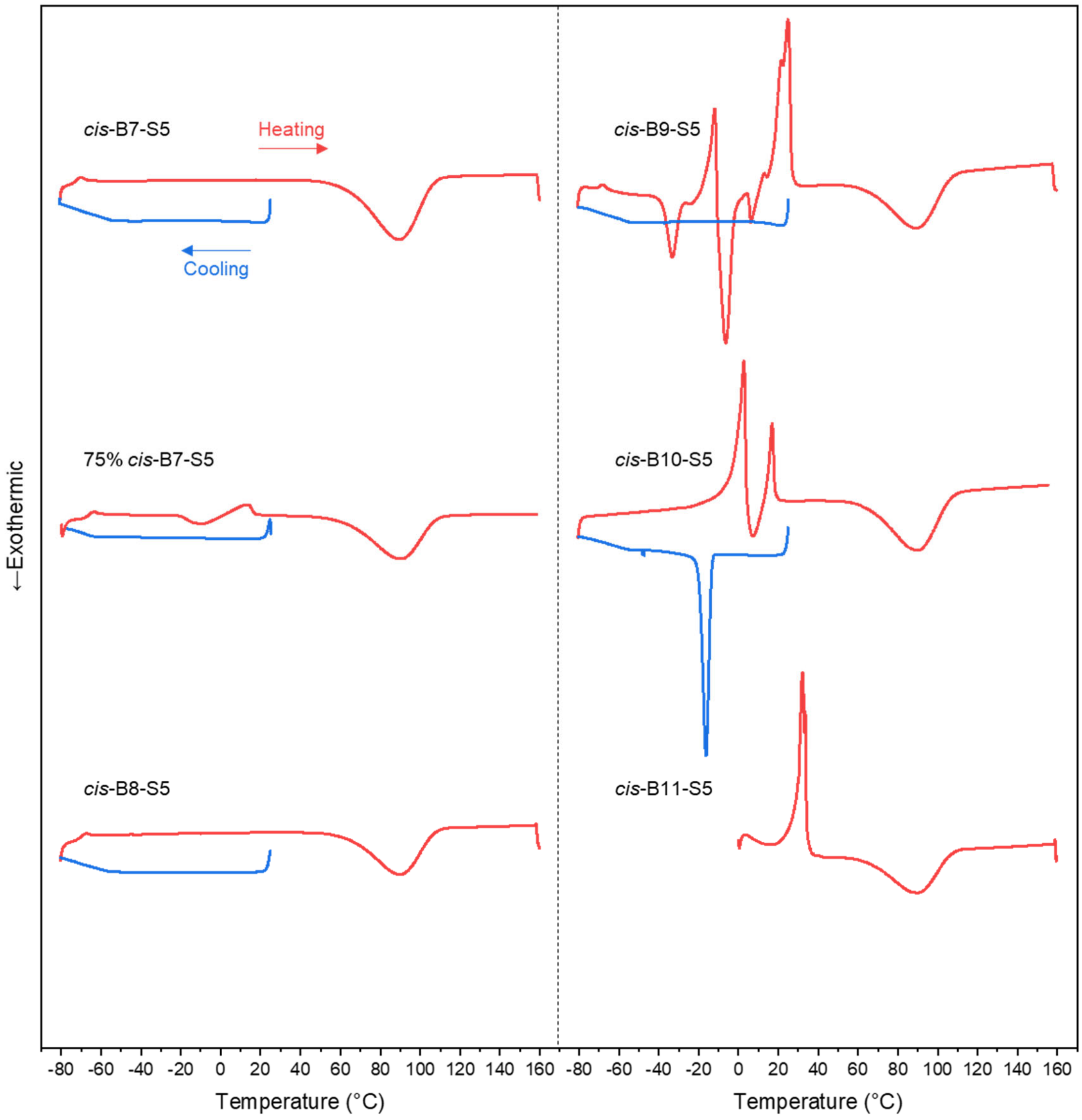

Figure S14. DSC curves of *cis*-**Bn-S5** measured at 10 °C/min.

Table S7. ΔH_{iso} of **S5** obtained by calculation and experiment.

	Conformation	Enthalpy (Hartree)	Population (gas)	Avg. Enthalpy (Hartree)	Cal. ΔH_{iso} (kJ/mol)	Exp. ΔH_{iso} (kJ/mol)
**S5**	*trans*-S5 (1)	-1043.120575	97.24%	-1043.120516	49	46
	trans-S5 (2)	-1043.118454	2.76%			
	cis-S5 (1)	-1043.101814	99.16%	-1043.101783		
	cis-S5 (2)	-1043.098182	0.84%			

5. Thermal isomerization kinetics and half-life

Thermal *cis–trans* kinetics was studied using Shimadzu UV-2700 spectrophotometer equipped with a programmable temperature controller (DCY-1006, Shanghai Sunny Hengping Scientific Instrument Ltd). The absorbance at λ_{max} = 385 nm of a *cis*-rich acetonitrile solution was recorded every minute.

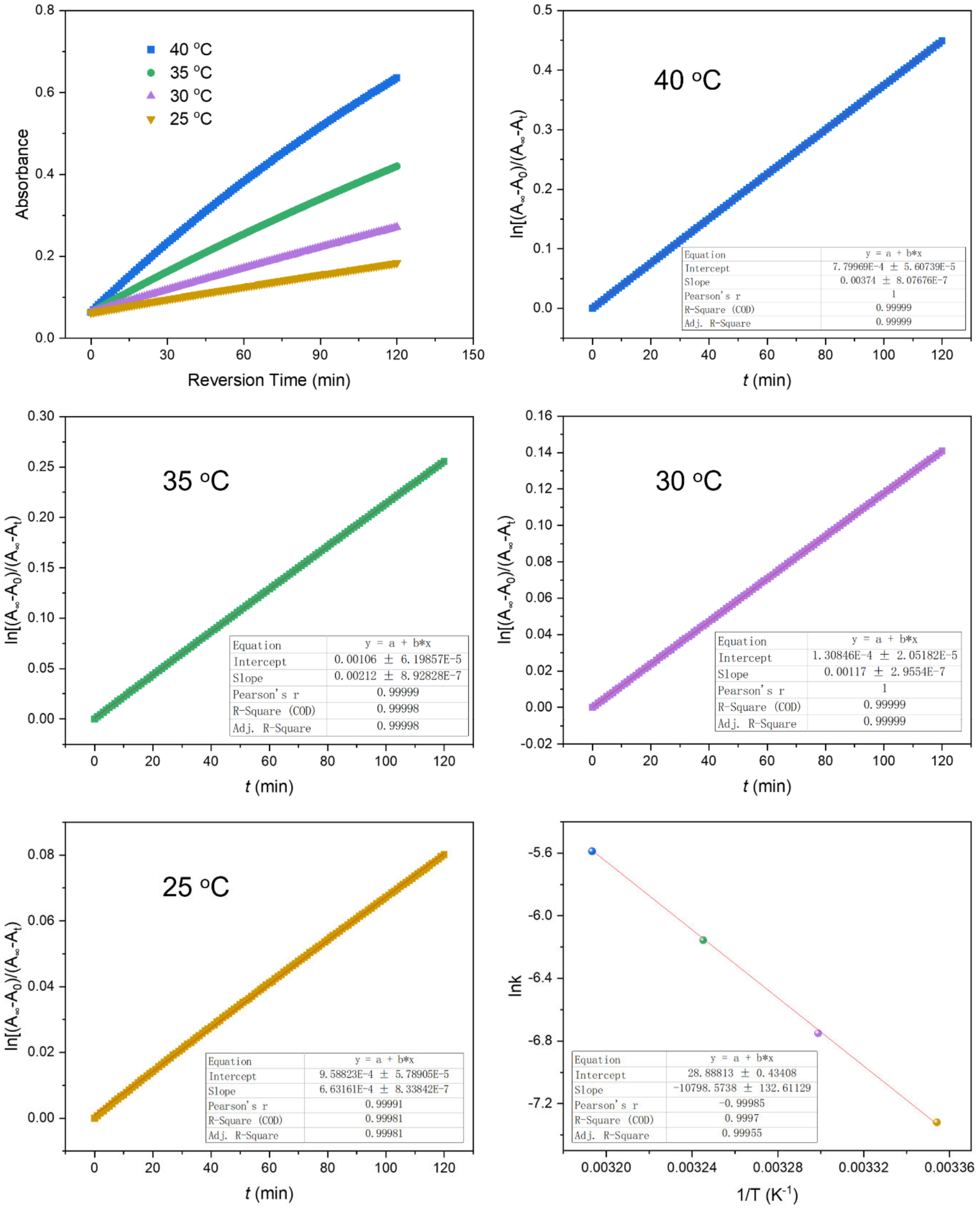

Figure S15. Thermal *cis–trans* recovery of *cis*-**B7-S5** in acetonitrile with time at different temperatures followed by the absorbance increase at λ_{max} = 385 nm, and the linear fit between ln*k* and 1/*T* according to the Arrhenius equation.

Thermal *cis–trans* kinetics in condensed states was studied as follows. The temperature of *cis*-rich liquid was controlled by a semiconductor cooling and heating platform (RTL100, Huozi Instrument Technology Ltd). A small sample was taken from the *cis*-rich liquid every 5 or 10 minutes, dissolved in acetonitrile, and recorded the UV-Vis absorption spectrum.

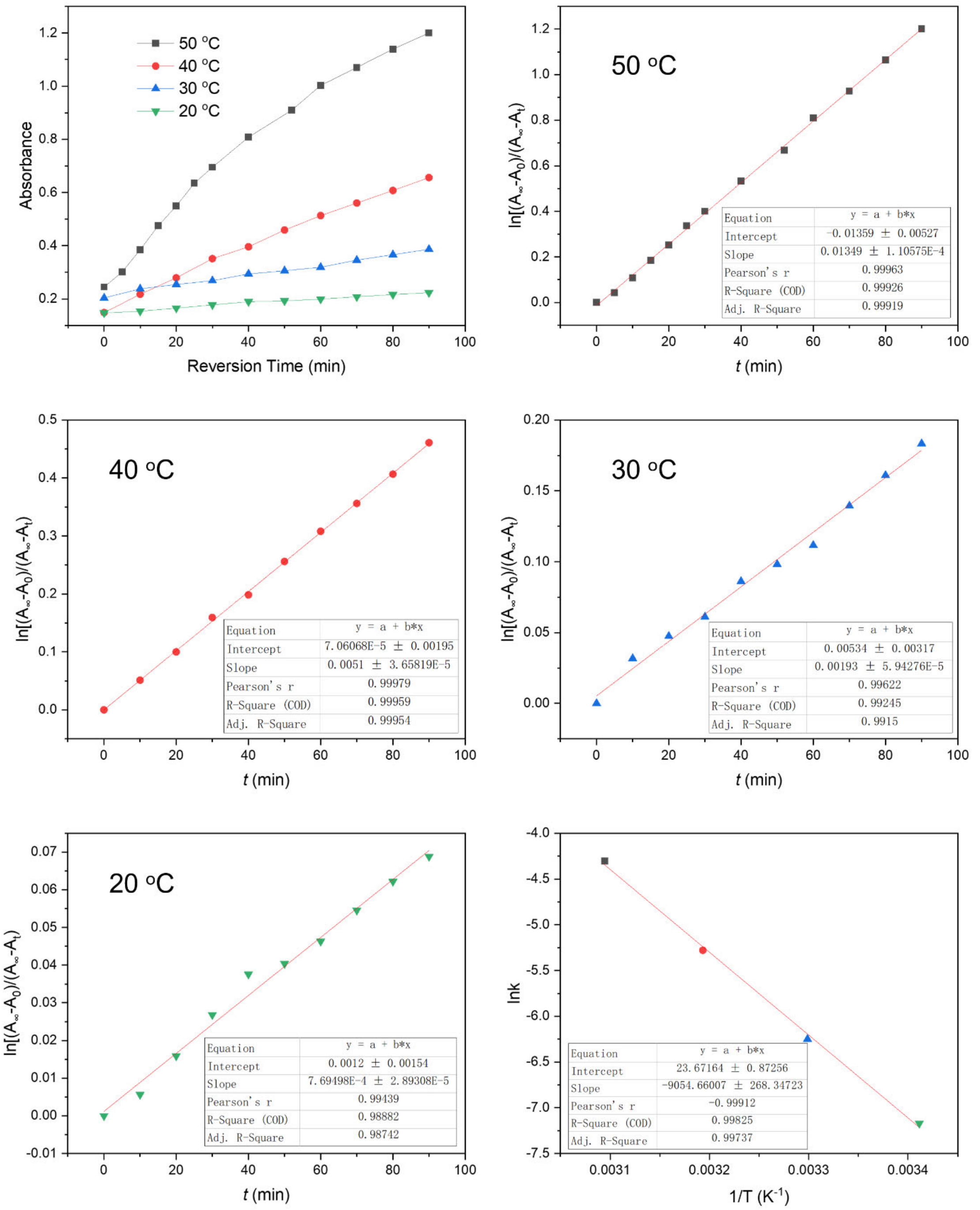

Figure S16. Thermal *cis–trans* recovery of *cis*-**B7-S5** in condensed states with time at different temperatures followed by the absorbance increase at λ_{max} = 385 nm, and the linear fit between ln*k* and 1/*T* according to the Arrhenius equation.

Table S8. Thermal isomerization kinetics results of *cis*-**B7-S5** in acetonitrile.

	Experimental					Calculated
	40 °C	35°C	30 °C	25 °C		0 °C
k (min^{-1})	3.74×10^{-3}	2.12×10^{-3}	1.17×10^{-3}	6.63×10^{-4}	k (min^{-1})	2.14×10^{-5}
$t_{1/2}$ (min)	185	327	592	1045	$t_{1/2}$ (d)	22.4

Table S9. Thermal isomerization kinetics results of *cis*-**B7-S5** in condensed states.

	Experimental					Calculated
	50 °C	40°C	30 °C	20 °C		0 °C
k (min^{-1})	1.35×10^{-2}	5.10×10^{-3}	1.93×10^{-3}	7.69×10^{-4}	k (min^{-1})	7.64×10^{-5}
$t_{1/2}$ (min)	51	136	359	901	$t_{1/2}$ (d)	6.3

6. Quantum yield and solar energy conversion efficiency

The photon flux of 395 nm LED light was 1.11×10^{-4} mol s^{-1}, which was determined by chemical actinometry using potassium ferrioxalate as a standard actinometer[3, 17]. Photoisomerization quantum yield ($\Phi_{trans\rightarrow cis}$) was measured by using an established method from literature[3].

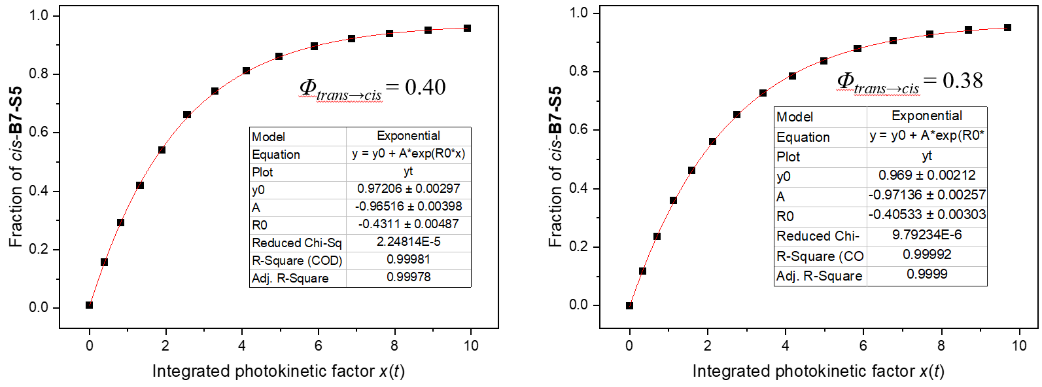

Figure S17. Exponential fitting of the fractions of *cis*-**B7-S5** against the integrated photokinetic factors $x(t)$.

Under AM 1.5 solar irradiation spectrum, the solar energy conversion efficiency (η) can be estimated with the following equation[18]:

$$\eta = \frac{\int_0^{\lambda_{onset}} \frac{E_{\mathrm{AM\ 1.5}}(\lambda)\Phi_{trans\rightarrow cis}\Delta H_{\mathrm{iso}}}{h\upsilon N_A} d\lambda}{\int E_{\mathrm{AM\ 1.5}}(\lambda) d\lambda} 100\%$$

Table S10. Solar energy conversion efficiency of **B7-S5**.

Molecule	Exp. ΔH_{iso} (kJ/mol)	$\Phi_{trans\rightarrow cis}$	λ_{onset} (nm) (> 100)	η
**B7-S5**	46	0.39±0.01	515	1.3%

7. Preparation of rechargeable glass

The diethoxydimethylsilane modified chain-like silica solution was prepared according to the reported method.[19] 42 mg of chain-like silica was added to 6 ml of a mixed solution of ethanol and isopropanol (1:1, v/v) at room temperature, and sonicated for 15 min. Then, 147 mg diethoxydimethylsilane and 25 mg HCl were slowly added to the mixture, and the mixture was stirred for 24 hours to obtain diethoxydimethylsilane modified chain-like silica. Finally, 180 mg **B7-S5** was added to the solution, stirred for 15 min to give orange-yellow **silica-B7-S5** mixture for drop-casting.

Rechargeable glass was made by drop-cast method. The glass (22 mm × 22 mm) was clean in deionized water, acetone and isopropanol sequentially by ultrasonication and then treated with oxygen plasma for 10 min, after which the glass was placed on a 60 °C hot plate. Then 1 mL of **silica-B7-S5** solution was dropped on a pre-heated glass surface to form a **silica-B7-S5** coating with a thickness of ~ 400 μm on glass.

The neat **B7-S5** coated glass was prepared by drop-coating a 30 mg mL^{-1} **B7-S5** mixed solution of ethanol and isopropanol on the glass.

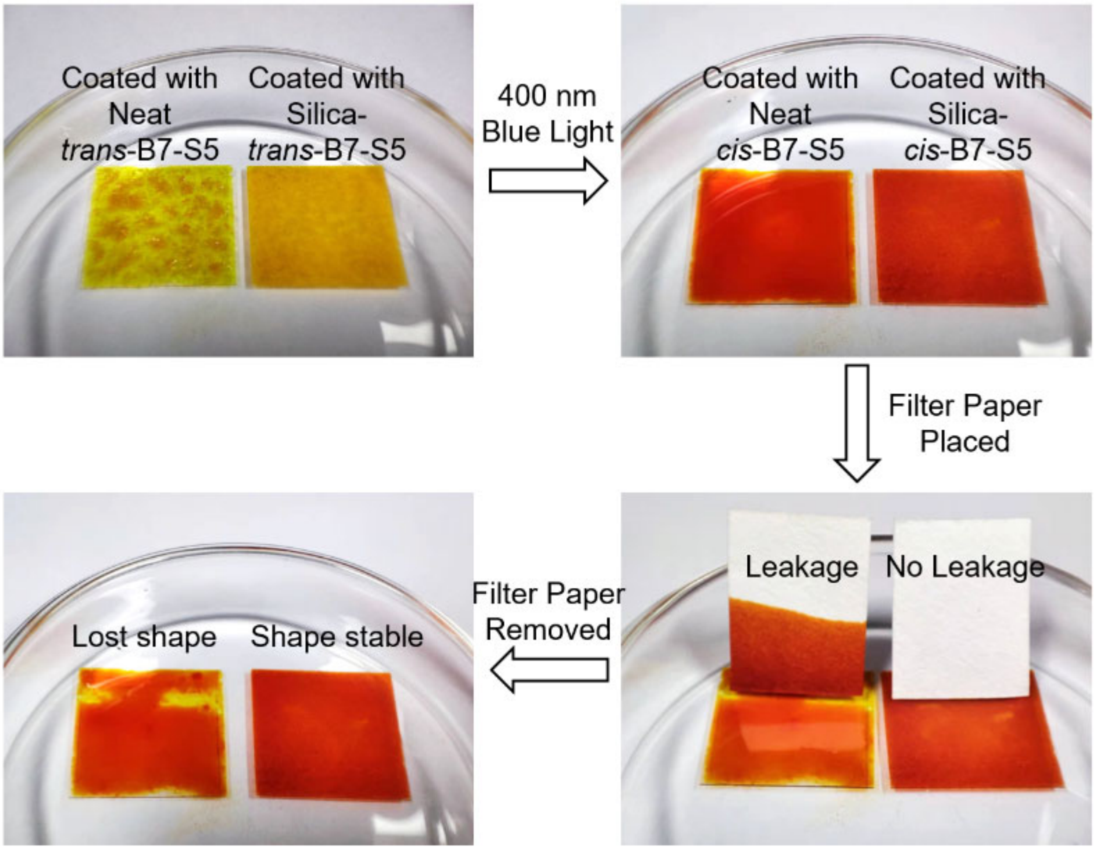

Figure S18. Leakage tests of neat **B7-S5** and **silica-B7-S5**.

8. Infrared image

A high-resolution infrared thermal imaging camera was used to track the temperature changes of the window when exposed to 400 nm (40 mW cm^{-2}) and 532 nm light (110 mW cm^{-2}).

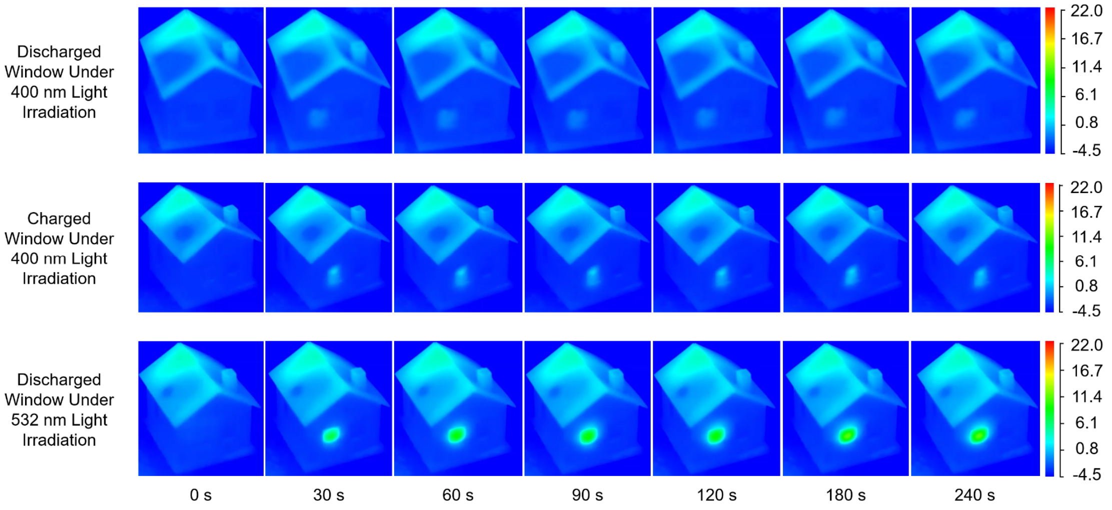

Figure S19. Time-evolved infrared images of the windows under 400 nm (40 mW cm^{-2}) and 532 nm light (110 mW cm^{-2}) irradiation at -1 °C.

9. Single crystal X-ray diffraction

Crystallography data were collected on a Bruker D8 VENTURE single crystal x-ray diffractometer. The crystal structures were solved and refined using SHELX programs[20-22] called from ShelXle[23] and Olex2[24] graphical user interfaces.

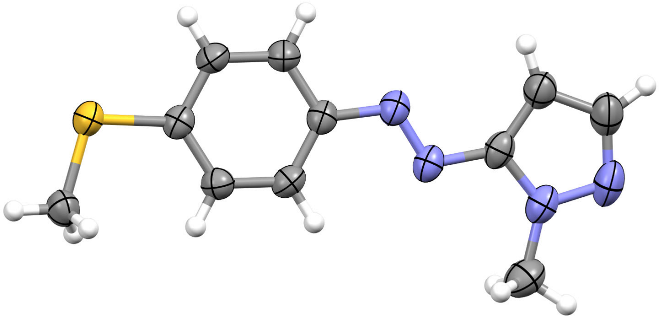

Figure S20. Crystal structure of **S5**. Gray: Carbon, blue: Nitrogen, yellow: Sulfur, white: Hydrogen.

Table S11. Crystal data and structure refinement of **S5** (CCDC 2112835).

Empirical formula	$C_{11}H_{12}N_4S$	
Formula weight	232.31	
Temperature	298(2) K	
Wavelength	1.54178 Å	
Crystal system, space group	Monoclinic, $P\ 2_1/c$	
Unit cell dimensions	a = 10.1876(19) Å	α = 90°.
	b = 9.2946(14) Å	β = 104.262(19)°.
	c = 12.4426(18) Å	γ = 90°.
Volume	1141.9(3) $Å^3$	
Z, Calculated density	4, 1.351 g/cm^3	
Absorption coefficient	2.333 mm^{-1}	
$F(000)$	488	
Crystal size	0.160 x 0.140 x 0.120 mm	
Theta range for data collection	4.478 to 68.297°	
Limiting indices	-11⩽h⩽12, -11⩽k⩽11, -14⩽l⩽14	
Reflections collected / unique	13012 / 2087 (R_{int} = 0.0400)	
Completeness to theta = 67.679	99.8 %	
Refinement method	Full-matrix least-squares on F^2	
Data / restraints / parameters	2087 / 0 / 147	
Goodness-of-fit on F^2	1.044	
Final R indices [I>2sigma(I)]	R_1 = 0.0327, wR_2 = 0.0908	
R indices (all data)	R_1 = 0.0389, wR_2 = 0.0955	
Largest diff. peak and hole	0.218 and -0.206 e.$Å^{-3}$	

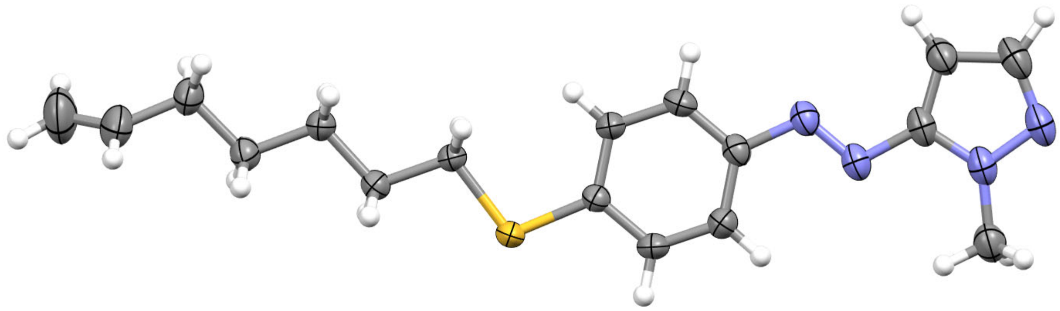

Figure S21. Crystal structure of **B7-S5**. Gray: Carbon, blue: Nitrogen, yellow: Sulfur, white: Hydrogen.

Table S12. Crystal data and structure refinement of **B7-S5** (CCDC 2112834).

Empirical formula	$C_{17}H_{22}N_4S$
Formula weight	314.44
Temperature	173(2) K
Wavelength	1.54178 Å
Crystal system, space group	Triclinic, P-1
Unit cell dimensions	a = 5.6168(2) Å α = 87.1020(10)°.
	b = 8.1750(2) Å β = 88.922(2)°.
	c = 39.0525(10) Å γ = 73.340(2)°.
Volume	1715.70(9) $Å^3$
Z, Calculated density	4, 1.217 g/cm^3
Absorption coefficient	1.680 mm^{-1}
F(000)	672
Crystal size	0.140 x 0.120 x 0.100 mm
Theta range for data collection	3.399 to 68.250°
Limiting indices	$-6\leq h\leq 6$, $-9\leq k\leq 9$, $-46\leq l\leq 46$
Reflections collected / unique	34076 / 6270 (R_{int} = 0.0660)
Completeness to theta = 67.679	99.8 %
Refinement method	Full-matrix least-squares on F^2
Data / restraints / parameters	6270 / 0 / 399
Goodness-of-fit on F^2	1.038
Final R indices [I>2sigma(I)]	R_1 = 0.0428, wR_2 = 0.1043
R indices (all data)	R_1 = 0.0649, wR_2 = 0.1155
Largest diff. peak and hole	0.578 and -0.295 e.$Å^{-3}$

10. NMR spectra

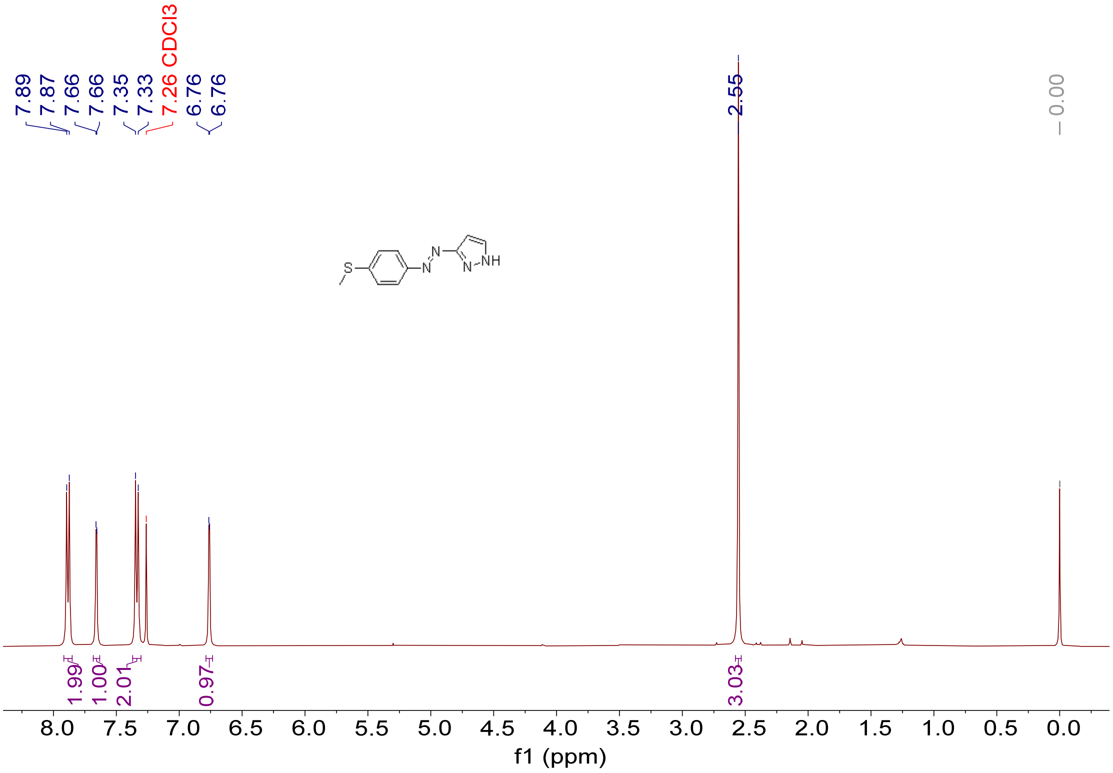

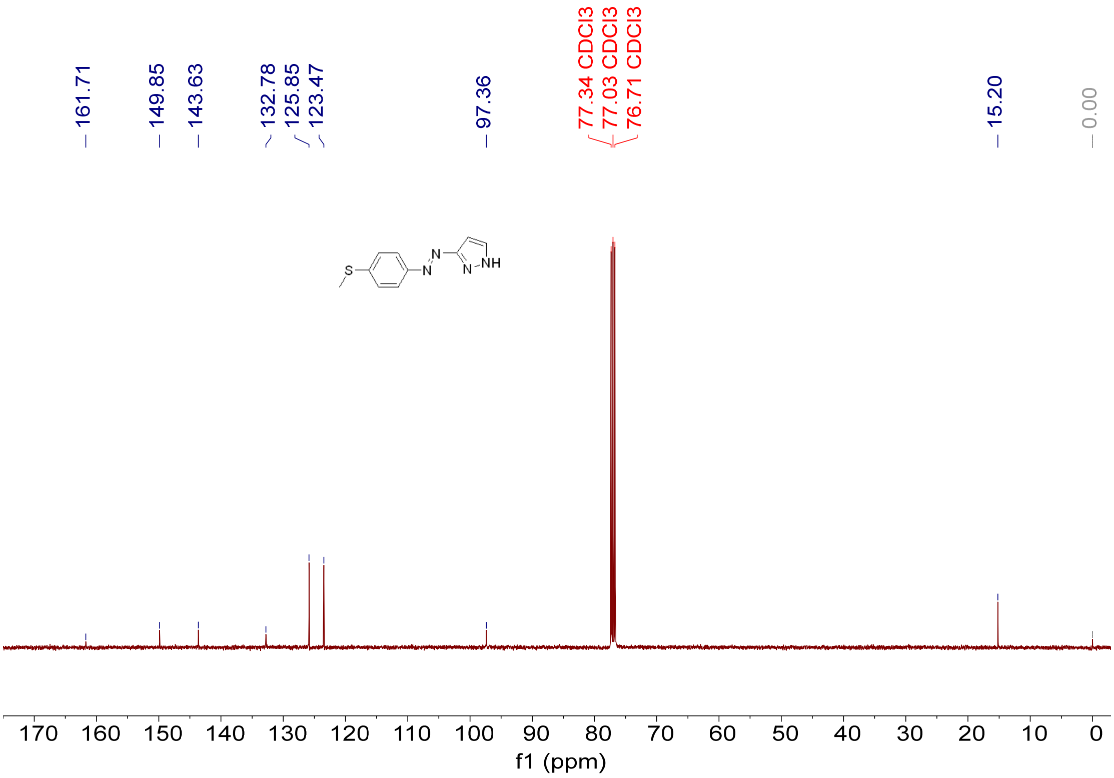

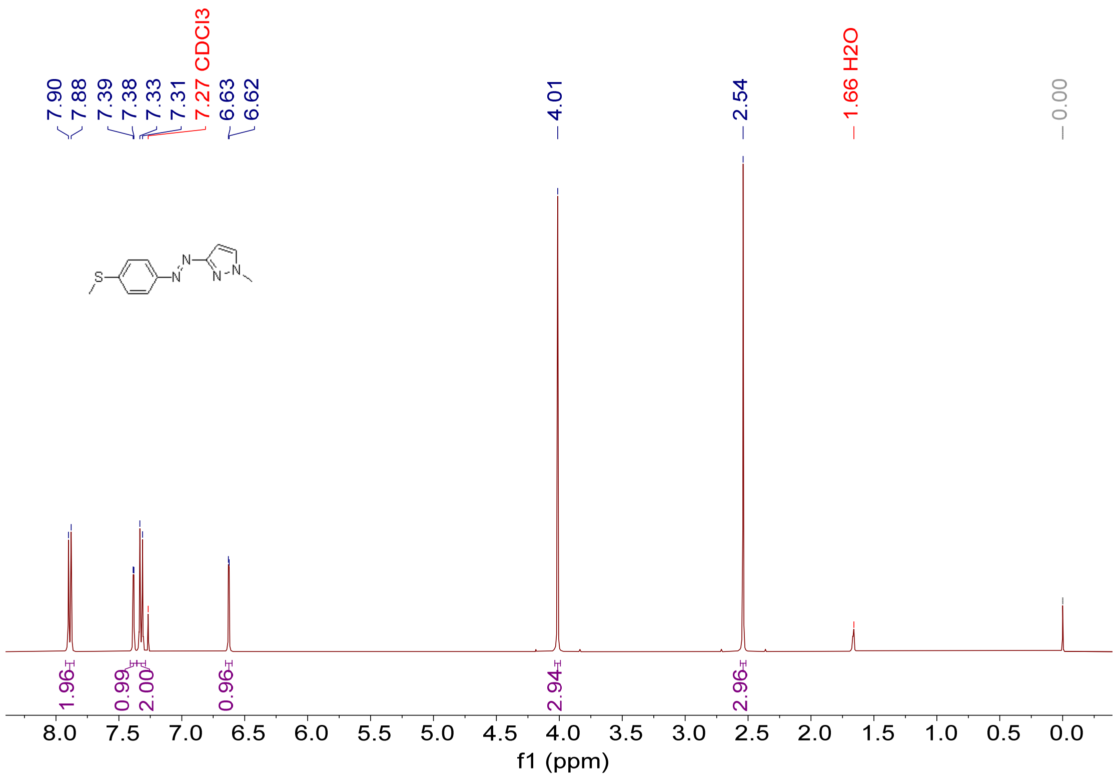
7.90
7.88
7.39
7.38
7.33
7.31
7.27 CDCl3
6.63
6.62
4.01
2.54
1.66 H2O
0.00
1.96
0.99
2.00
0.96
2.94
2.96
S
N
N
N
N
f1 (ppm)

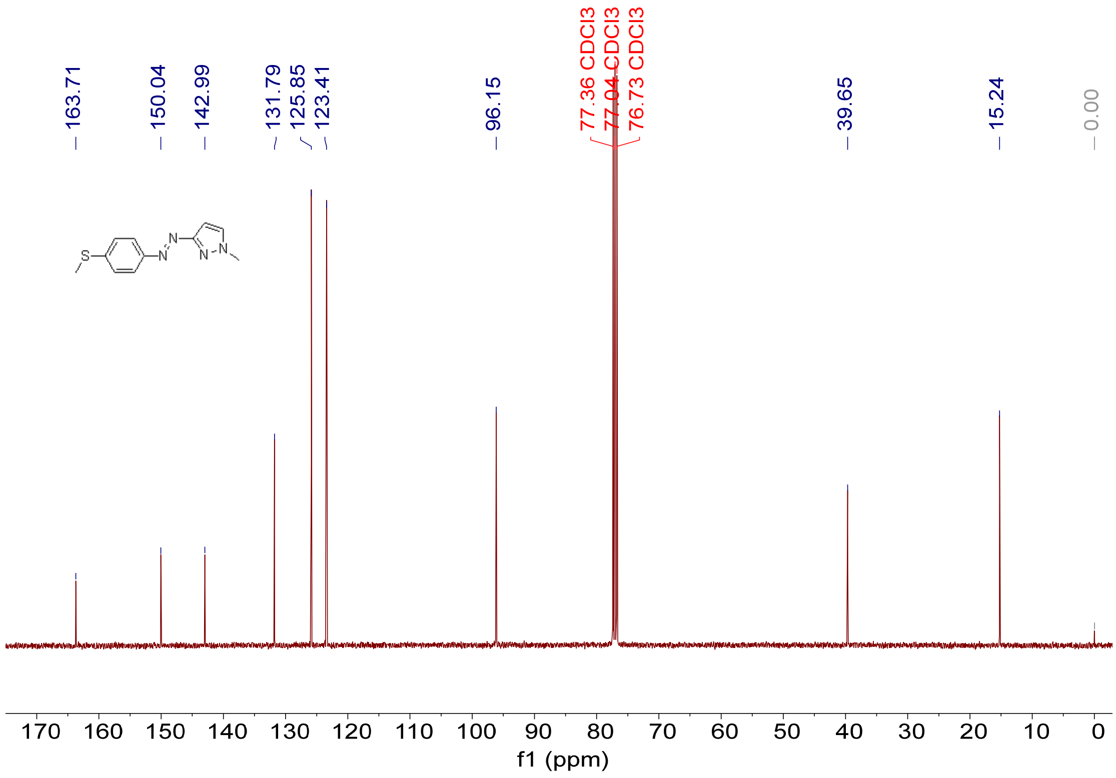
163.71
150.04
142.99
131.79
125.85
123.41
96.15
77.36 CDCl3
77.04 CDCl3
76.73 CDCl3
39.65
15.24
0.00
S
N
N
N
N
f1 (ppm)

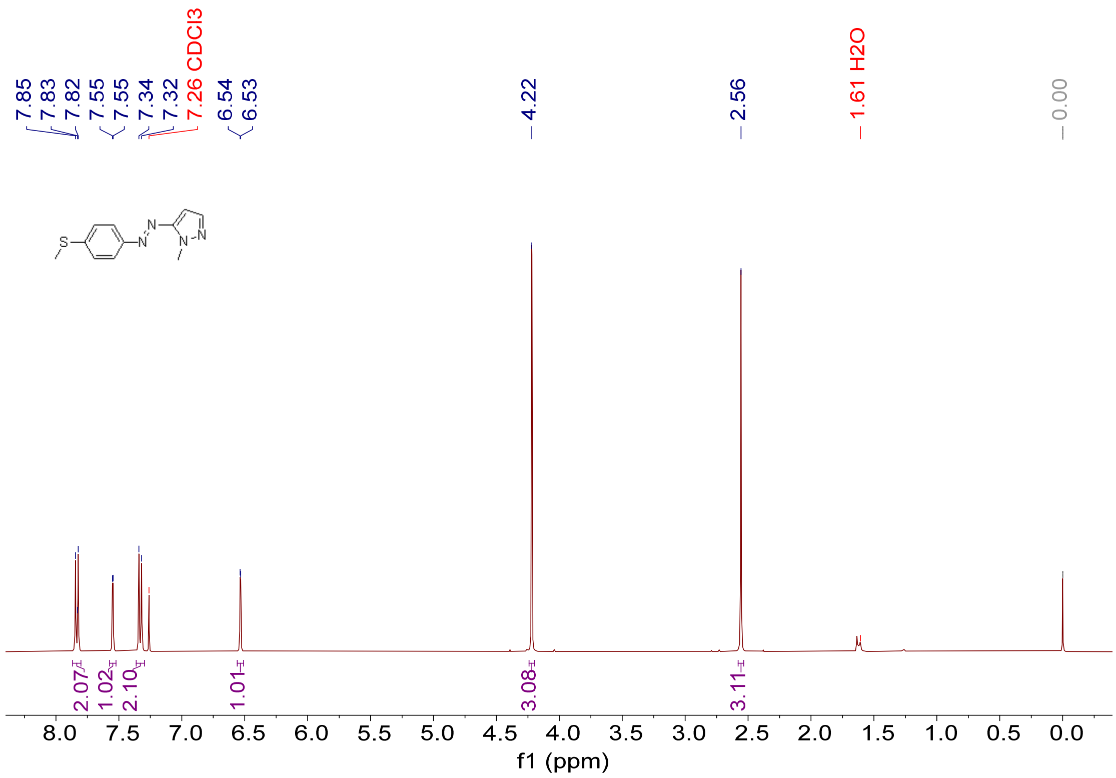
7.85
7.83
7.82
7.55
7.55
7.34
7.32
7.26 CDCl3
6.54
6.53
4.22
2.56
1.61 H2O
0.00
2.07
1.02
2.10
1.01
3.08
3.11
8.0 7.5 7.0 6.5 6.0 5.5 5.0 4.5 4.0 3.5 3.0 2.5 2.0 1.5 1.0 0.5 0.0
f1 (ppm)

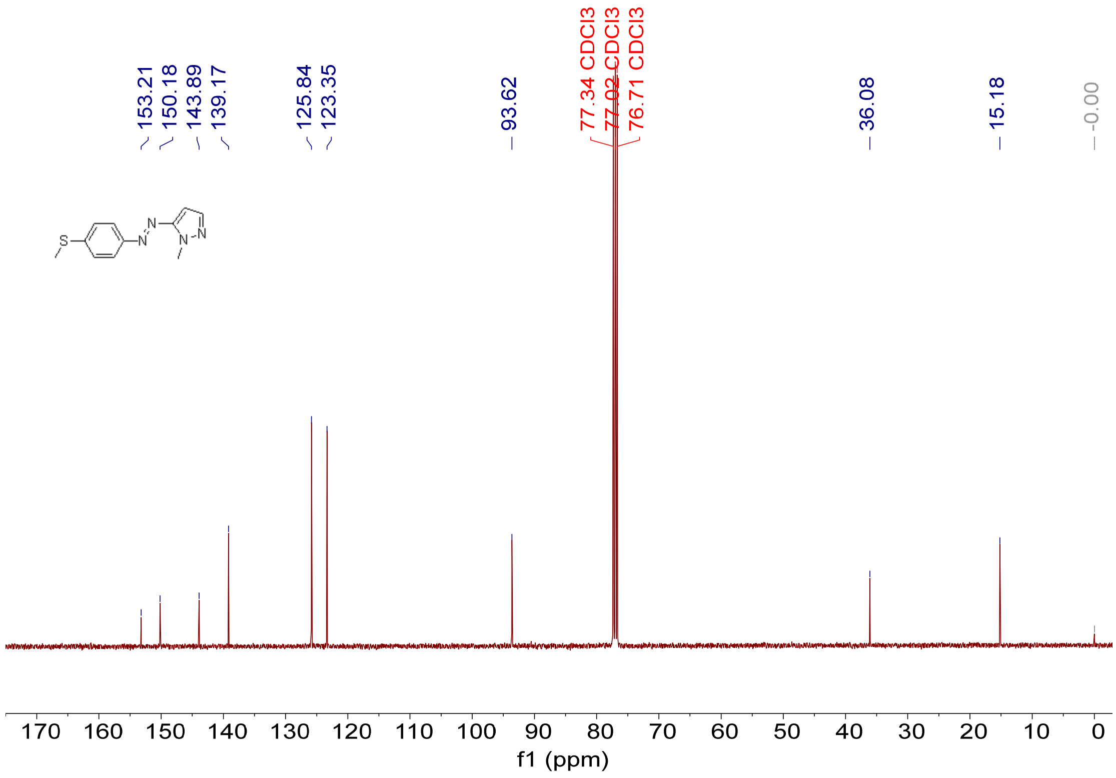
153.21
150.18
143.89
139.17
125.84
123.35
93.62
77.34 CDCl3
77.02 CDCl3
76.71 CDCl3
36.08
15.18
-0.00
170 160 150 140 130 120 110 100 90 80 70 60 50 40 30 20 10 0
f1 (ppm)

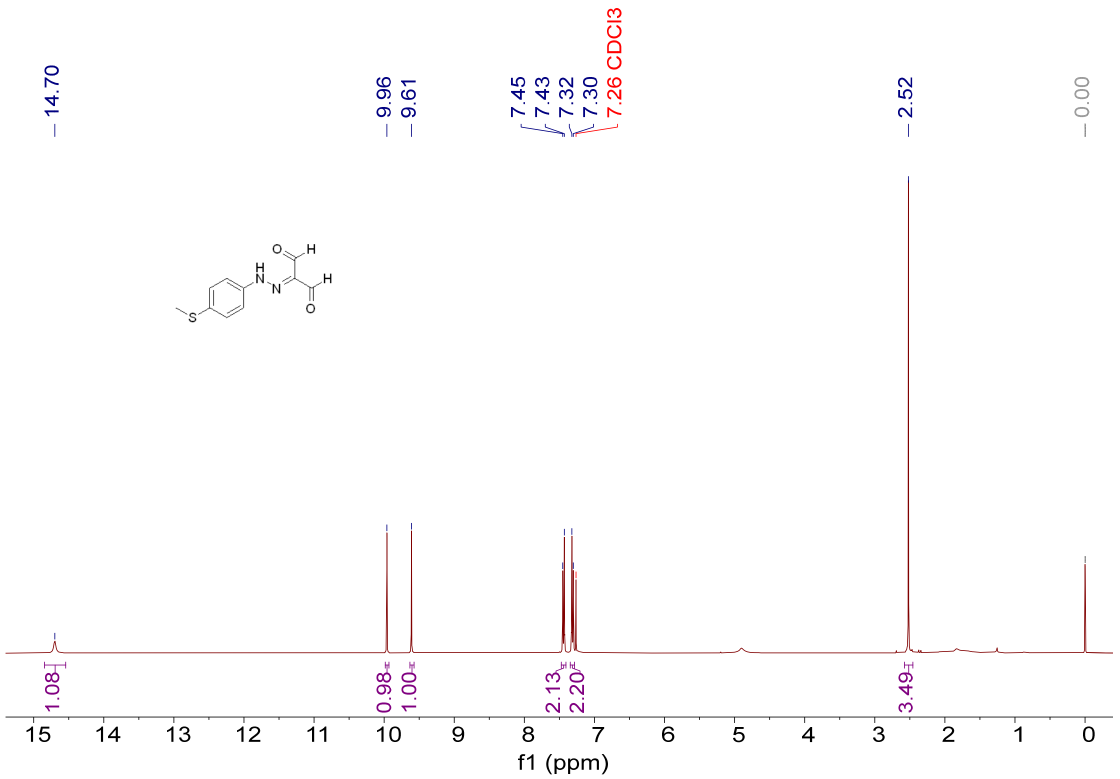
14.70
9.96
9.61
7.45
7.43
7.32
7.30
7.26 CDCl3
2.52
0.00
1.08
0.98
1.00
2.13
2.20
3.49
f1 (ppm)

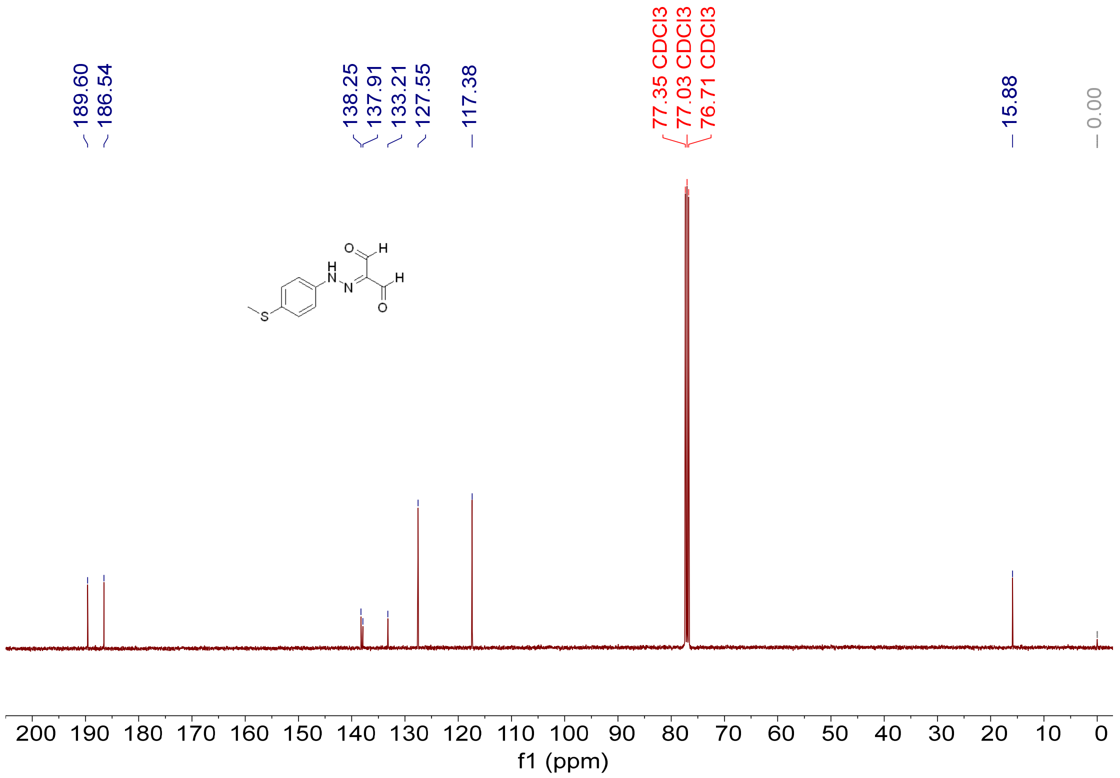
189.60
186.54
138.25
137.91
133.21
127.55
117.38
77.35 CDCl3
77.03 CDCl3
76.71 CDCl3
15.88
0.00
f1 (ppm)

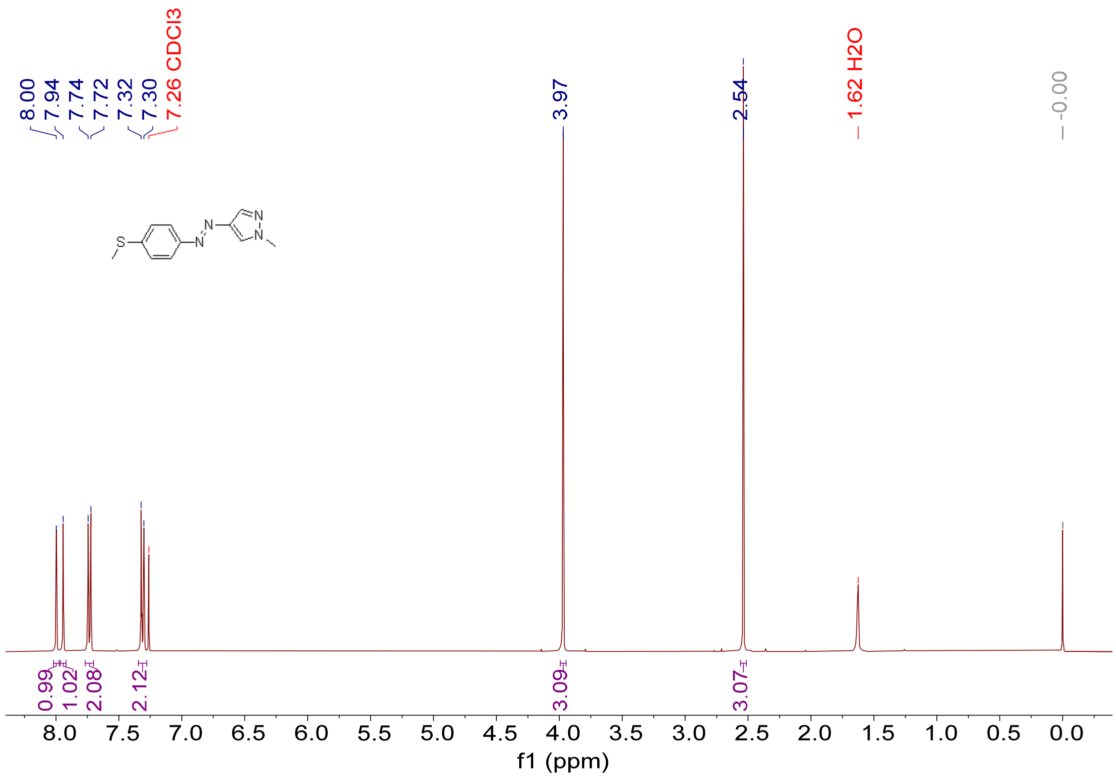
8.00
7.94
7.74
7.72
7.32
7.30
7.26 CDCl3
3.97
2.54
1.62 H2O
-0.00
0.99
1.02
2.08
2.12
3.09
3.07
S
N
N
N
N
f1 (ppm)

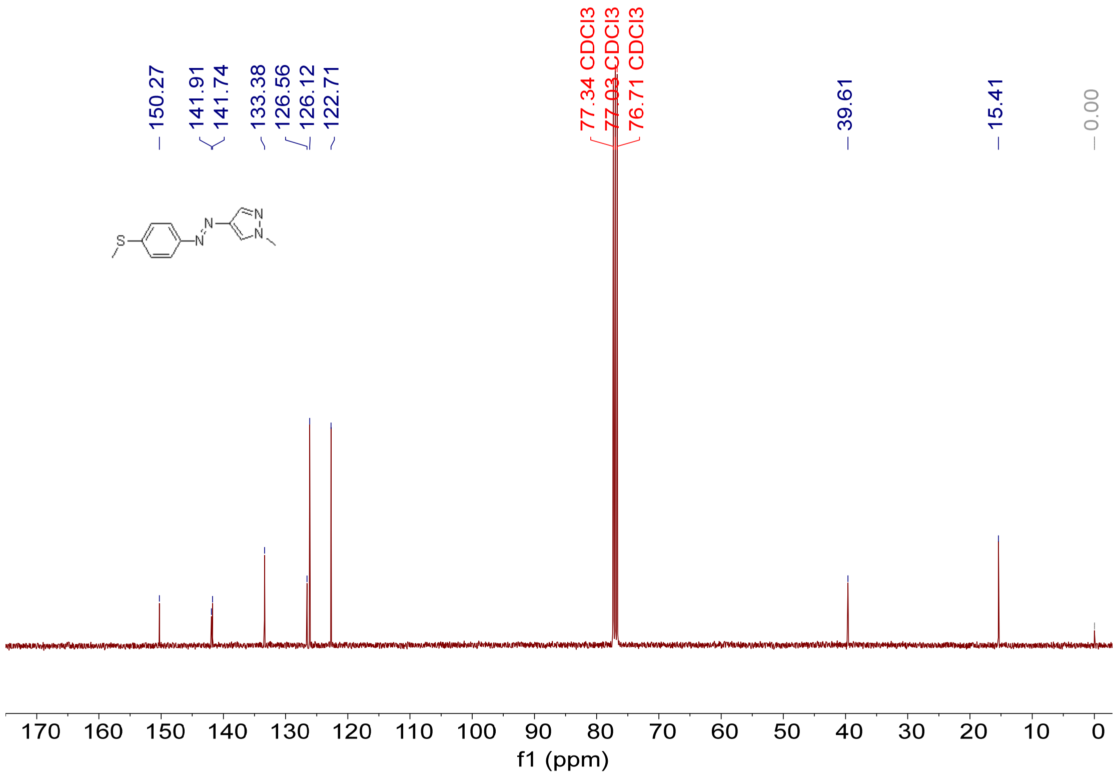
150.27
141.91
141.74
133.38
126.56
126.12
122.71
77.34 CDCl3
77.03 CDCl3
76.71 CDCl3
39.61
15.41
0.00
S
N
N
N
N
f1 (ppm)

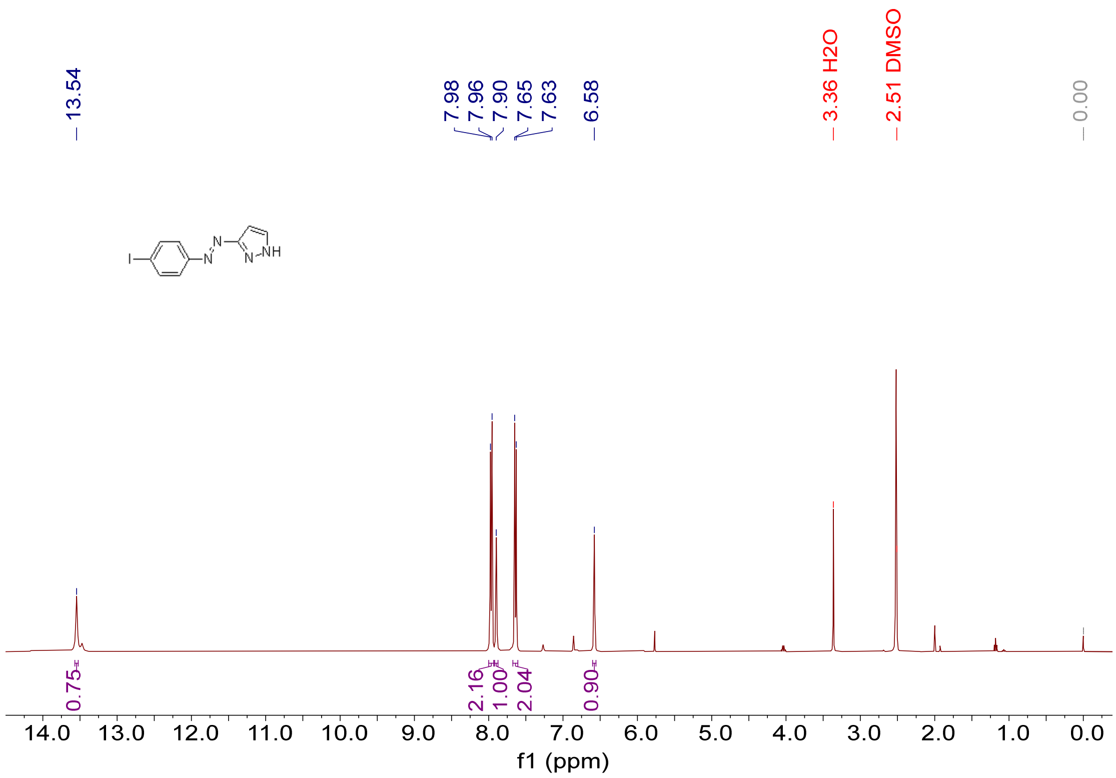
13.54
7.98
7.96
7.90
7.65
7.63
6.58
3.36 H2O
2.51 DMSO
0.00
0.75
2.16
1.00
2.04
0.90
14.0 13.0 12.0 11.0 10.0 9.0 8.0 7.0 6.0 5.0 4.0 3.0 2.0 1.0 0.0
f1 (ppm)

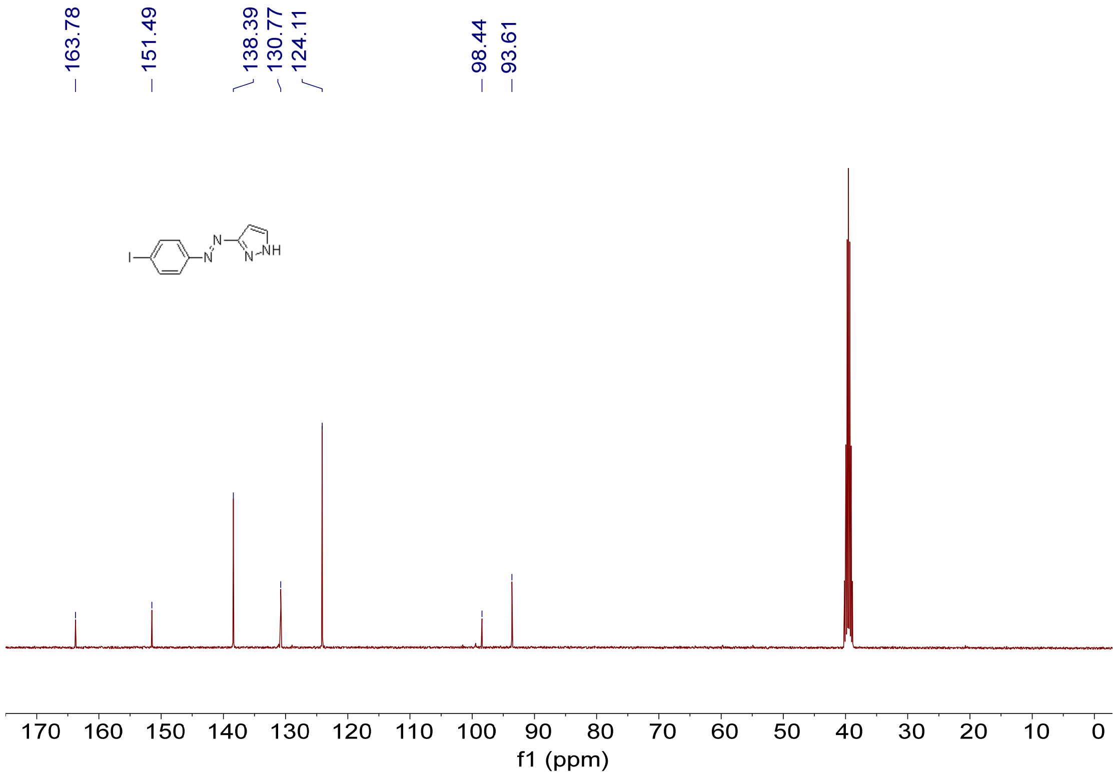
163.78
151.49
138.39
130.77
124.11
98.44
93.61
170 160 150 140 130 120 110 100 90 80 70 60 50 40 30 20 10 0
f1 (ppm)

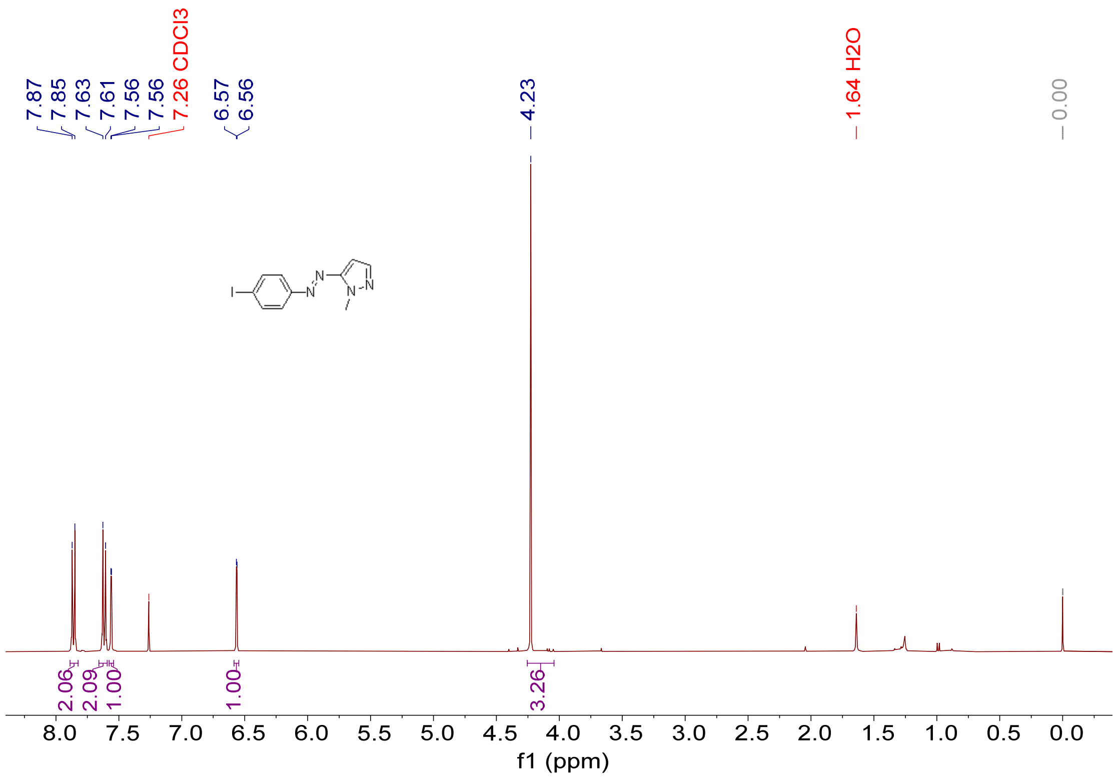
7.87
7.85
7.63
7.61
7.56
7.56
7.26 CDCl3
6.57
6.56
4.23
1.64 H2O
0.00
2.06
2.09
1.00
1.00
3.26
8.0 7.5 7.0 6.5 6.0 5.5 5.0 4.5 4.0 3.5 3.0 2.5 2.0 1.5 1.0 0.5 0.0
f1 (ppm)

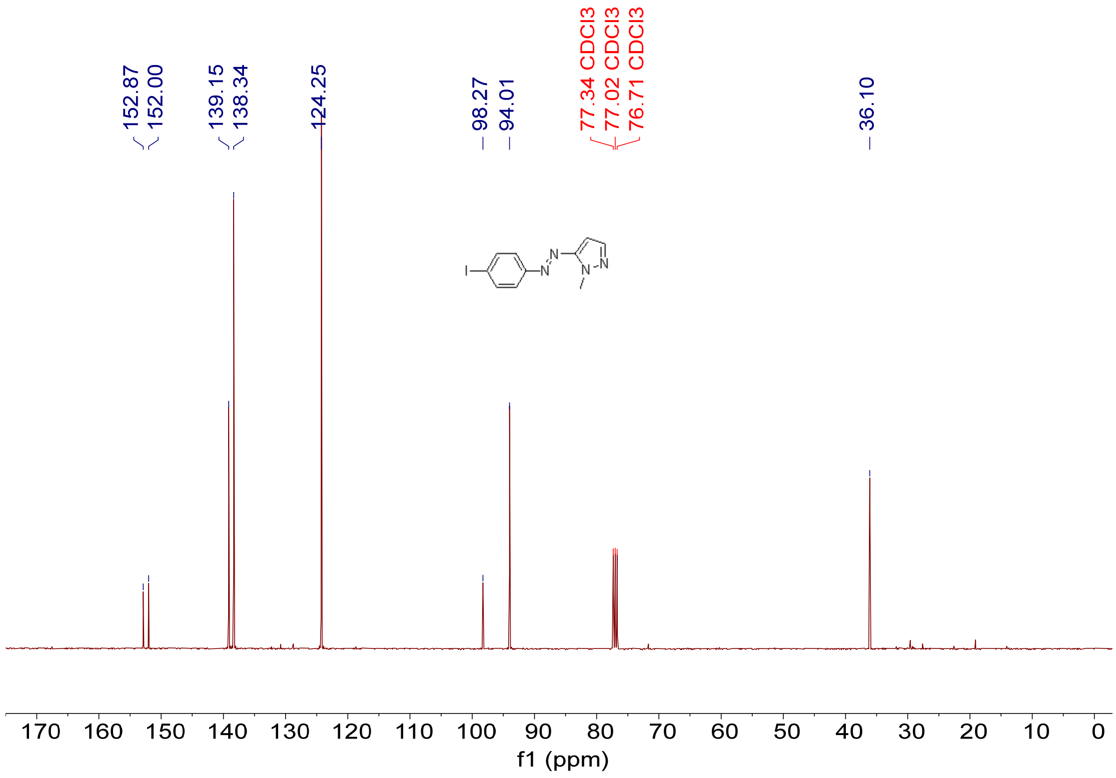
152.87
152.00
139.15
138.34
124.25
98.27
94.01
77.34 CDCl3
77.02 CDCl3
76.71 CDCl3
36.10
170 160 150 140 130 120 110 100 90 80 70 60 50 40 30 20 10 0
f1 (ppm)

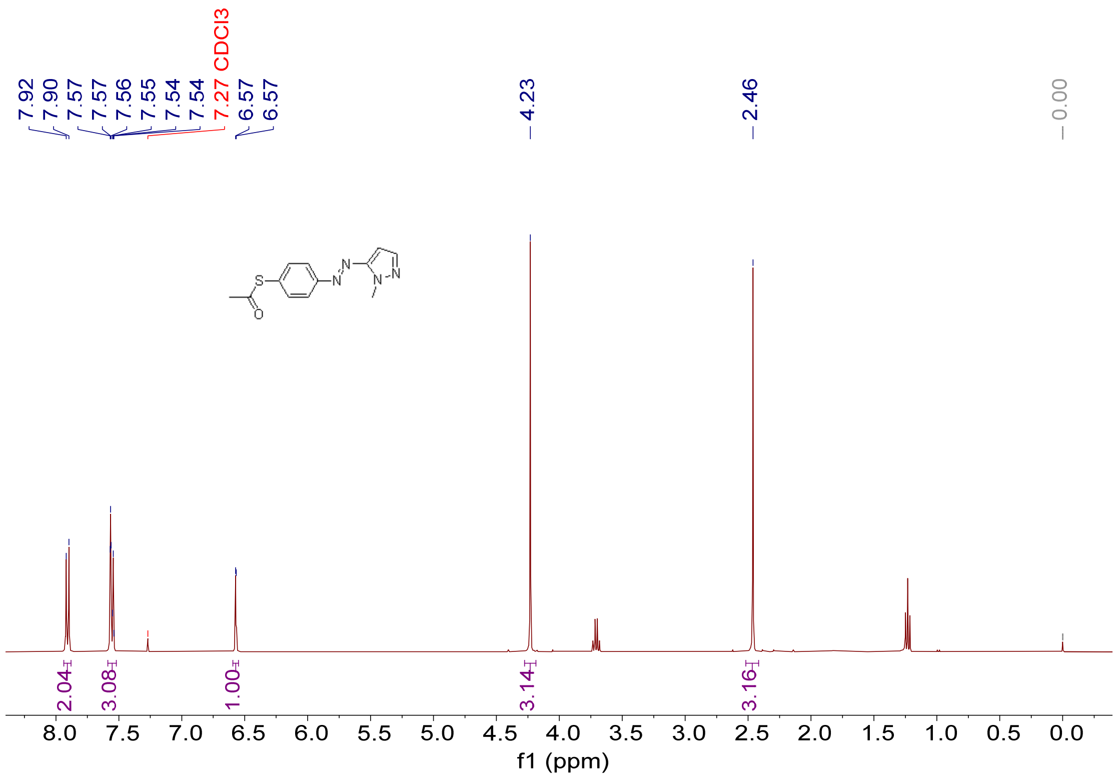
7.92
7.90
7.57
7.57
7.56
7.55
7.54
7.54
7.27 CDCl3
6.57
6.57
4.23
2.46
0.00
2.04
3.08
1.00
3.14
3.16
f1 (ppm)

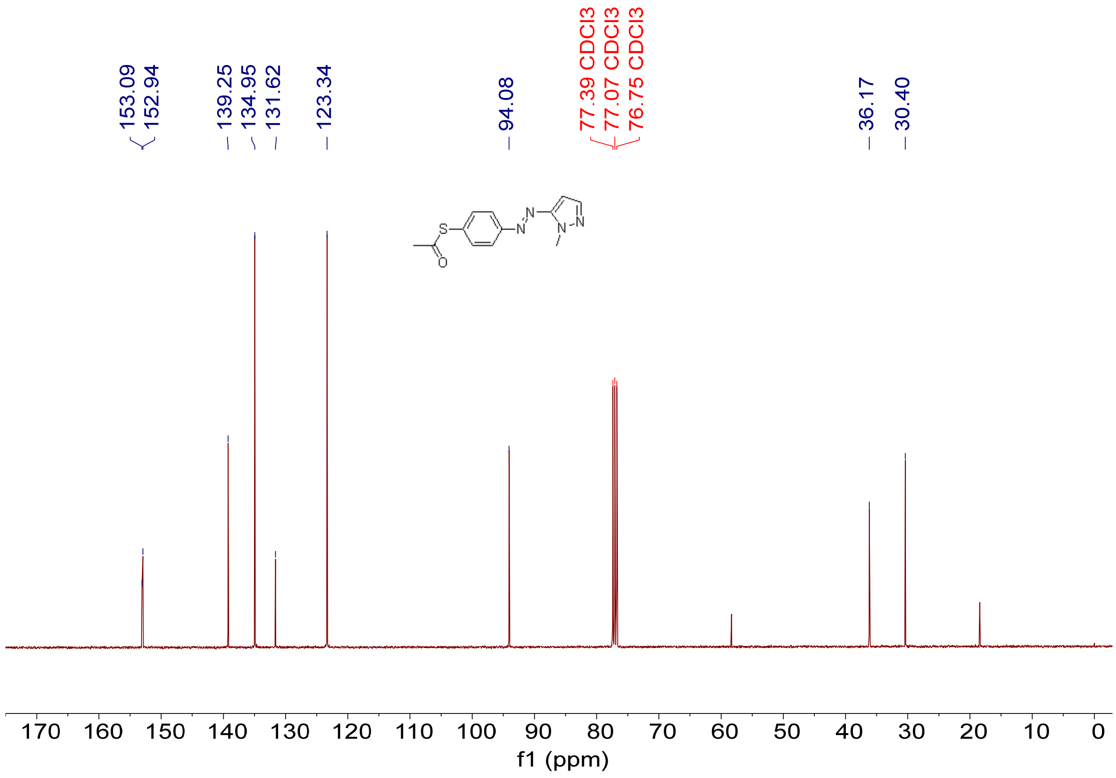
153.09
152.94
139.25
134.95
131.62
123.34
94.08
77.39 CDCl3
77.07 CDCl3
76.75 CDCl3
36.17
30.40
f1 (ppm)

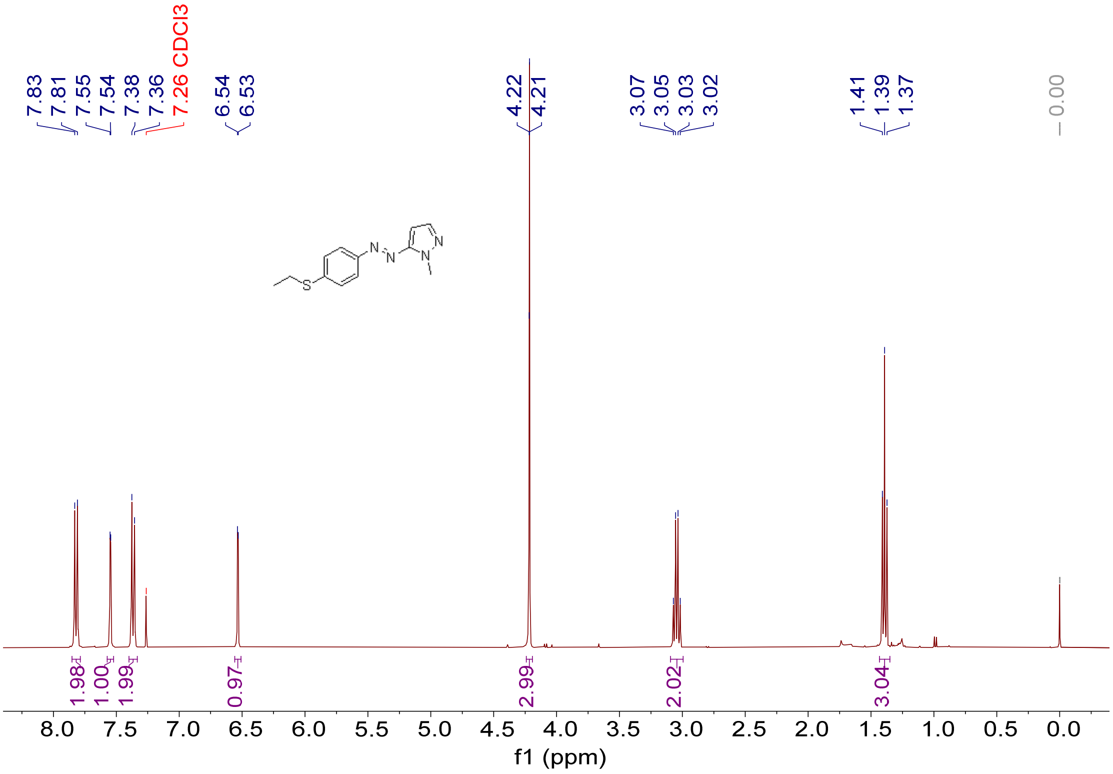
7.83
7.81
7.55
7.54
7.38
7.36
7.26 CDCl3
6.54
6.53
4.22
4.21
3.07
3.05
3.03
3.02
1.41
1.39
1.37
-0.00
N
N
N
N
S
1.98
1.00
1.99
0.97
2.99
2.02
3.04
8.0 7.5 7.0 6.5 6.0 5.5 5.0 4.5 4.0 3.5 3.0 2.5 2.0 1.5 1.0 0.5 0.0
f1 (ppm)

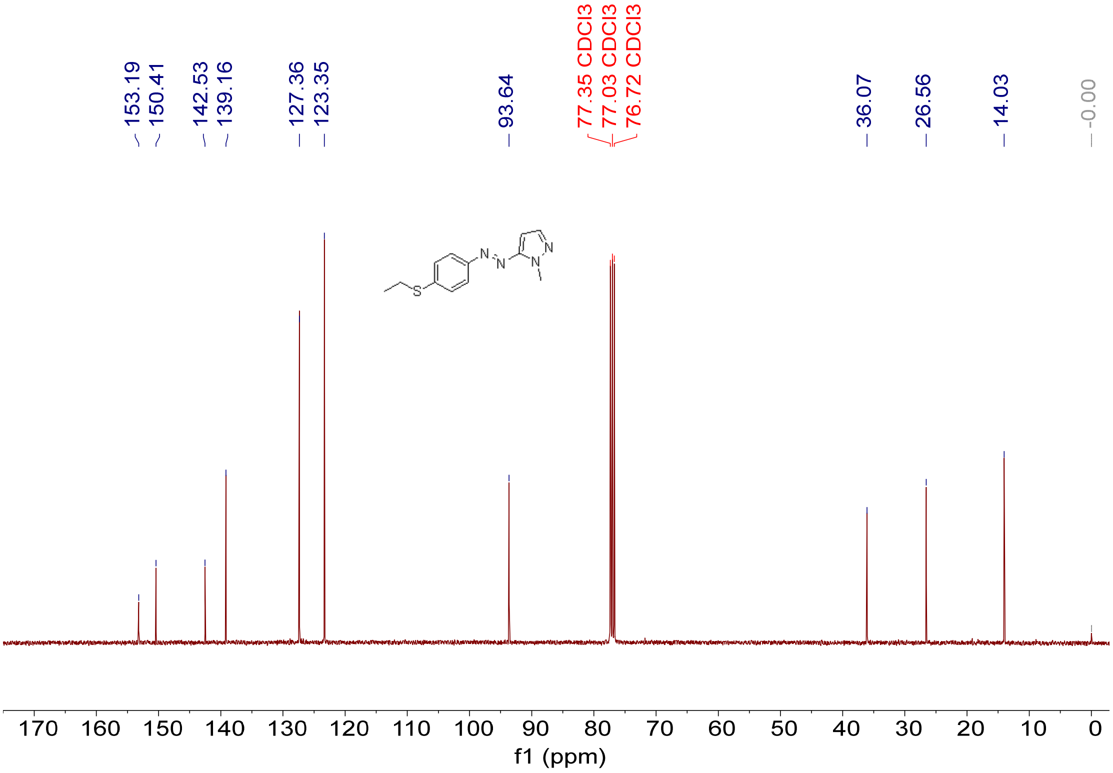
153.19
150.41
142.53
139.16
127.36
123.35
93.64
77.35 CDCl3
77.03 CDCl3
76.72 CDCl3
36.07
26.56
14.03
-0.00
N
N
N
N
S
170 160 150 140 130 120 110 100 90 80 70 60 50 40 30 20 10 0
f1 (ppm)

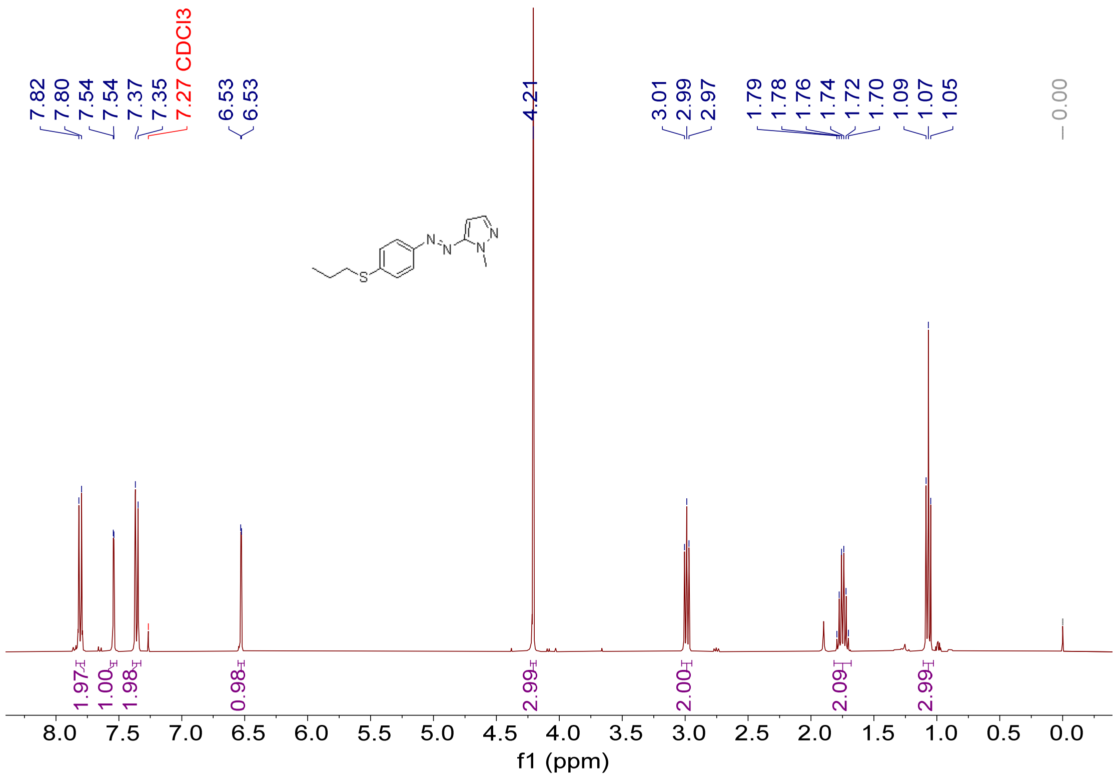
7.82
7.80
7.54
7.54
7.37
7.35
7.27 CDCl3
6.53
6.53
4.21
3.01
2.99
2.97
1.79
1.78
1.76
1.74
1.72
1.70
1.09
1.07
1.05
0.00
S
N
N
N
N
1.97
1.00
1.98
0.98
2.99
2.00
2.09
2.99
8.0 7.5 7.0 6.5 6.0 5.5 5.0 4.5 4.0 3.5 3.0 2.5 2.0 1.5 1.0 0.5 0.0
f1 (ppm)

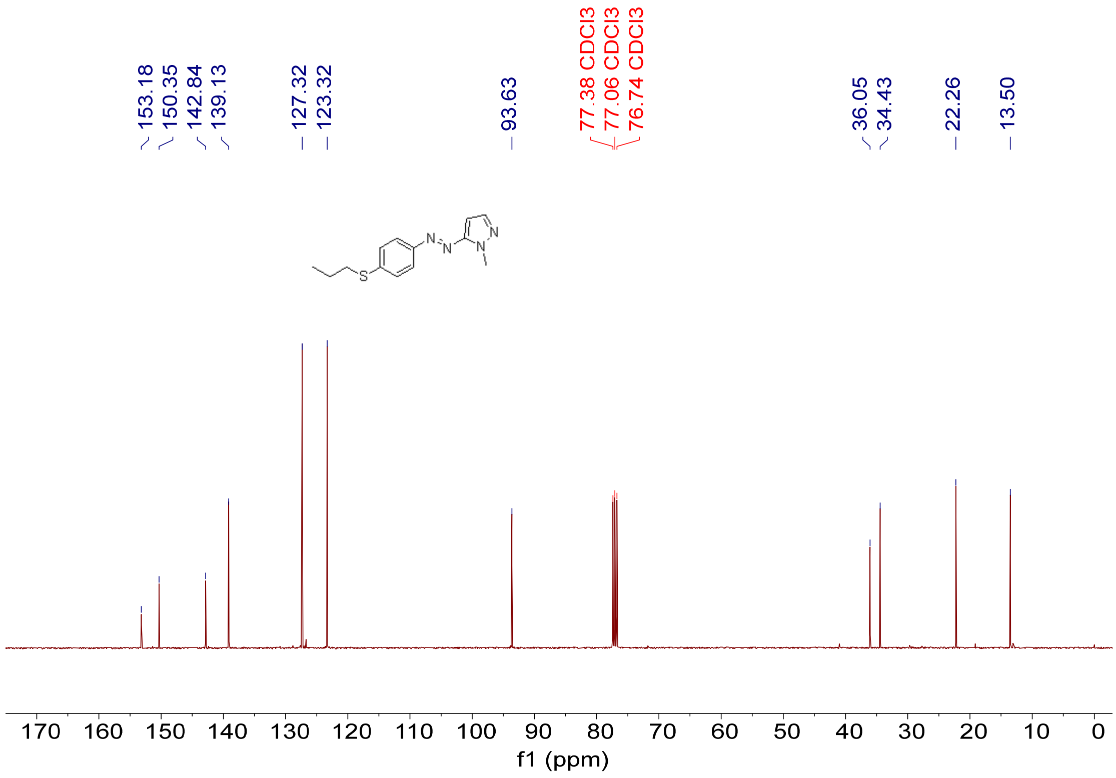
153.18
150.35
142.84
139.13
127.32
123.32
93.63
77.38 CDCl3
77.06 CDCl3
76.74 CDCl3
36.05
34.43
22.26
13.50
S
N
N
N
N
170 160 150 140 130 120 110 100 90 80 70 60 50 40 30 20 10 0
f1 (ppm)

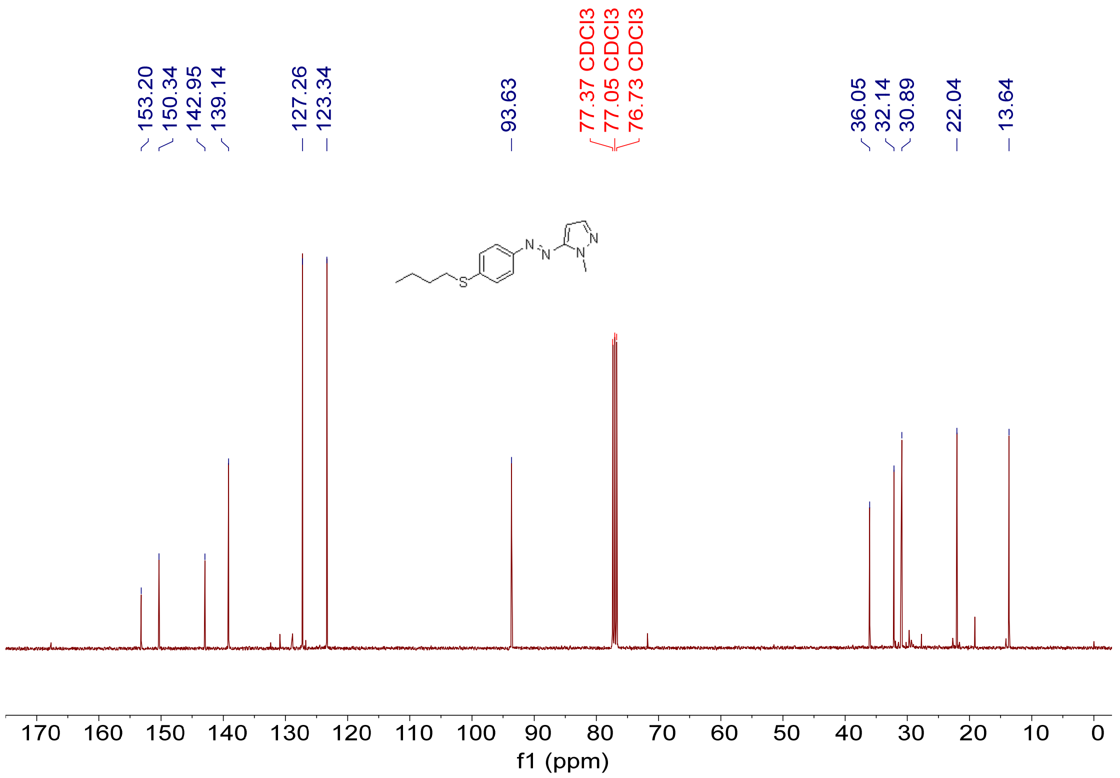
153.20
150.34
142.95
139.14
127.26
123.34
93.63
77.37 CDCl3
77.05 CDCl3
76.73 CDCl3
36.05
32.14
30.89
22.04
13.64
S
N
N
N
N
170 160 150 140 130 120 110 100 90 80 70 60 50 40 30 20 10 0
f1 (ppm)

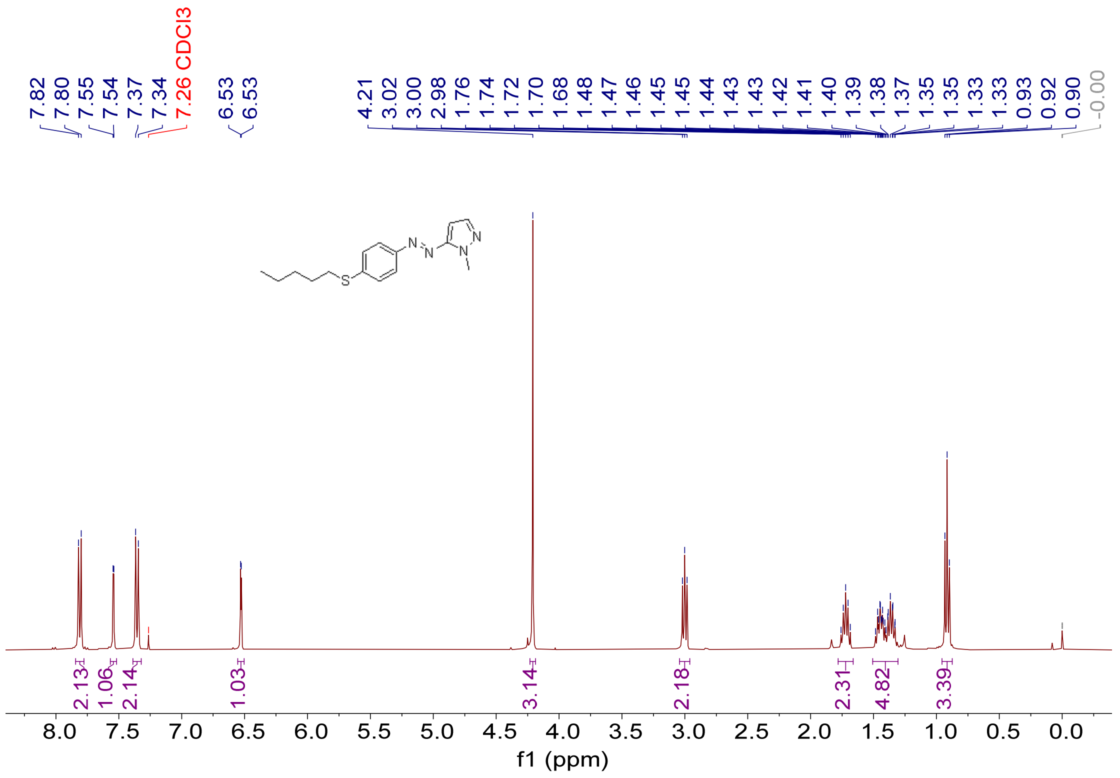
7.82
7.80
7.55
7.54
7.37
7.34
7.26 CDCl3
6.53
6.53
4.21
3.02
3.00
2.98
1.76
1.74
1.72
1.70
1.68
1.48
1.47
1.46
1.45
1.45
1.44
1.43
1.43
1.42
1.41
1.40
1.39
1.38
1.37
1.35
1.35
1.33
1.33
0.93
0.92
0.90
-0.00
S
N
N
N
N
2.13
1.06
2.14
1.03
3.14
2.18
2.31
4.82
3.39
8.0 7.5 7.0 6.5 6.0 5.5 5.0 4.5 4.0 3.5 3.0 2.5 2.0 1.5 1.0 0.5 0.0
f1 (ppm)

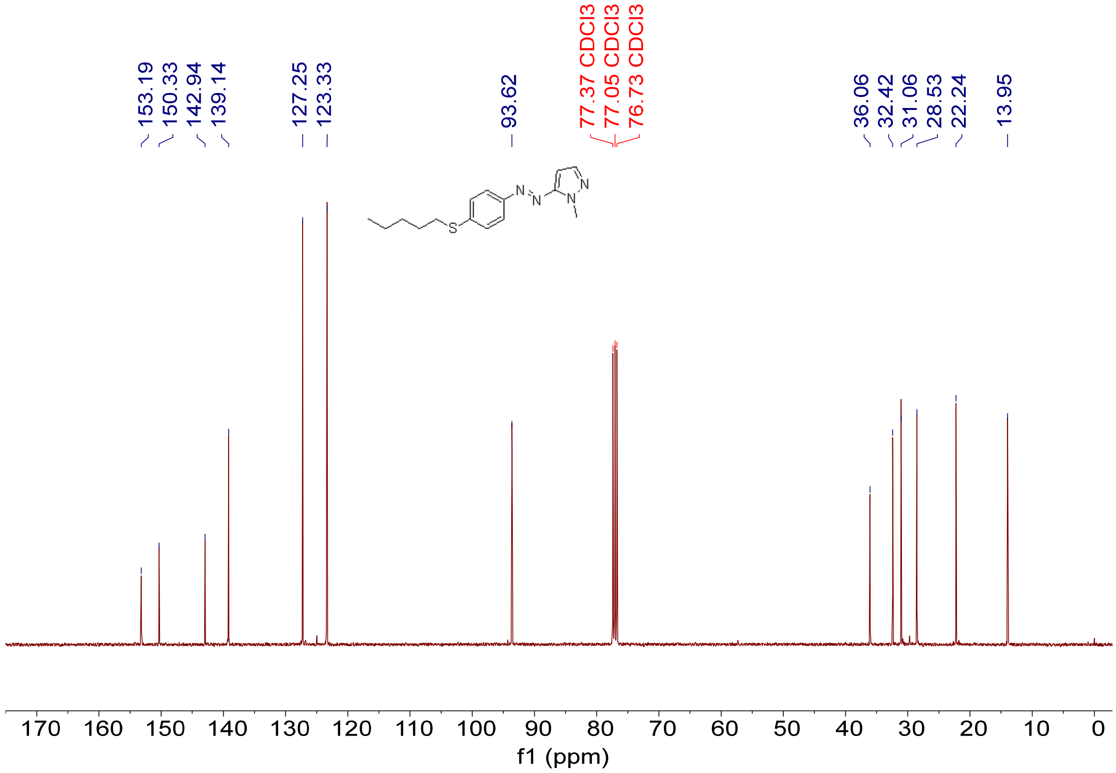
153.19
150.33
142.94
139.14
127.25
123.33
93.62
77.37 CDCl3
77.05 CDCl3
76.73 CDCl3
36.06
32.42
31.06
28.53
22.24
13.95
S
N
N
N
N
170 160 150 140 130 120 110 100 90 80 70 60 50 40 30 20 10 0
f1 (ppm)

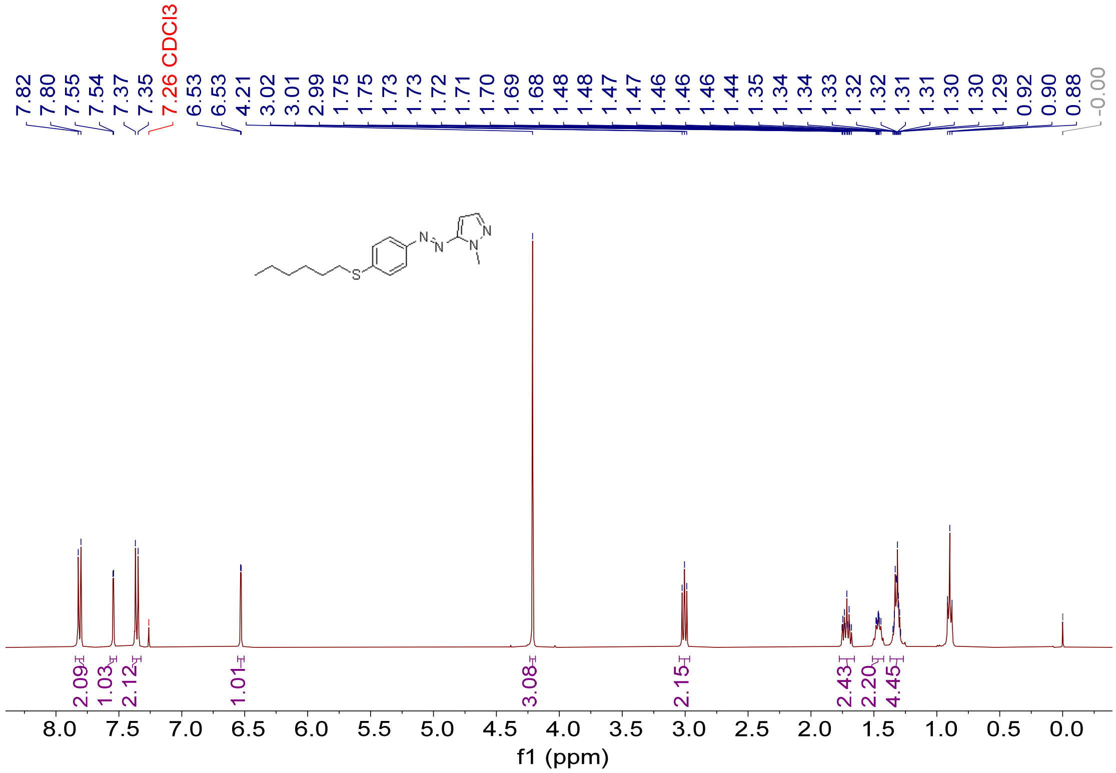
7.82
7.80
7.55
7.54
7.37
7.35
7.26 CDCl3
6.53
6.53
4.21
3.02
3.01
2.99
1.75
1.75
1.73
1.73
1.72
1.71
1.70
1.69
1.68
1.48
1.48
1.47
1.47
1.46
1.46
1.46
1.44
1.35
1.34
1.34
1.33
1.32
1.32
1.31
1.31
1.30
1.30
1.29
0.92
0.90
0.88
-0.00
S
N
N
N
N
2.09
1.03
2.12
1.01
3.08
2.15
2.43
2.20
4.45
8.0 7.5 7.0 6.5 6.0 5.5 5.0 4.5 4.0 3.5 3.0 2.5 2.0 1.5 1.0 0.5 0.0
f1 (ppm)

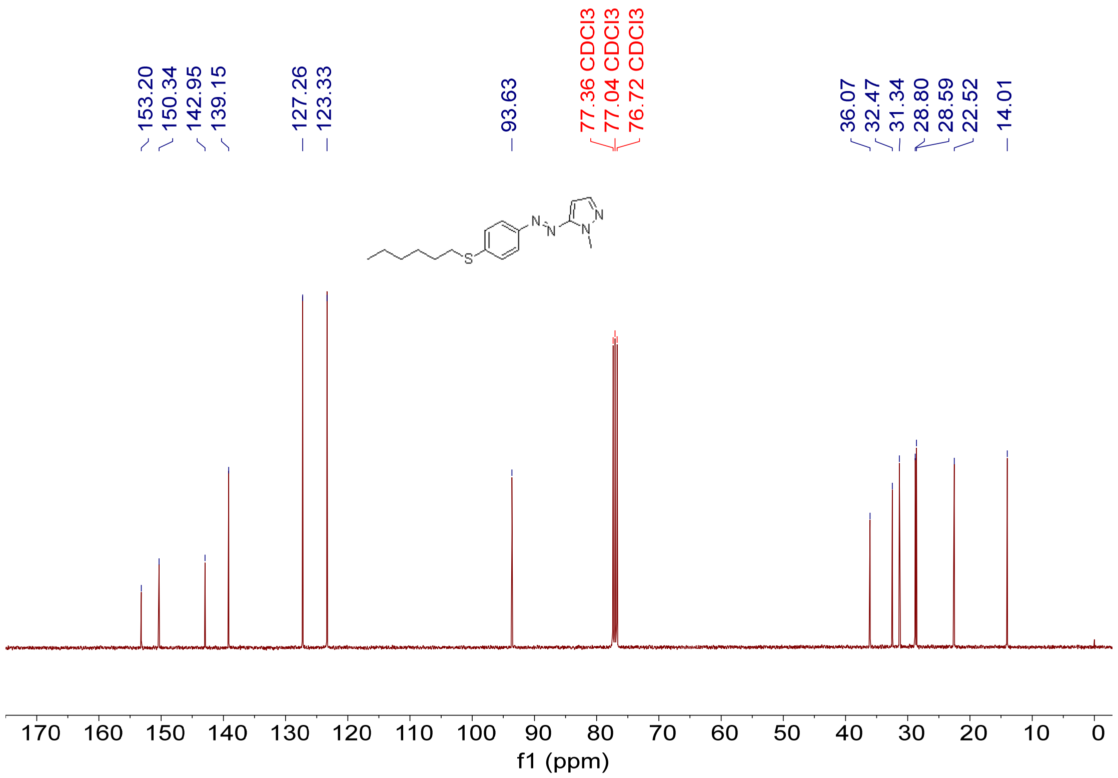

153.20
150.34
142.95
139.15
127.26
123.33
93.63
77.36 CDCl3
77.04 CDCl3
76.72 CDCl3
36.07
32.47
31.34
28.80
28.59
22.52
14.01
N
N
N
N
S
170 160 150 140 130 120 110 100 90 80 70 60 50 40 30 20 10 0
f1 (ppm)

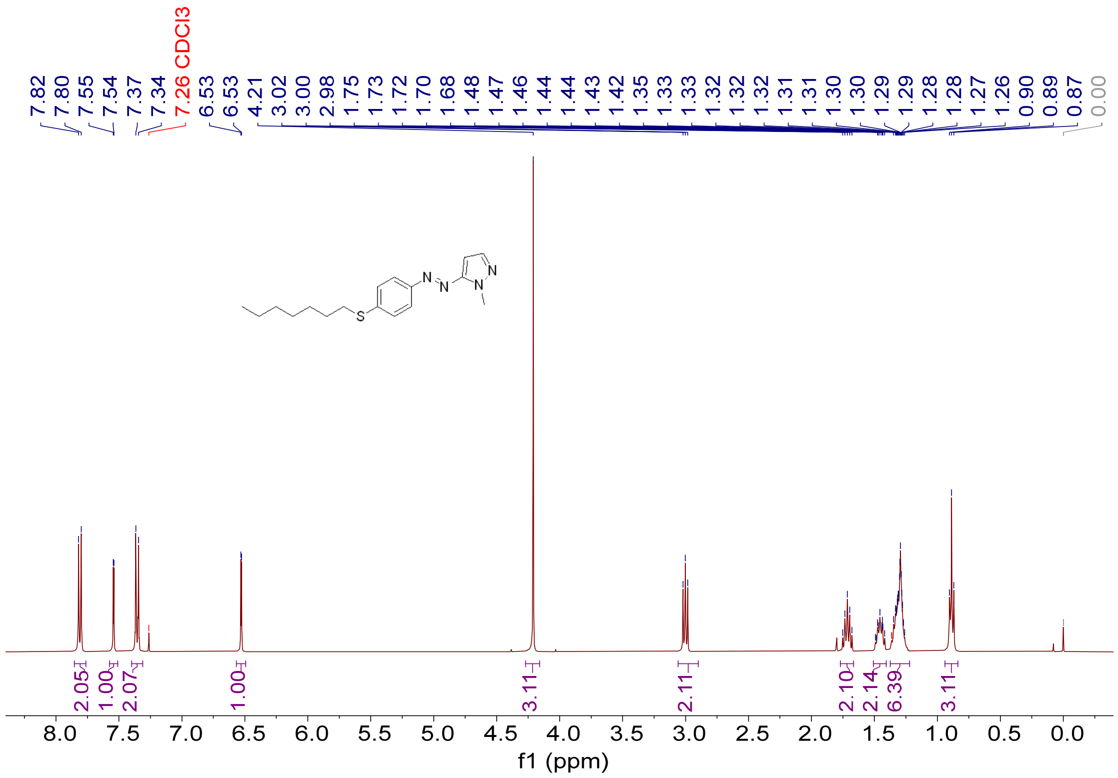

7.82
7.80
7.55
7.54
7.37
7.34
7.26 CDCl3
6.53
6.53
4.21
3.02
3.00
2.98
1.75
1.73
1.72
1.70
1.68
1.48
1.47
1.46
1.44
1.44
1.43
1.42
1.35
1.33
1.33
1.32
1.32
1.32
1.31
1.31
1.30
1.30
1.29
1.29
1.28
1.28
1.27
1.26
0.90
0.89
0.87
0.00
N
N
N
N
S
2.05
1.00
2.07
1.00
3.11
2.11
2.10
2.14
6.39
3.11
8.0 7.5 7.0 6.5 6.0 5.5 5.0 4.5 4.0 3.5 3.0 2.5 2.0 1.5 1.0 0.5 0.0
f1 (ppm)

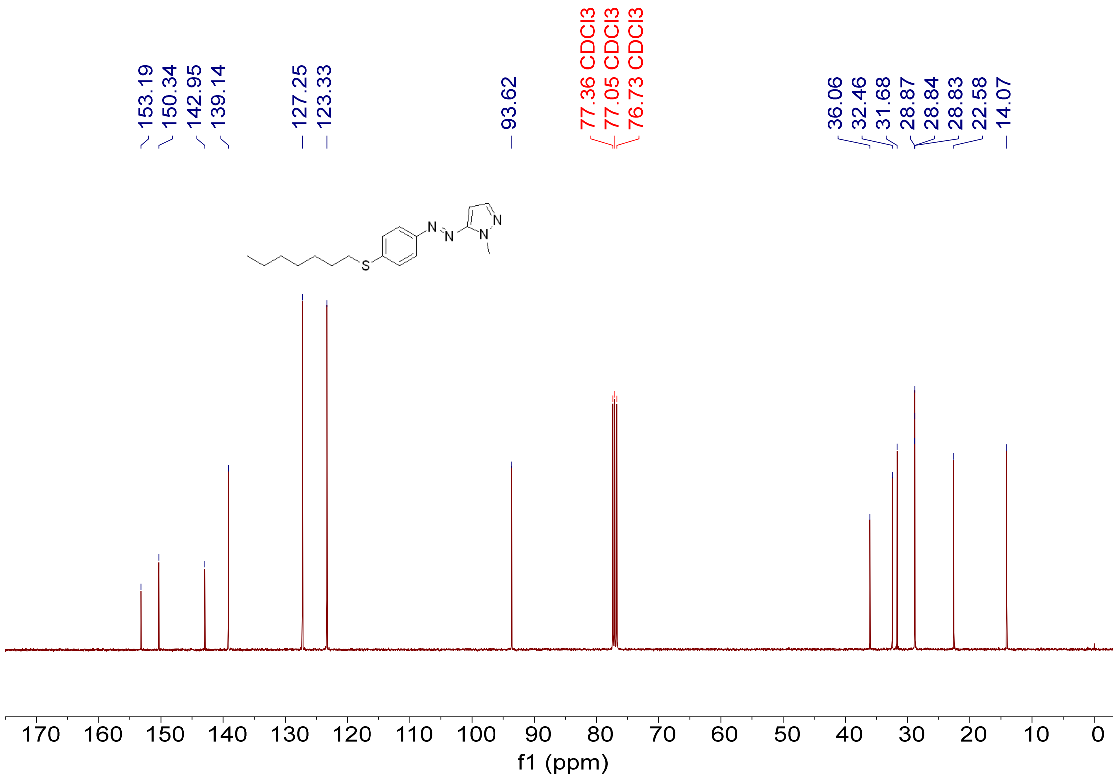
153.19
150.34
142.95
139.14
127.25
123.33
93.62
77.36 CDCl3
77.05 CDCl3
76.73 CDCl3
36.06
32.46
31.68
28.87
28.84
28.83
22.58
14.07
170 160 150 140 130 120 110 100 90 80 70 60 50 40 30 20 10 0
f1 (ppm)

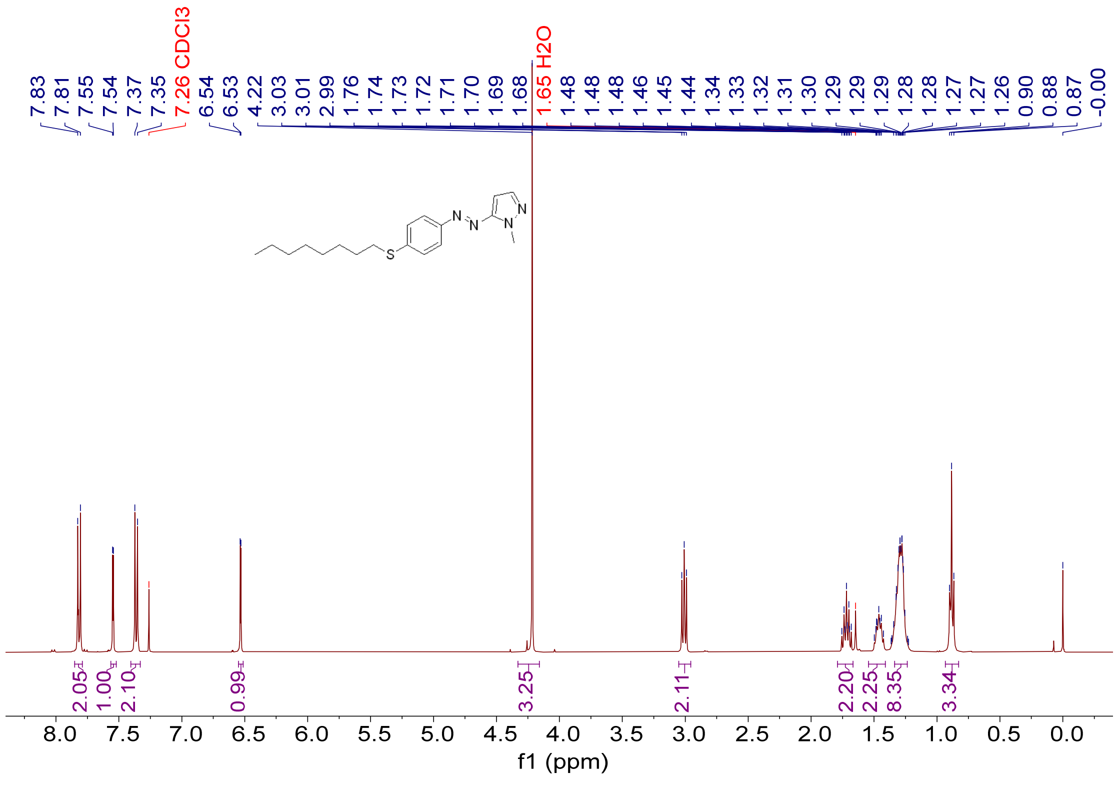
7.83
7.81
7.55
7.54
7.37
7.35
7.26 CDCl3
6.54
6.53
4.22
3.03
3.01
2.99
1.76
1.74
1.73
1.72
1.71
1.70
1.69
1.68
1.65 H2O
1.48
1.48
1.48
1.46
1.45
1.44
1.34
1.33
1.32
1.31
1.30
1.29
1.29
1.29
1.28
1.28
1.27
1.27
1.26
0.90
0.88
0.87
-0.00
2.05
1.00
2.10
0.99
3.25
2.11
2.20
2.25
8.35
3.34
8.0 7.5 7.0 6.5 6.0 5.5 5.0 4.5 4.0 3.5 3.0 2.5 2.0 1.5 1.0 0.5 0.0
f1 (ppm)

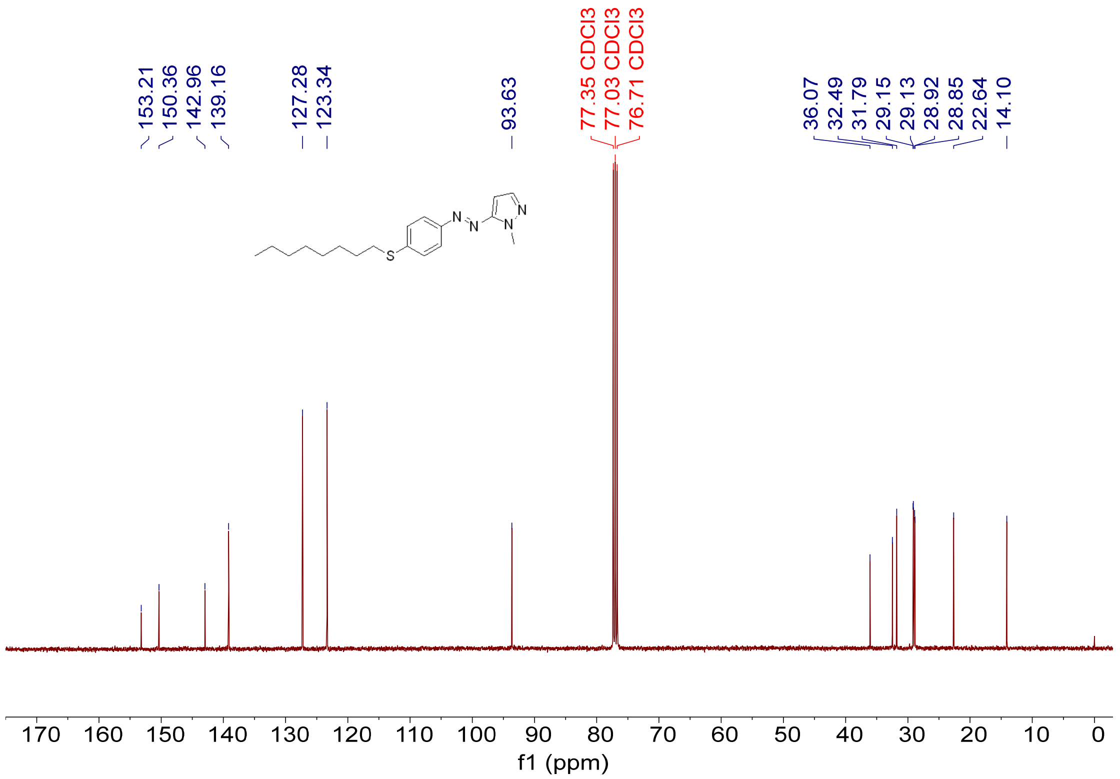
153.21
150.36
142.96
139.16
127.28
123.34
93.63
77.35 CDCl3
77.03 CDCl3
76.71 CDCl3
36.07
32.49
31.79
29.15
29.13
28.92
28.85
22.64
14.10
N
N
N
N
S
170 160 150 140 130 120 110 100 90 80 70 60 50 40 30 20 10 0
f1 (ppm)

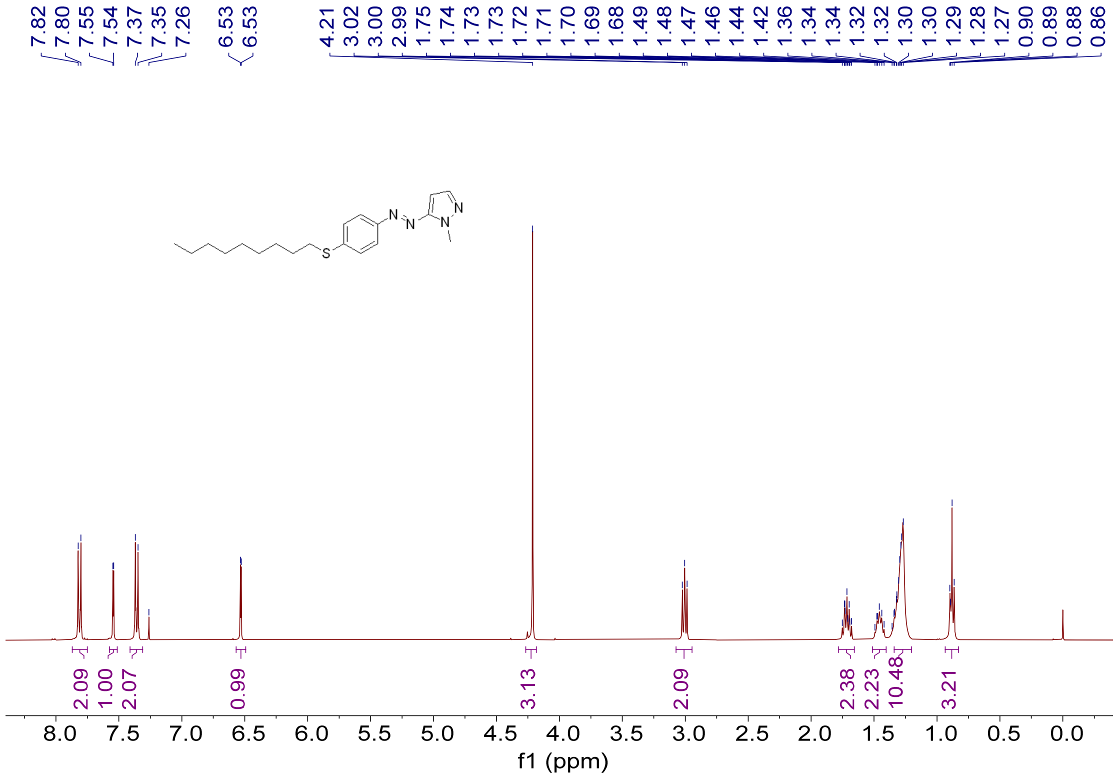
7.82
7.80
7.55
7.54
7.37
7.35
7.26
6.53
6.53
4.21
3.02
3.00
2.99
1.75
1.74
1.73
1.72
1.71
1.70
1.69
1.68
1.49
1.48
1.47
1.46
1.44
1.42
1.36
1.34
1.34
1.32
1.32
1.30
1.30
1.29
1.28
1.27
0.90
0.89
0.88
0.86
N
N
N
N
S
2.09
1.00
2.07
0.99
3.13
2.09
2.38
2.23
10.48
3.21
8.0 7.5 7.0 6.5 6.0 5.5 5.0 4.5 4.0 3.5 3.0 2.5 2.0 1.5 1.0 0.5 0.0
f1 (ppm)

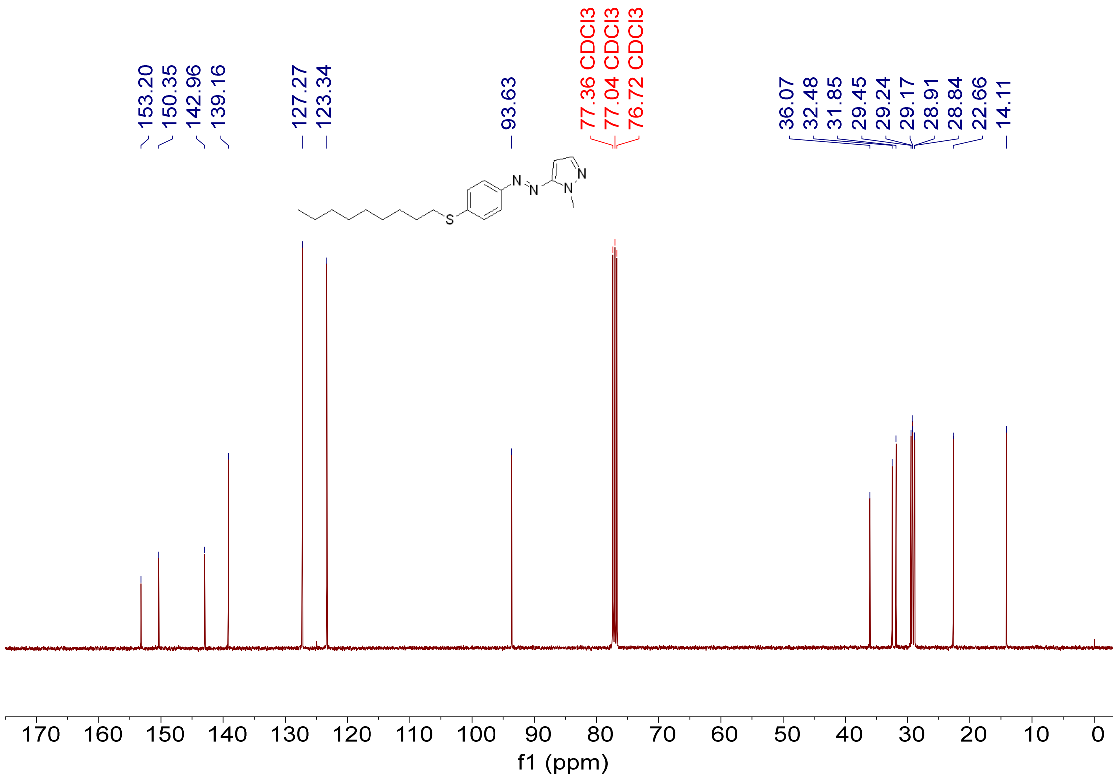
153.20
150.35
142.96
139.16
127.27
123.34
93.63
77.36 CDCl3
77.04 CDCl3
76.72 CDCl3
36.07
32.48
31.85
29.45
29.24
29.17
28.91
28.84
22.66
14.11
N
N
N
N
S
170 160 150 140 130 120 110 100 90 80 70 60 50 40 30 20 10 0
f1 (ppm)

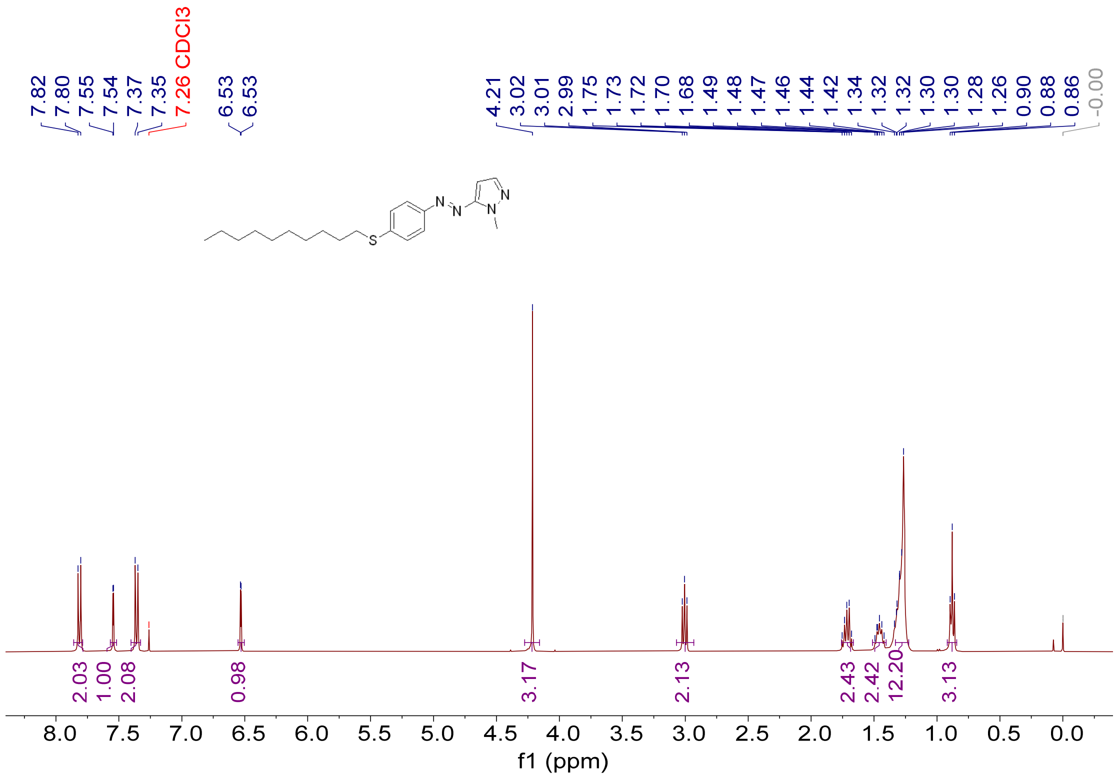
7.82
7.80
7.55
7.54
7.37
7.35
7.26 CDCl3
6.53
6.53
4.21
3.02
3.01
2.99
1.75
1.73
1.72
1.70
1.68
1.49
1.48
1.47
1.46
1.44
1.42
1.34
1.32
1.32
1.30
1.30
1.28
1.26
0.90
0.88
0.86
-0.00
N
N
N
N
S
2.03
1.00
2.08
0.98
3.17
2.13
2.43
2.42
12.20
3.13
8.0 7.5 7.0 6.5 6.0 5.5 5.0 4.5 4.0 3.5 3.0 2.5 2.0 1.5 1.0 0.5 0.0
f1 (ppm)

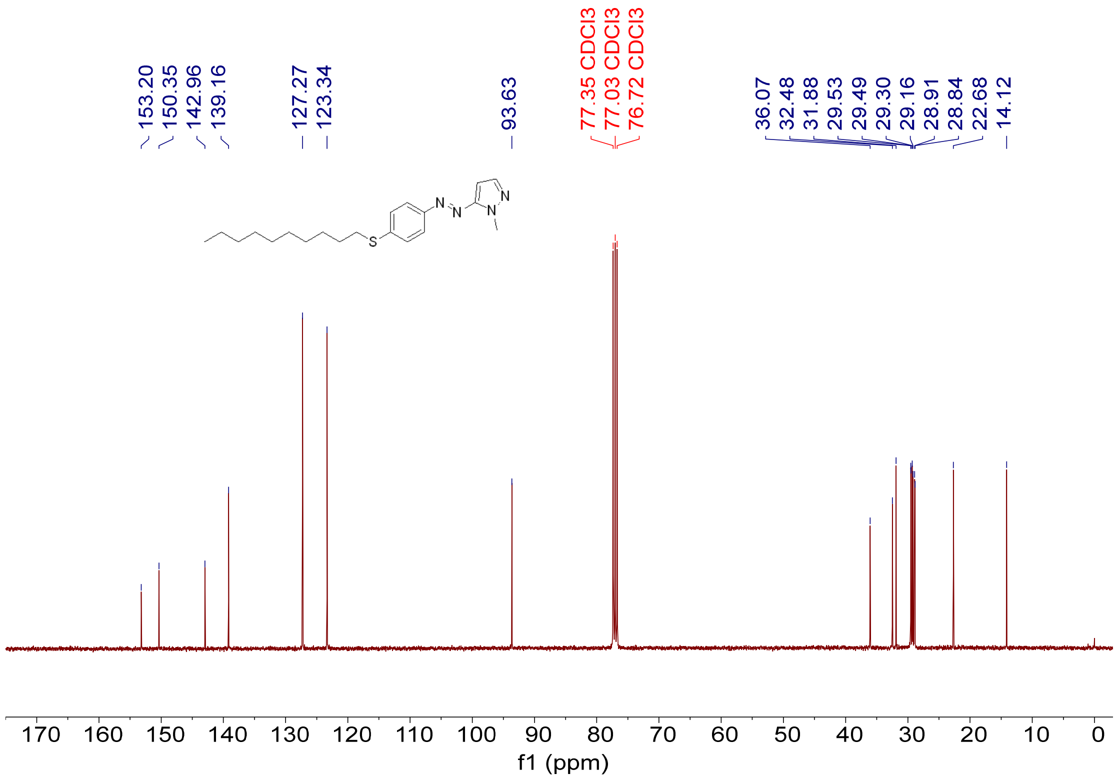
153.20
150.35
142.96
139.16
127.27
123.34
93.63
77.35 CDCl3
77.03 CDCl3
76.72 CDCl3
36.07
32.48
31.88
29.53
29.49
29.30
29.16
28.91
28.84
22.68
14.12
N
N
N
N
S
170 160 150 140 130 120 110 100 90 80 70 60 50 40 30 20 10 0
f1 (ppm)

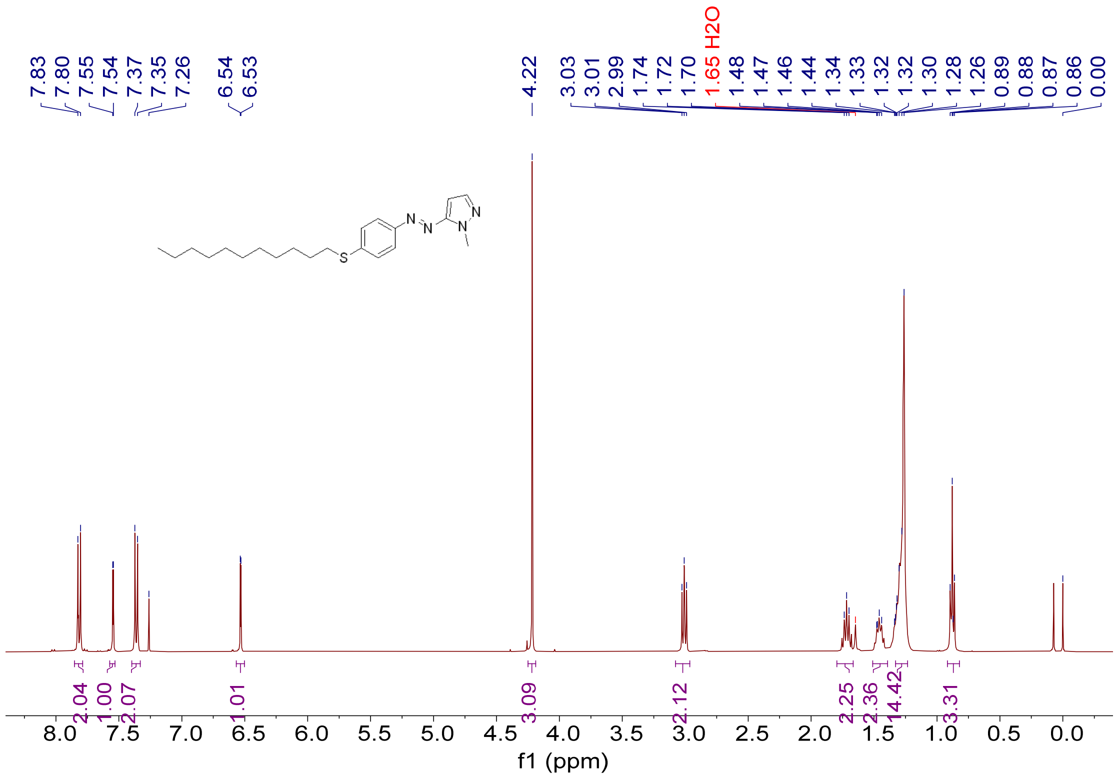
7.83
7.80
7.55
7.54
7.37
7.35
7.26
6.54
6.53
4.22
3.03
3.01
2.99
1.74
1.72
1.70
1.65 H2O
1.48
1.47
1.46
1.44
1.34
1.33
1.32
1.32
1.30
1.28
1.26
0.89
0.88
0.87
0.86
0.00
N
N
N
N
S
2.04
1.00
2.07
1.01
3.09
2.12
2.25
2.36
14.42
3.31
8.0 7.5 7.0 6.5 6.0 5.5 5.0 4.5 4.0 3.5 3.0 2.5 2.0 1.5 1.0 0.5 0.0
f1 (ppm)

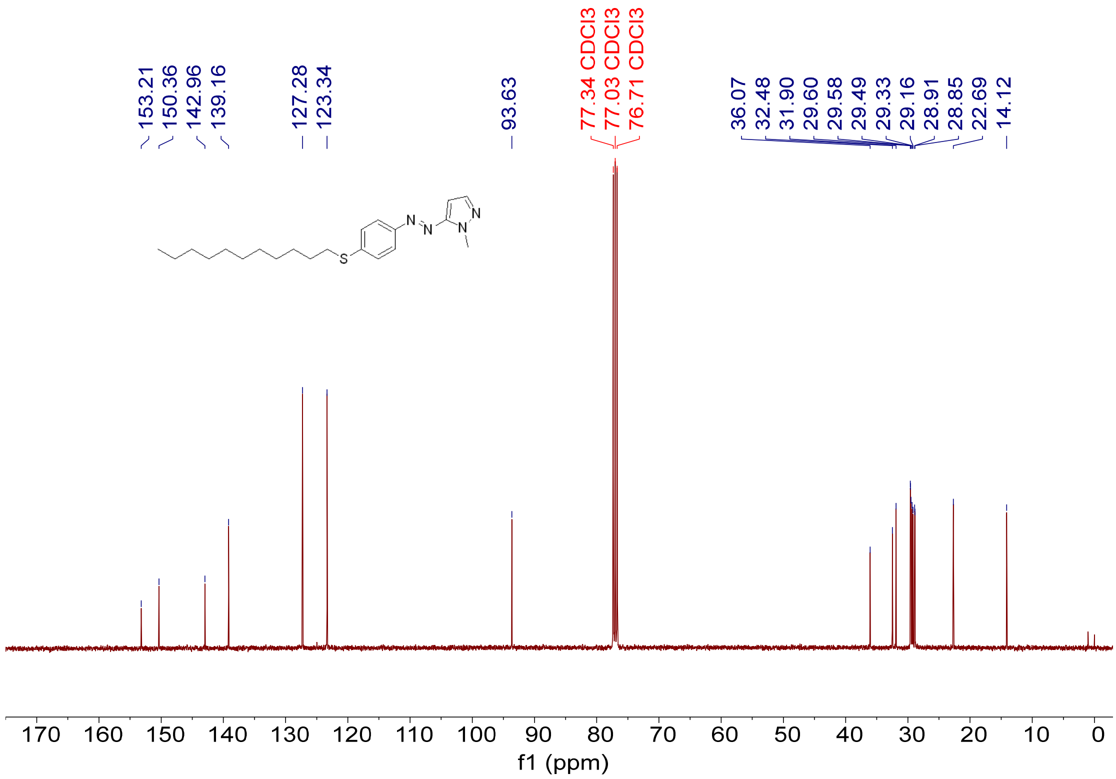
153.21
150.36
142.96
139.16
127.28
123.34
93.63
77.34 CDCl3
77.03 CDCl3
76.71 CDCl3
36.07
32.48
31.90
29.60
29.58
29.49
29.33
29.16
28.91
28.85
22.69
14.12
S
N
N
N
N
170 160 150 140 130 120 110 100 90 80 70 60 50 40 30 20 10 0
f1 (ppm)

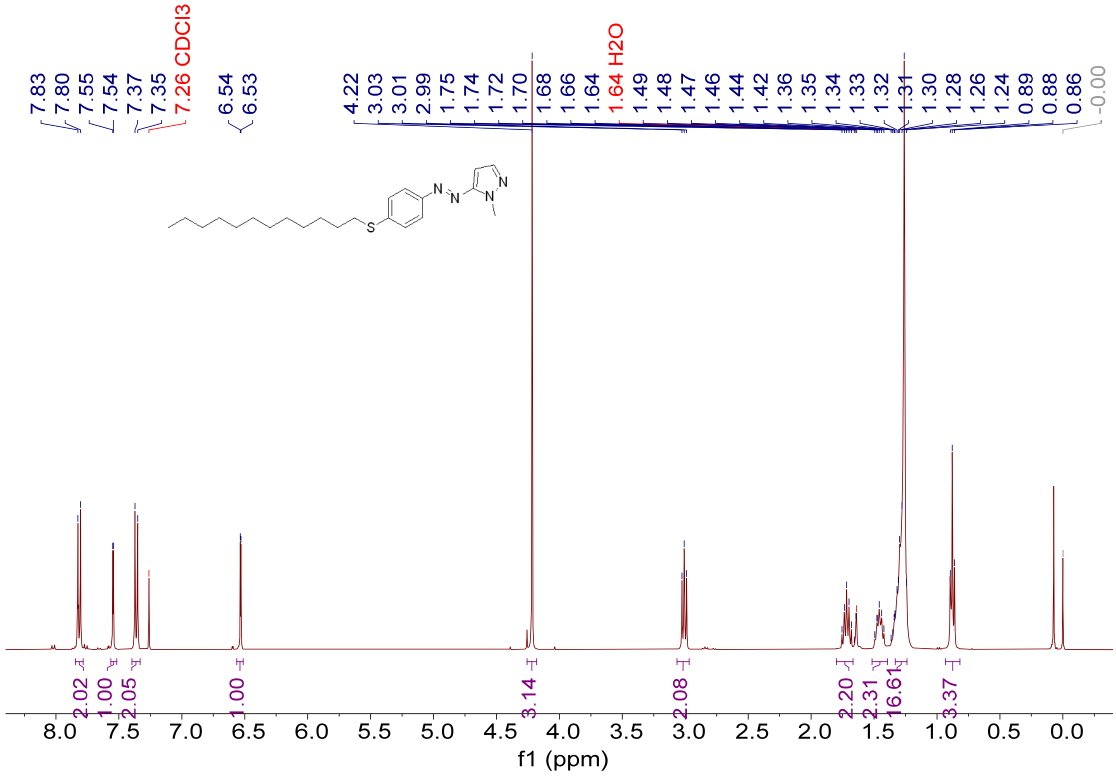
7.83
7.80
7.55
7.54
7.37
7.35
7.26 CDCl3
6.54
6.53
4.22
3.03
3.01
2.99
1.75
1.74
1.72
1.70
1.68
1.66
1.64
1.64 H2O
1.49
1.48
1.47
1.46
1.44
1.42
1.36
1.35
1.34
1.33
1.32
1.31
1.30
1.28
1.26
1.24
0.89
0.88
0.86
-0.00
S
N
N
N
N
2.02
1.00
2.05
1.00
3.14
2.08
2.20
2.31
16.61
3.37
8.0 7.5 7.0 6.5 6.0 5.5 5.0 4.5 4.0 3.5 3.0 2.5 2.0 1.5 1.0 0.5 0.0
f1 (ppm)

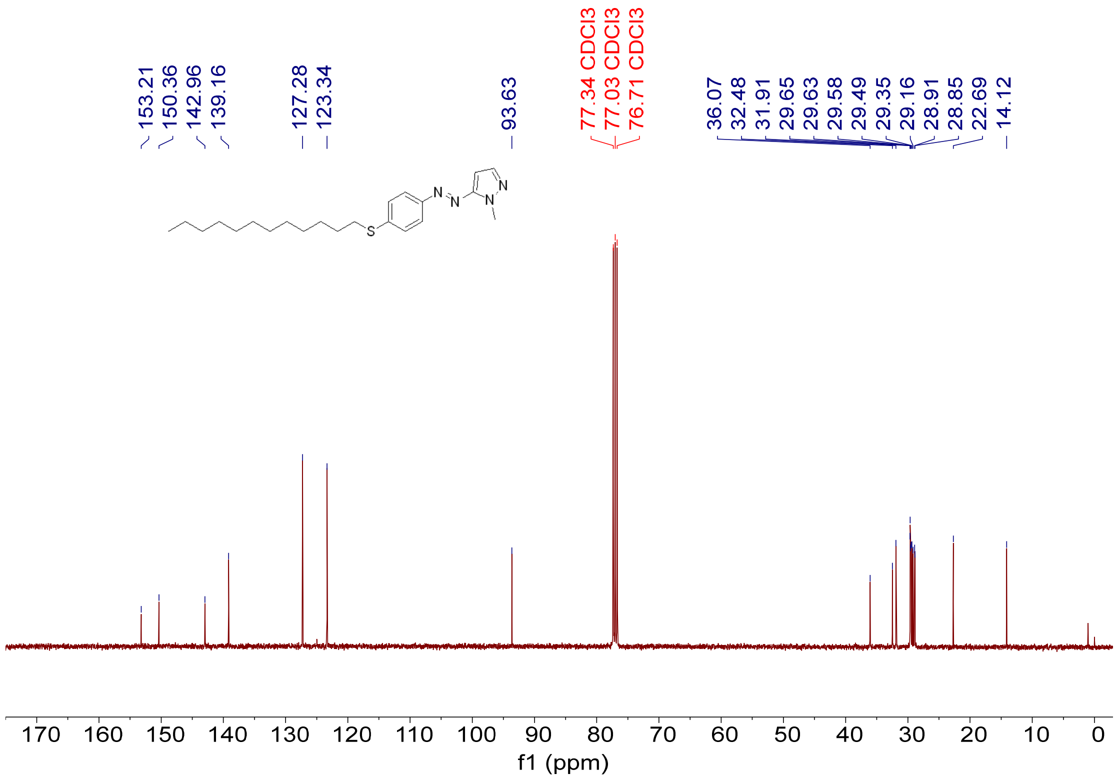
153.21
150.36
142.96
139.16
127.28
123.34
93.63
77.34 CDCl3
77.03 CDCl3
76.71 CDCl3
36.07
32.48
31.91
29.65
29.63
29.58
29.49
29.35
29.16
28.91
28.85
22.69
14.12
170 160 150 140 130 120 110 100 90 80 70 60 50 40 30 20 10 0
f1 (ppm)

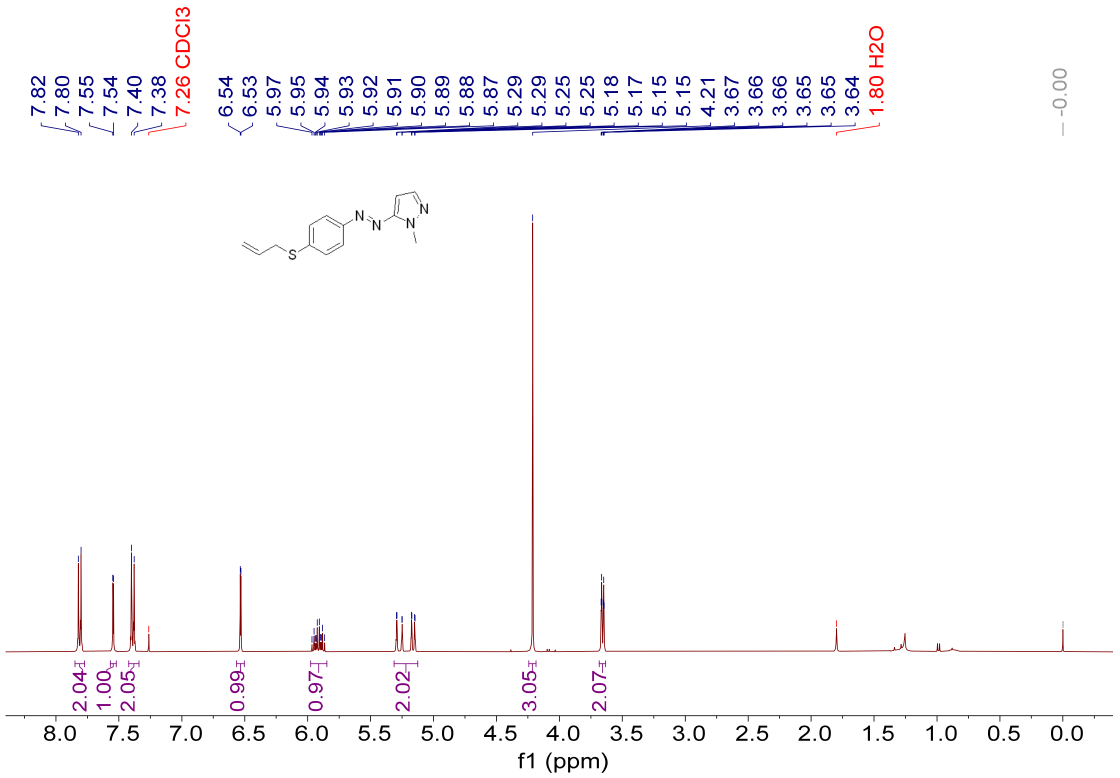
7.82
7.80
7.55
7.54
7.40
7.38
7.26 CDCl3
6.54
6.53
5.97
5.95
5.94
5.93
5.92
5.91
5.90
5.89
5.88
5.87
5.29
5.29
5.25
5.25
5.18
5.17
5.15
5.15
4.21
3.67
3.66
3.66
3.65
3.65
3.64
1.80 H2O
-0.00
2.04
1.00
2.05
0.99
0.97
2.02
3.05
2.07
8.0 7.5 7.0 6.5 6.0 5.5 5.0 4.5 4.0 3.5 3.0 2.5 2.0 1.5 1.0 0.5 0.0
f1 (ppm)

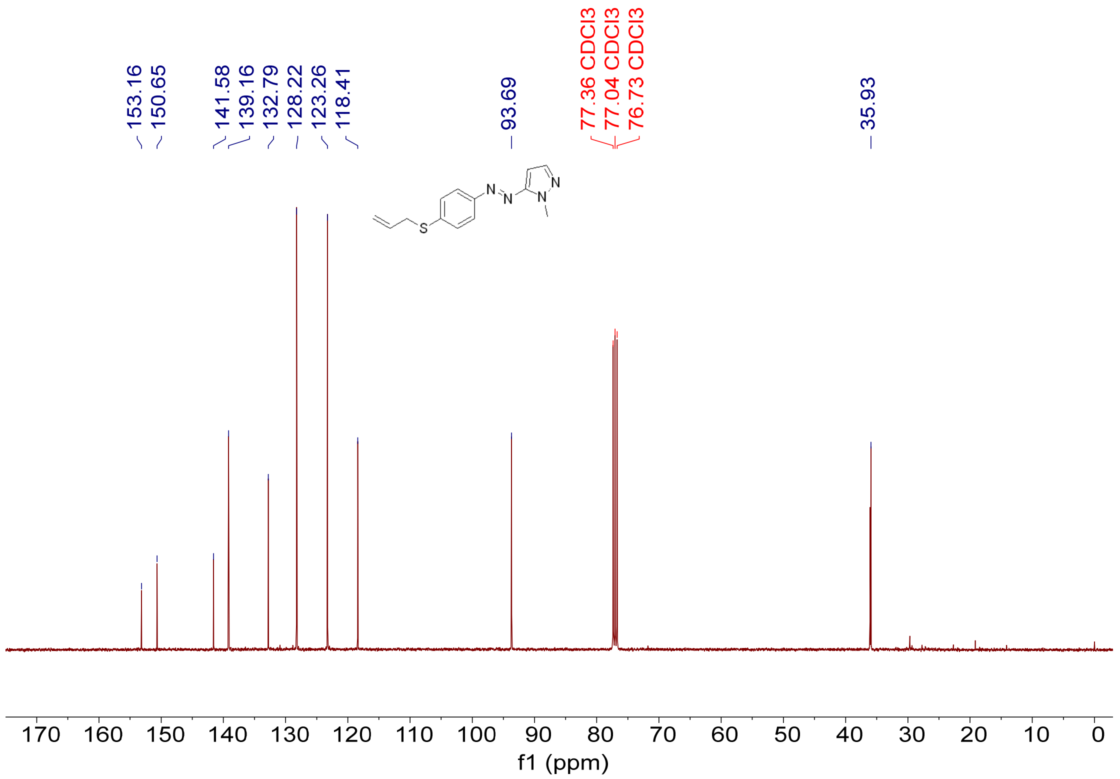
153.16
150.65
141.58
139.16
132.79
128.22
123.26
118.41
93.69
77.36 CDCl3
77.04 CDCl3
76.73 CDCl3
35.93
S
N
N
N
N
170 160 150 140 130 120 110 100 90 80 70 60 50 40 30 20 10 0
f1 (ppm)

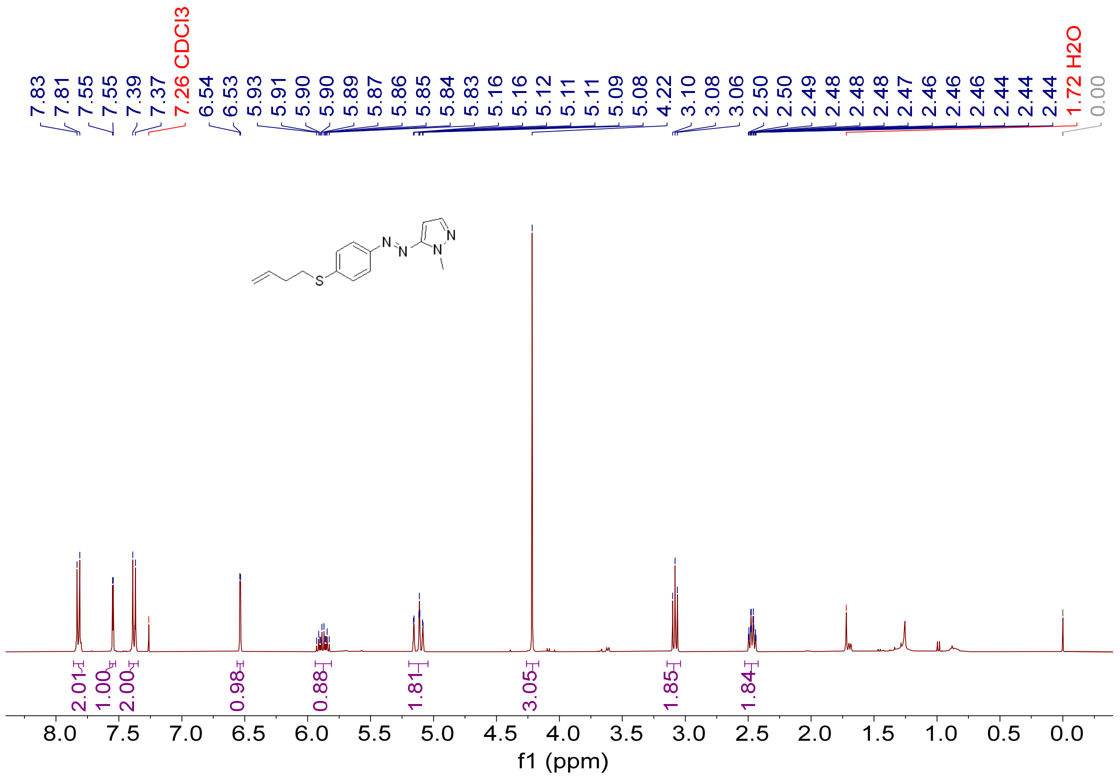
7.83
7.81
7.55
7.55
7.39
7.37
7.26 CDCl3
6.54
6.53
5.93
5.91
5.90
5.90
5.89
5.87
5.86
5.85
5.84
5.83
5.16
5.16
5.12
5.11
5.11
5.09
5.08
4.22
3.10
3.08
3.06
2.50
2.50
2.49
2.48
2.48
2.48
2.47
2.46
2.46
2.46
2.44
2.44
2.44
1.72 H2O
0.00
S
N
N
N
N
2.01
1.00
2.00
0.98
0.88
1.81
3.05
1.85
1.84
8.0 7.5 7.0 6.5 6.0 5.5 5.0 4.5 4.0 3.5 3.0 2.5 2.0 1.5 1.0 0.5 0.0
f1 (ppm)

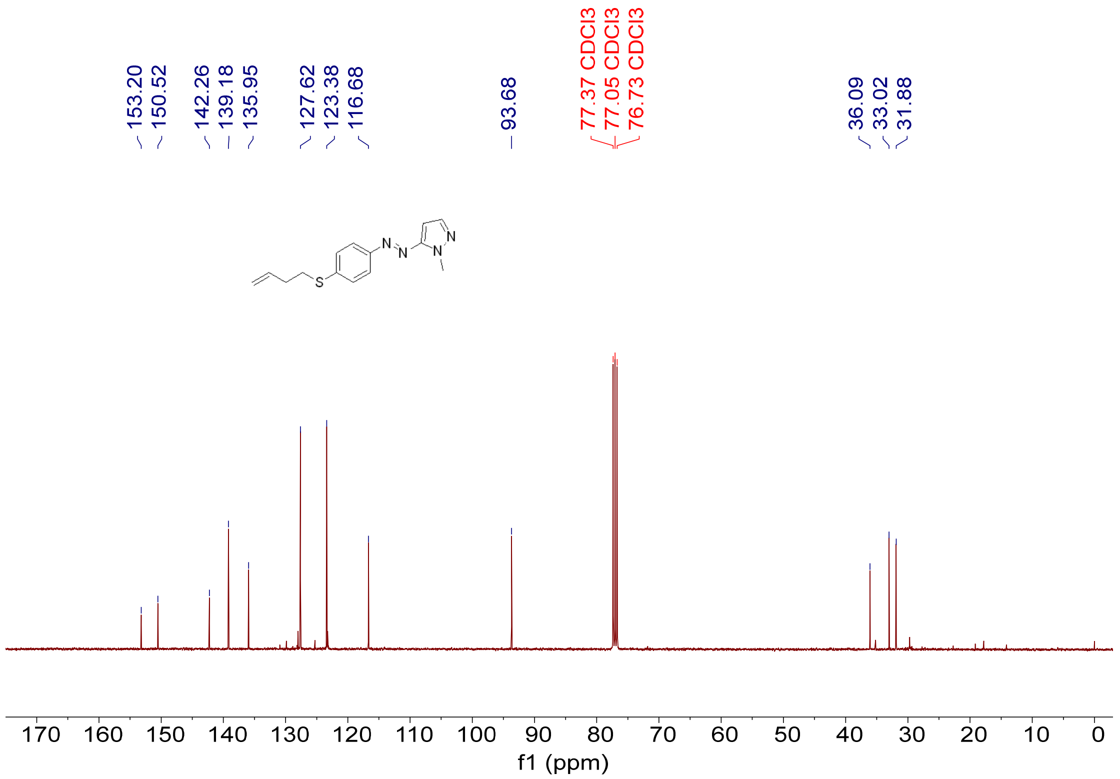
153.20
150.52
142.26
139.18
135.95
127.62
123.38
116.68
93.68
77.37 CDCl3
77.05 CDCl3
76.73 CDCl3
36.09
33.02
31.88
S
N
N
N
N
170 160 150 140 130 120 110 100 90 80 70 60 50 40 30 20 10 0
f1 (ppm)

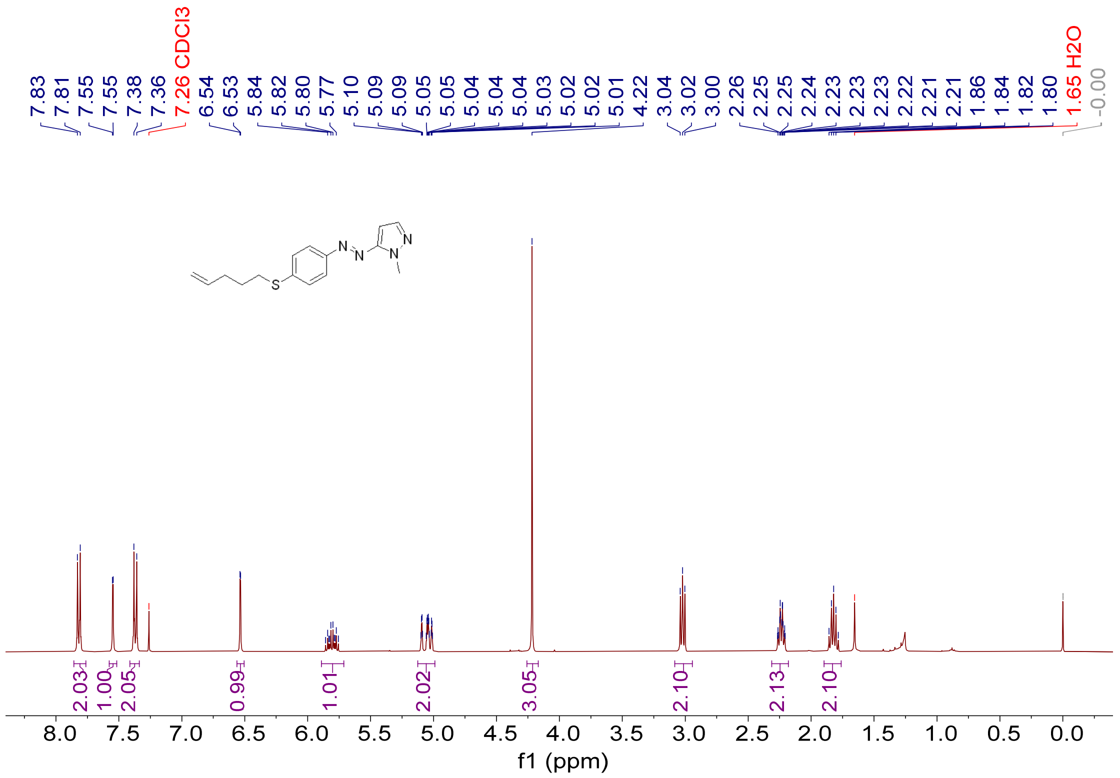
7.83
7.81
7.55
7.55
7.38
7.36
7.26 CDCl3
6.54
6.53
5.84
5.82
5.80
5.77
5.10
5.09
5.09
5.05
5.05
5.04
5.04
5.03
5.02
5.02
5.01
4.22
3.04
3.02
3.00
2.26
2.25
2.25
2.24
2.23
2.23
2.23
2.22
2.21
2.21
1.86
1.84
1.82
1.80
1.65 H2O
-0.00
S
N
N
N
N
2.03
1.00
2.05
0.99
1.01
2.02
3.05
2.10
2.13
2.10
8.0 7.5 7.0 6.5 6.0 5.5 5.0 4.5 4.0 3.5 3.0 2.5 2.0 1.5 1.0 0.5 0.0
f1 (ppm)

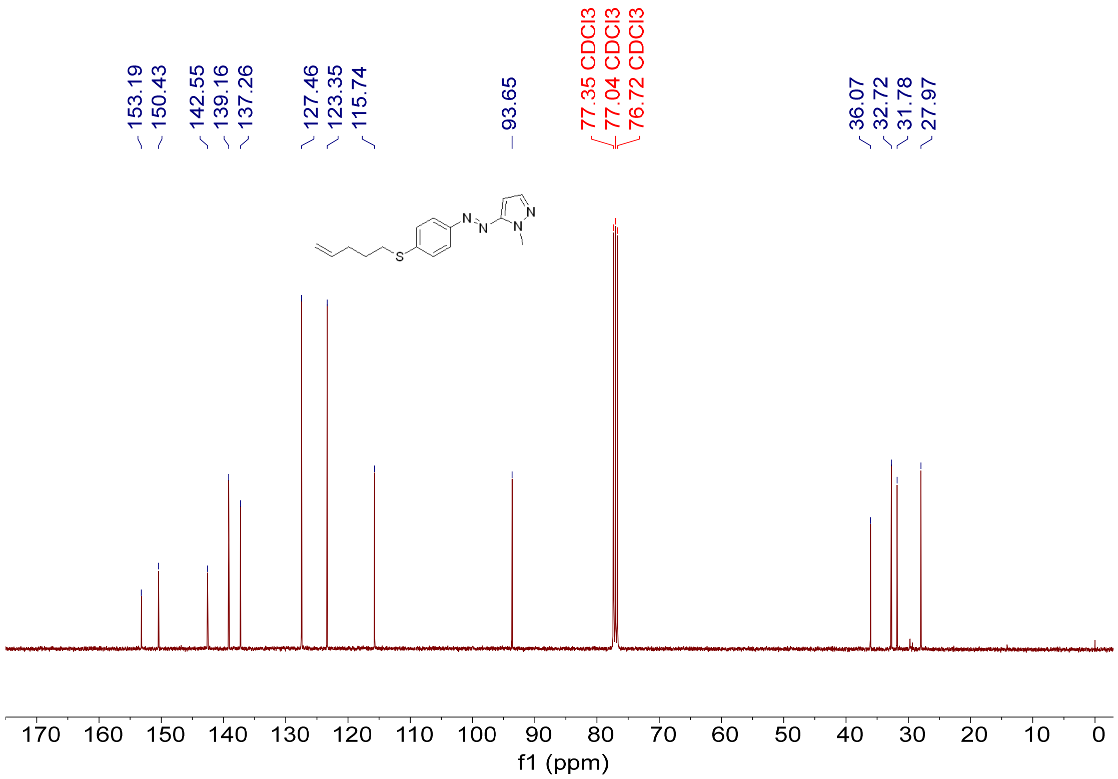
153.19
150.43
142.55
139.16
137.26
127.46
123.35
115.74
93.65
77.35 CDCl3
77.04 CDCl3
76.72 CDCl3
36.07
32.72
31.78
27.97
N
N
N
N
S
170 160 150 140 130 120 110 100 90 80 70 60 50 40 30 20 10 0
f1 (ppm)

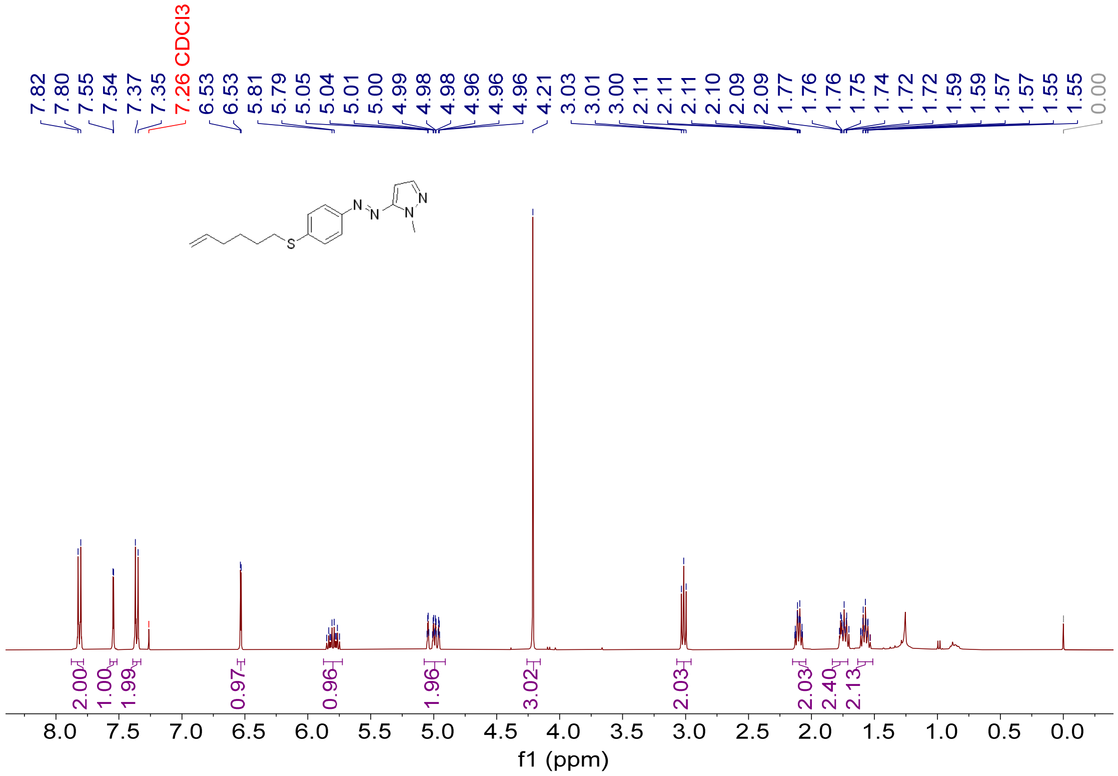
7.82
7.80
7.55
7.54
7.37
7.35
7.26 CDCl3
6.53
6.53
5.81
5.79
5.05
5.04
5.01
5.00
4.99
4.98
4.98
4.96
4.96
4.21
3.03
3.01
3.00
2.11
2.11
2.11
2.10
2.09
2.09
1.77
1.76
1.76
1.75
1.74
1.72
1.72
1.59
1.59
1.57
1.57
1.55
1.55
0.00
N
N
N
N
S
2.00
1.00
1.99
0.97
0.96
1.96
3.02
2.03
2.03
2.40
2.13
8.0 7.5 7.0 6.5 6.0 5.5 5.0 4.5 4.0 3.5 3.0 2.5 2.0 1.5 1.0 0.5 0.0
f1 (ppm)

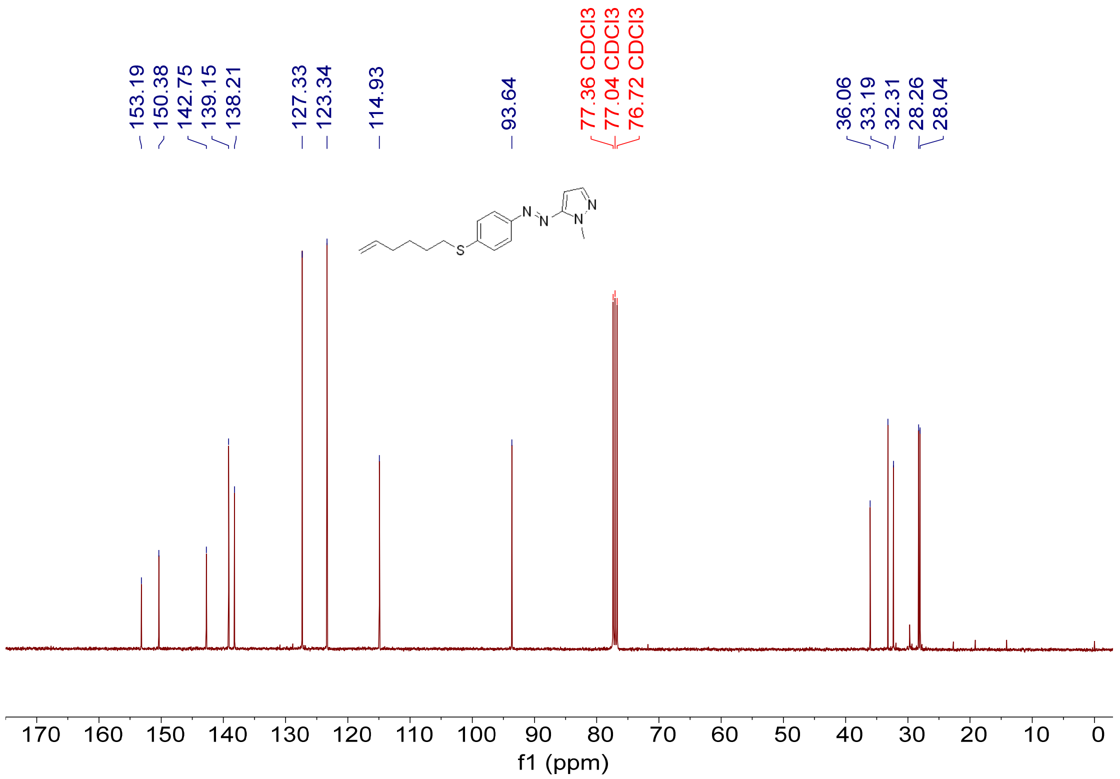
153.19
150.38
142.75
139.15
138.21
127.33
123.34
114.93
93.64
77.36 CDCl3
77.04 CDCl3
76.72 CDCl3
36.06
33.19
32.31
28.26
28.04
N
N
N
N
S
170 160 150 140 130 120 110 100 90 80 70 60 50 40 30 20 10 0
f1 (ppm)

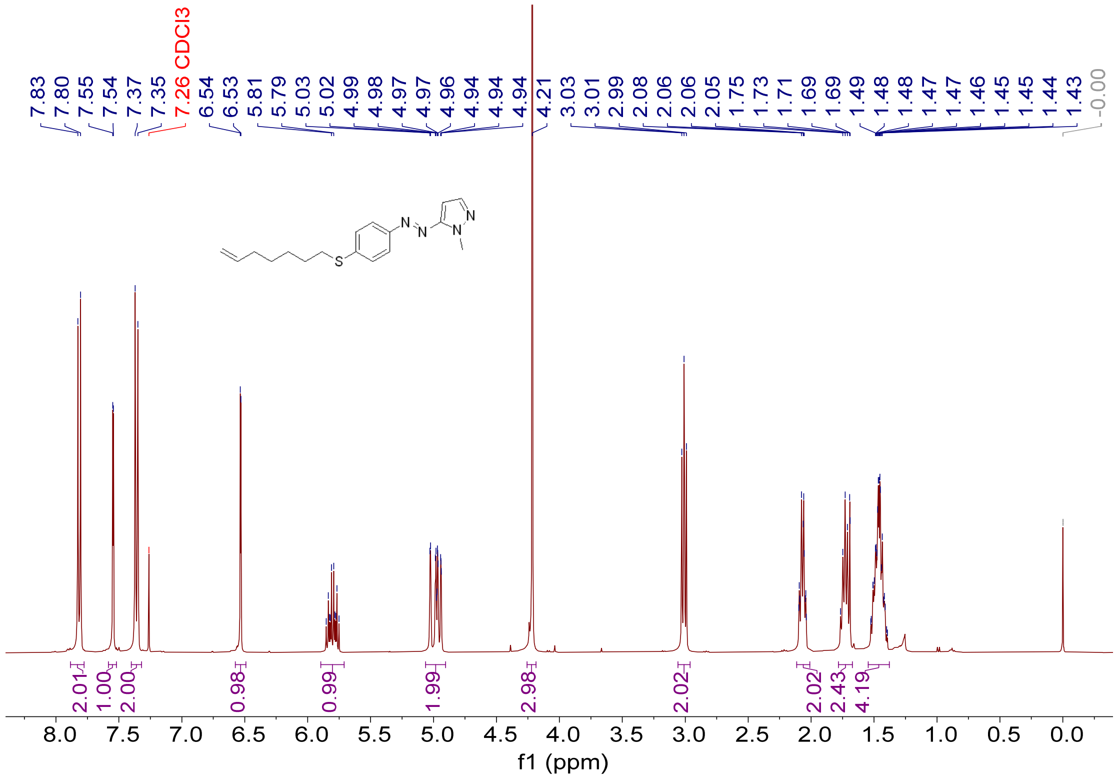
7.83
7.80
7.55
7.54
7.37
7.35
7.26 CDCl3
6.54
6.53
5.81
5.79
5.03
5.02
4.99
4.98
4.97
4.97
4.96
4.94
4.94
4.94
4.21
3.03
3.01
2.99
2.08
2.06
2.05
1.75
1.73
1.71
1.69
1.69
1.49
1.48
1.48
1.47
1.47
1.46
1.45
1.45
1.44
1.43
-0.00
N
N
N
N
S
2.01
1.00
2.00
0.98
0.99
1.99
2.98
2.02
2.02
2.43
4.19
8.0 7.5 7.0 6.5 6.0 5.5 5.0 4.5 4.0 3.5 3.0 2.5 2.0 1.5 1.0 0.5 0.0
f1 (ppm)

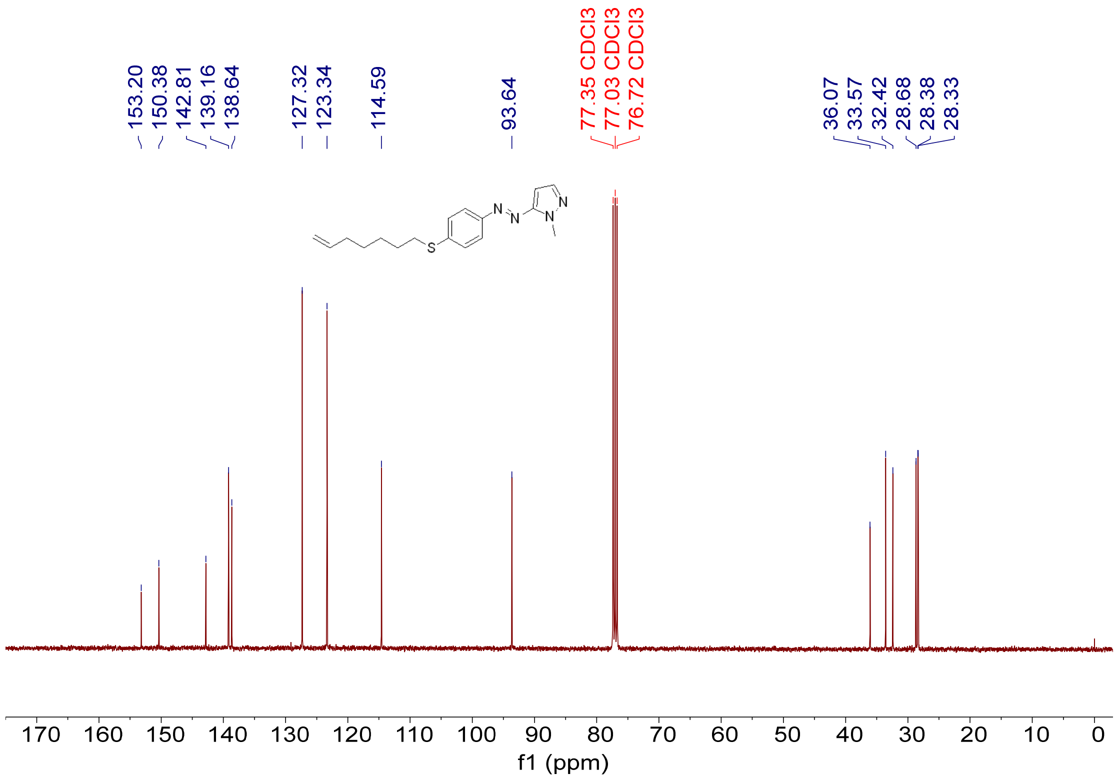
153.20
150.38
142.81
139.16
138.64
127.32
123.34
114.59
93.64
77.35 CDCl3
77.03 CDCl3
76.72 CDCl3
36.07
33.57
32.42
28.68
28.38
28.33
S
N
N
N
N
170 160 150 140 130 120 110 100 90 80 70 60 50 40 30 20 10 0
f1 (ppm)

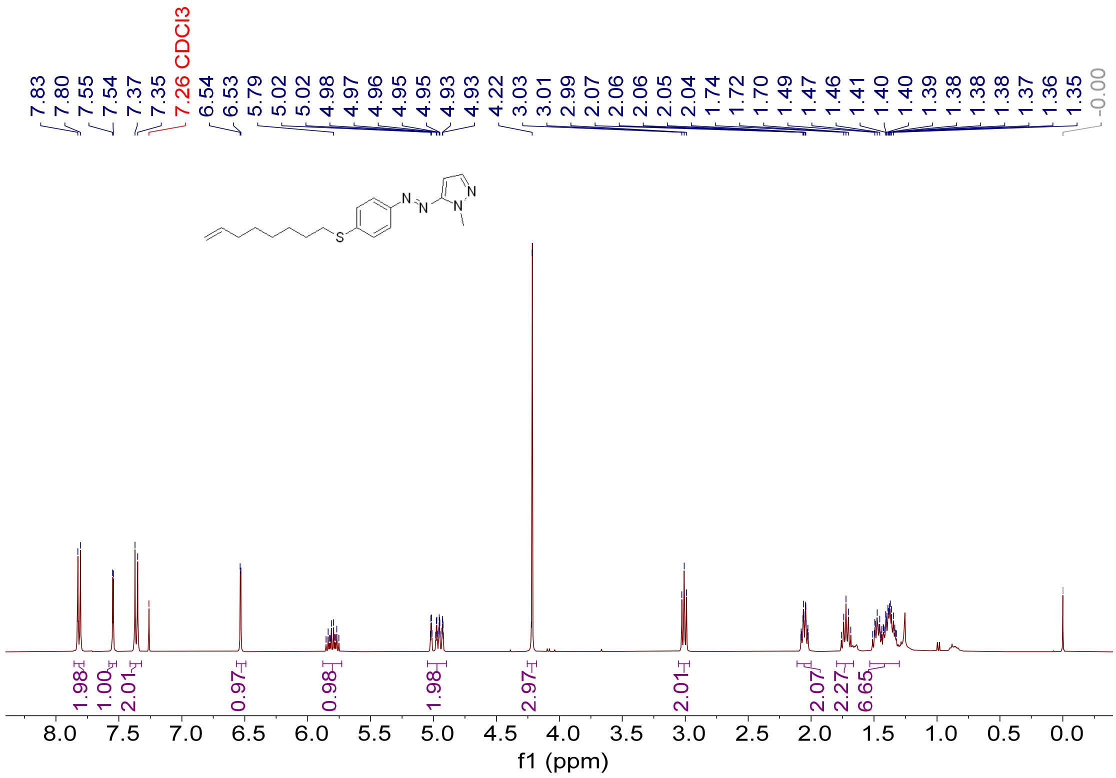
7.83
7.80
7.55
7.54
7.37
7.35
7.26 CDCl3
6.54
6.53
5.79
5.02
4.98
4.97
4.96
4.95
4.95
4.93
4.93
4.22
3.03
3.01
2.99
2.07
2.06
2.05
2.04
1.74
1.72
1.70
1.49
1.47
1.46
1.41
1.40
1.40
1.39
1.38
1.38
1.38
1.37
1.36
1.35
-0.00
S
N
N
N
N
1.98
1.00
2.01
0.97
0.98
1.98
2.97
2.01
2.07
2.27
6.65
8.0 7.5 7.0 6.5 6.0 5.5 5.0 4.5 4.0 3.5 3.0 2.5 2.0 1.5 1.0 0.5 0.0
f1 (ppm)

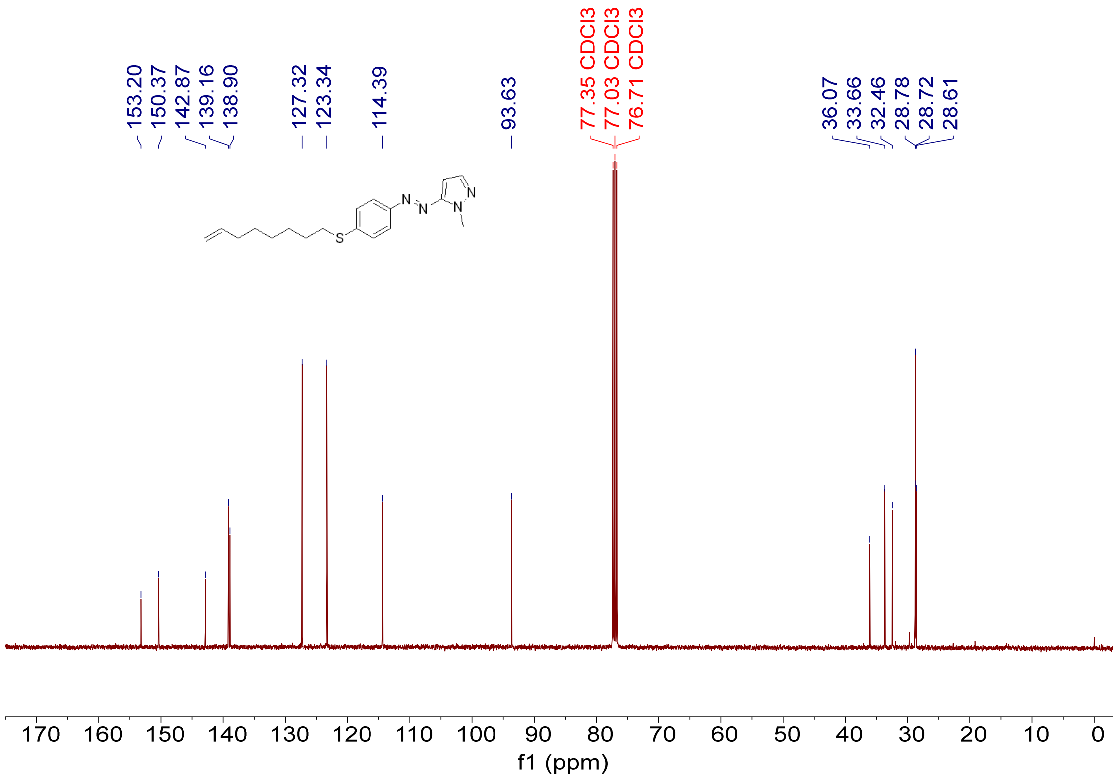
153.20
150.37
142.87
139.16
138.90
127.32
123.34
114.39
93.63
77.35 CDCl3
77.03 CDCl3
76.71 CDCl3
36.07
33.66
32.46
28.78
28.72
28.61
S
N
N
N
N
170 160 150 140 130 120 110 100 90 80 70 60 50 40 30 20 10 0
f1 (ppm)

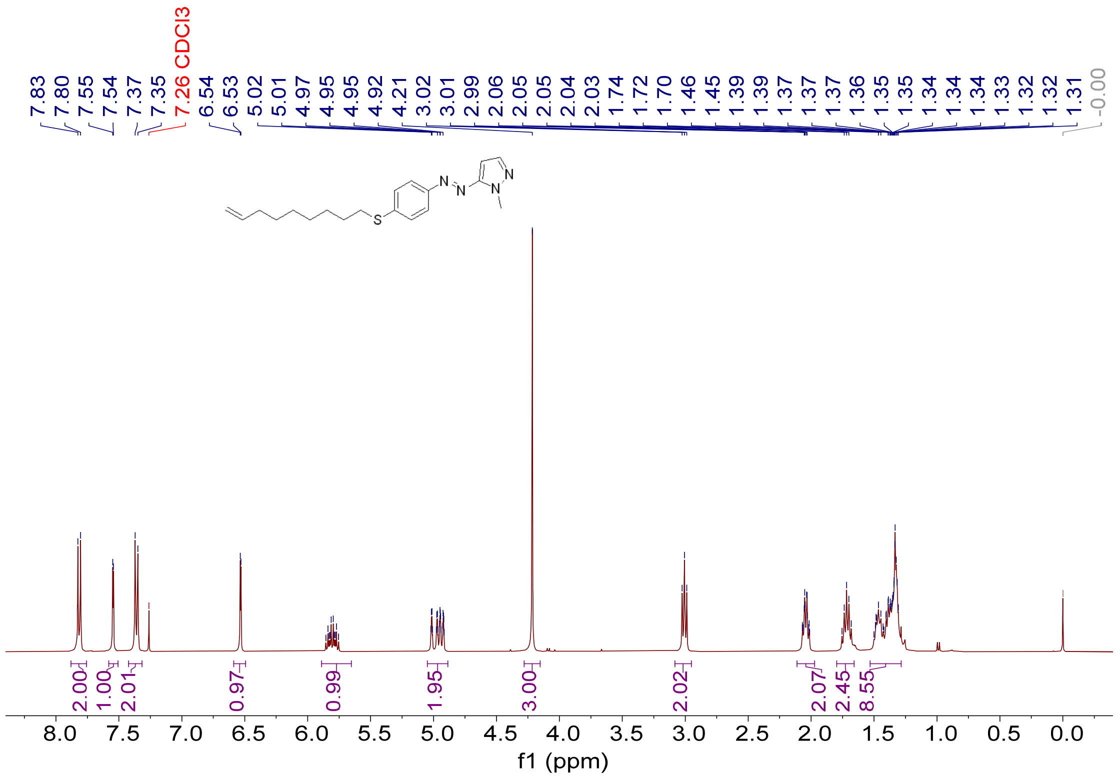
7.83
7.80
7.55
7.54
7.37
7.35
7.26 CDCl3
6.54
6.53
5.02
5.01
4.97
4.95
4.95
4.92
4.21
3.02
3.01
2.99
2.06
2.05
2.05
2.04
2.03
1.74
1.72
1.70
1.46
1.45
1.39
1.39
1.37
1.37
1.37
1.36
1.35
1.35
1.34
1.34
1.34
1.33
1.32
1.32
1.31
-0.00
S
N
N
N
N
2.00
1.00
2.01
0.97
0.99
1.95
3.00
2.02
2.07
2.45
8.55
8.0 7.5 7.0 6.5 6.0 5.5 5.0 4.5 4.0 3.5 3.0 2.5 2.0 1.5 1.0 0.5 0.0
f1 (ppm)

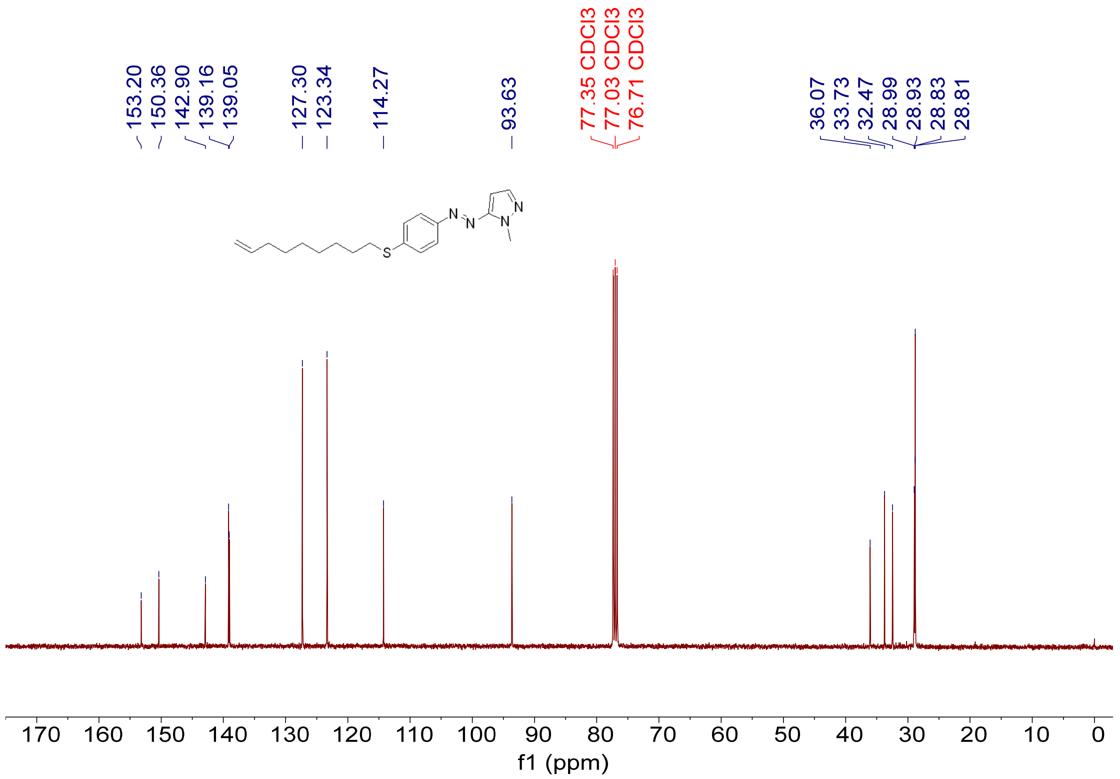
153.20
150.36
142.90
139.16
139.05
127.30
123.34
114.27
93.63
77.35 CDCl3
77.03 CDCl3
76.71 CDCl3
36.07
33.73
32.47
28.99
28.93
28.83
28.81
N
N
N
N
S
170 160 150 140 130 120 110 100 90 80 70 60 50 40 30 20 10 0
f1 (ppm)

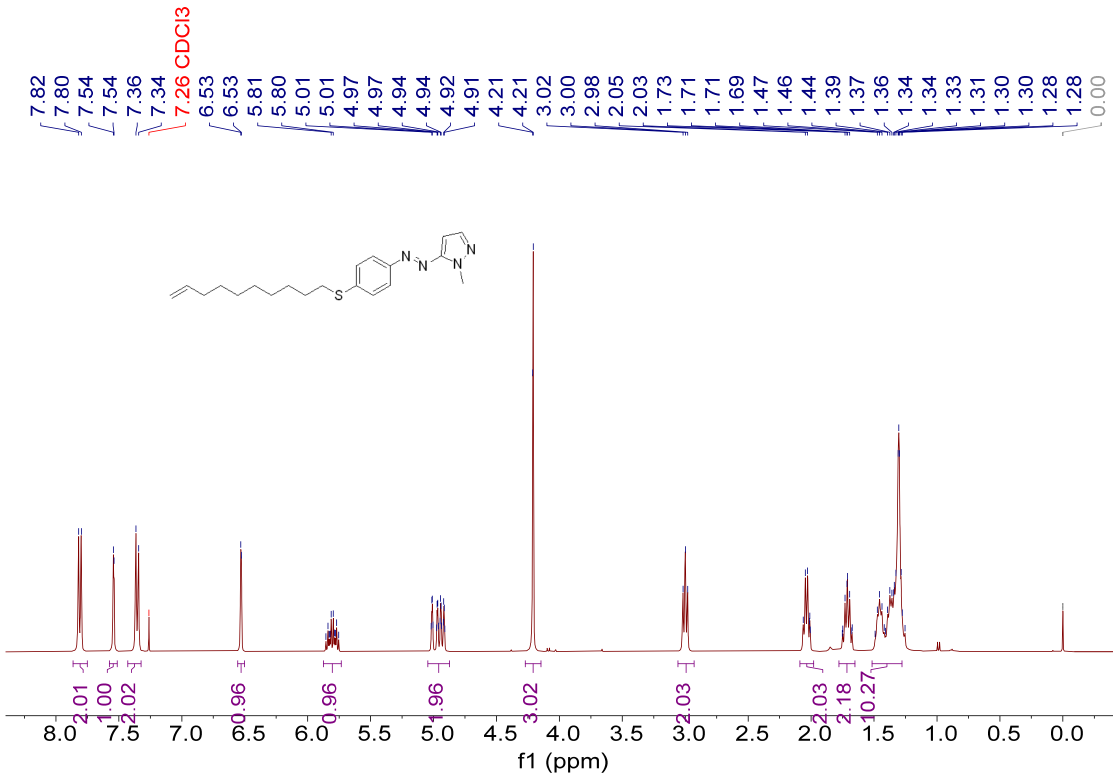
7.82
7.80
7.54
7.54
7.36
7.34
7.26 CDCl3
6.53
6.53
5.81
5.80
5.01
5.01
4.97
4.97
4.94
4.94
4.92
4.91
4.21
4.21
3.02
3.00
2.98
2.05
2.03
1.73
1.71
1.71
1.69
1.47
1.46
1.44
1.39
1.37
1.36
1.34
1.34
1.33
1.31
1.30
1.30
1.28
1.28
0.00
N
N
N
N
S
2.01
1.00
2.02
0.96
0.96
1.96
3.02
2.03
2.03
2.18
10.27
8.0 7.5 7.0 6.5 6.0 5.5 5.0 4.5 4.0 3.5 3.0 2.5 2.0 1.5 1.0 0.5 0.0
f1 (ppm)

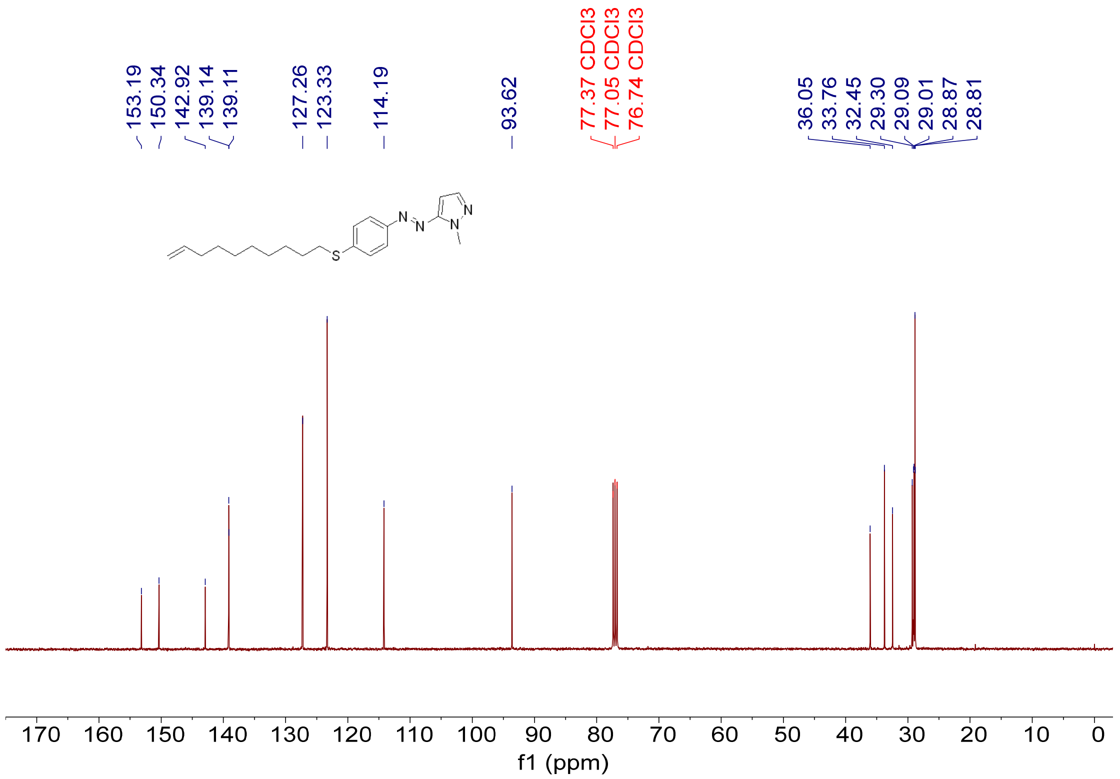
153.19
150.34
142.92
139.14
139.11
127.26
123.33
114.19
93.62
77.37 CDCl3
77.05 CDCl3
76.74 CDCl3
36.05
33.76
32.45
29.30
29.09
29.01
28.87
28.81
S
N
N
N
N
170 160 150 140 130 120 110 100 90 80 70 60 50 40 30 20 10 0
f1 (ppm)

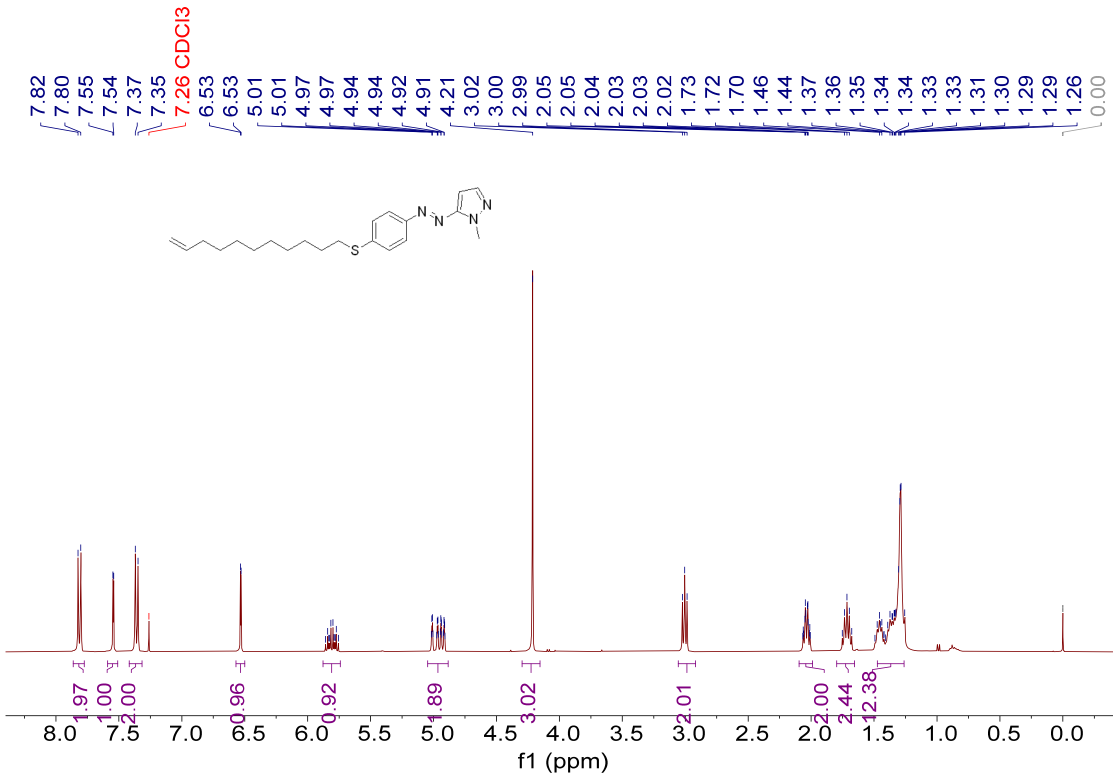
7.82
7.80
7.55
7.54
7.37
7.35
7.26 CDCl3
6.53
6.53
5.01
5.01
4.97
4.97
4.94
4.94
4.92
4.91
4.21
3.02
3.00
2.99
2.05
2.05
2.04
2.03
2.03
2.02
1.73
1.72
1.70
1.46
1.44
1.37
1.36
1.35
1.34
1.34
1.33
1.33
1.31
1.30
1.29
1.29
1.26
0.00
S
N
N
N
N
1.97
1.00
2.00
0.96
0.92
1.89
3.02
2.01
2.00
2.44
12.38
8.0 7.5 7.0 6.5 6.0 5.5 5.0 4.5 4.0 3.5 3.0 2.5 2.0 1.5 1.0 0.5 0.0
f1 (ppm)

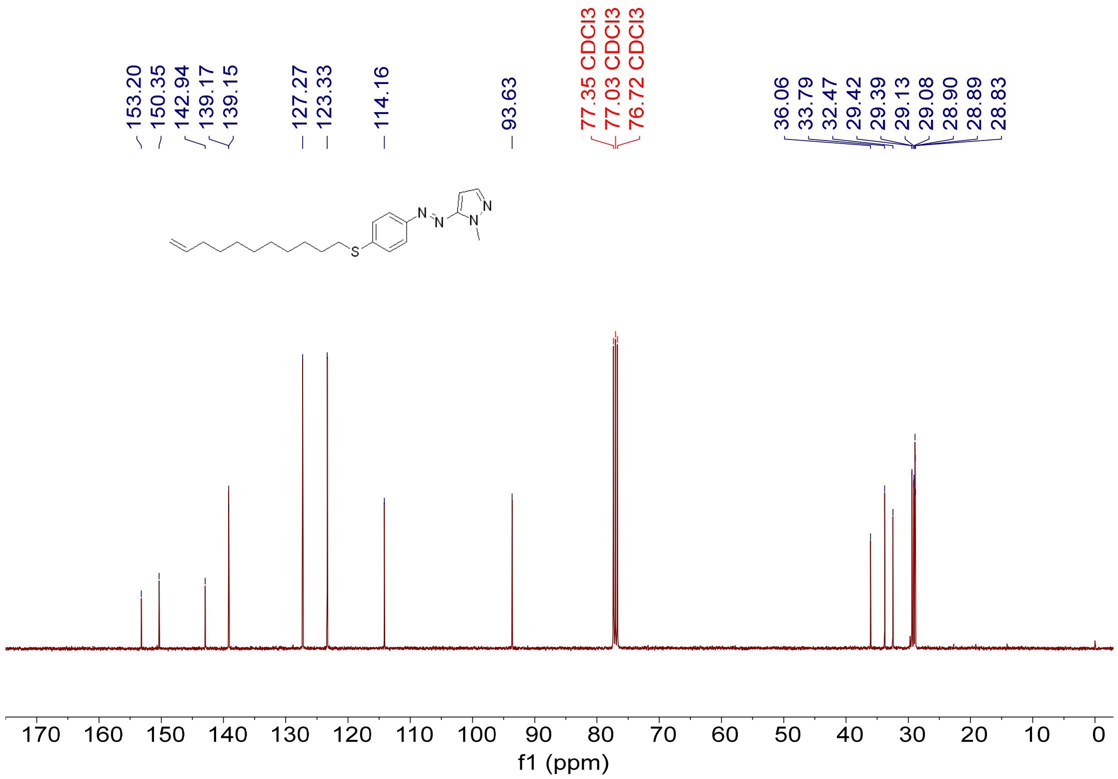

153.20
150.35
142.94
139.17
139.15
127.27
123.33
114.16
93.63
77.35 CDCl3
77.03 CDCl3
76.72 CDCl3
36.06
33.79
32.47
29.42
29.39
29.13
29.08
28.90
28.89
28.83
S
N
N
N
N
170 160 150 140 130 120 110 100 90 80 70 60 50 40 30 20 10 0
f1 (ppm)

11. Cartesian coordinates for optimized geometries

trans-O4 (1) in gas

C	3.44011629787398	-0.99959428218837	-0.00821424371304
C	2.45922163464509	-0.02313898299098	-0.00332721167472
C	3.17798568684919	1.19609744173695	-0.00169650903151
N	4.61789464298579	-0.35881977246607	-0.01197730085833
H	3.36006155907759	-2.07468013341801	-0.01152751179568
H	2.78708054547797	2.20072846651648	-0.00232450830838
N	4.47758089312214	0.97616751851064	-0.00545082075302
N	1.10496940462260	-0.30203545550912	-0.00508185739446
N	0.37033173245994	0.70715822604197	-0.00081891341531
C	-1.00849258854430	0.44360868704267	-0.00118568366740
C	-1.84615912757914	1.55899504179578	0.00327534334113
C	-1.57593367942934	-0.82845372634526	-0.00476995843147
C	-3.21594152641564	1.41281945988271	0.00439704948711
H	-1.39051822535634	2.54219362640679	0.00605642615051
C	-2.94900302512474	-0.98615539912713	-0.00360218341004
H	-0.92251880577568	-1.69150454345642	-0.00820852891640
C	-3.77862440345742	0.13721448294081	0.00113949386727
H	-3.87652142211588	2.27104708643448	0.00802061199020
H	-3.36867398443891	-1.98322780450762	-0.00623768339950
O	-5.12676565400034	0.08398466380732	0.00306380057962
C	5.93594949951682	-0.93164781694299	0.02272471040362
H	6.55458085974723	-0.46221077675512	-0.74158244225658
H	6.39721098951179	-0.77326971210241	0.99945817418189
H	5.86345857650154	-2.00008715132459	-0.17375531104169
C	-5.74068820824540	-1.18222785906195	0.00100621371278
H	-5.47168352589108	-1.75983994294883	0.89169556292953
H	-6.81349162669785	-0.99885841902506	0.00368694049179
H	-5.47537151931961	-1.75523392294667	-0.89375065906793

E[PWPB95-D3(BJ)/def2-QZVPP] = -720.393288 (Hartree)

Zero-point correction = 0.223042

Thermal correction to Energy = 0.237379

Thermal correction to Enthalpy= 0.238323

Thermal correction to Gibbs Free Energy= 0.182488

***trans*-O4 (2) in gas**

C	3.25085565189605	0.65647037648615	0.00125417869516
C	2.45375101327658	-0.48106889583051	-0.00716397638993
C	3.37096513635924	-1.55022415719245	-0.01680765817559
N	4.51800948660490	0.22313550760456	-0.00476009954786
H	2.98958169123488	1.70122902764177	0.00986821216152
H	3.15637460311499	-2.60805683961091	-0.02768944848494
N	4.61708101006331	-1.11399615799258	-0.01464609080587
N	1.07839535312791	-0.62366351953876	-0.00830250124509
N	0.44511210283378	0.45209938316008	-0.00816708694498
C	-0.95227155109235	0.32473529068307	-0.00648217096202
C	-1.67753804373317	1.51605033273416	-0.01628690442843
C	-1.64007495174336	-0.88641297867698	0.00578452537611
C	-3.05500349903042	1.50390823870139	-0.01456275712576
H	-1.12882465000363	2.45054142932723	-0.02563039568501
C	-3.02185249205401	-0.90961271980837	0.00822221351188
H	-1.07331587922803	-1.80871216643266	0.01393623021358
C	-3.73864374896740	0.28896791386784	-0.00196044041546
H	-3.62902075085847	2.42224364393644	-0.02252364516797
H	-3.53628914304786	-1.86124233503301	0.01853622050057
O	-5.08524006992455	0.36674912627032	-0.00005476506018
C	5.71692572073690	1.01739188829176	0.02334255590682
H	6.36539220348708	0.73044675858160	-0.80446008857360
H	5.44725929821471	2.06767918146454	-0.07115188301486
H	6.25038048044574	0.86130245541481	0.96231333839204
C	-5.81933970748740	-0.83399800275067	0.01124225225539
H	-5.60771582931963	-1.42447369423759	0.90887473401682
H	-6.86920879404744	-0.54730544080956	0.01009988266888
H	-5.60986164085835	-1.43978464625167	-0.87662643167120

E[PWPB95-D3(BJ)/def2-QZVPP] = -720.392954 (Hartree)

Zero-point correction = 0.223091

Thermal correction to Energy = 0.237410

Thermal correction to Enthalpy= 0.238354

Thermal correction to Gibbs Free Energy= 0.182493

***cis*-O4 (1) in gas**

C	-1.40213206264687	-0.63417502867343	-0.15941766228164
C	-1.99820508116426	0.65663658760500	-0.07334101200802
C	-3.35808525436521	0.38858709555973	-0.01338748862852
N	-2.32319817561753	-1.57635998336699	-0.14859792088106
H	-0.36384444133485	-0.90992125558315	-0.23529468563791
H	-4.19020513213825	1.07124408856323	0.05104914700315
N	-3.50067481330529	-0.93729771687003	-0.05419096763061
N	-1.59009113995501	1.99380575342873	-0.10505590258754
N	-0.40626329733333	2.36415310235786	-0.09676499046950
C	0.65569431984454	1.42607462459512	0.02912047608615
C	1.43250228240787	1.10827003583259	-1.07088065773482
C	0.98819798963361	0.90320034609733	1.27680529456390
C	2.50660524963893	0.23530774664732	-0.94932676437140
H	1.18813883745090	1.53658141294744	-2.03592682588586
C	2.06217886298278	0.04836205297299	1.40759949349211
H	0.39300552110823	1.16826050897022	2.14280210850893
C	2.82628608207219	-0.29960628680077	0.29378259243151
H	3.08941085331399	-0.00837394681984	-1.82716830500139
H	2.33261215048829	-0.36589742071132	2.37133167190134
O	3.85812577523344	-1.14494294511735	0.52057280156408
C	-4.71771558685981	-1.70175419873892	0.00192403270156
H	-4.77644099160817	-2.36173288122076	-0.86357509059925
H	-4.74169364496711	-2.30462965847727	0.91068395179075
H	-5.56442541898446	-1.01771699760605	-0.00301756495189
C	4.65693157504177	-1.51080118037844	-0.57676247485643
H	5.14769211065993	-0.63952884179711	-1.02427727237755
H	5.41491860202961	-2.18918145455421	-0.18950976052888
H	4.07127382837407	-2.02684155886191	-1.34552522361121

E[PWPB95-D3(BJ)/def2-QZVPP] = -720.374068 (Hartree)

Zero-point correction = 0.222940

Thermal correction to Energy = 0.237191

Thermal correction to Enthalpy= 0.238135

Thermal correction to Gibbs Free Energy= 0.182001

***cis*-O4 (2) in gas**

C	1.52470178780345	0.53785844726272	0.01379027350850
C	2.20438590993470	-0.67812022359248	-0.10027191777499
C	3.56515809379529	-0.30117017178979	-0.13773679328061
N	2.46623504149797	1.48784086885569	0.03557843011211
H	0.47998810598931	0.78776167470688	0.07885570871000
H	4.41766498551786	-0.95800837218399	-0.22214044644542
N	3.71529176139271	1.00331355615361	-0.05472371042472
N	1.86327181790370	-2.02919843378268	-0.18255979036019
N	0.70057626734374	-2.46278533245492	-0.15020483569329
C	-0.40129149805818	-1.57397826300255	-0.01171988511917
C	-1.06362012785268	-1.10323564154483	-1.13252535847095
C	-0.87315719759375	-1.23696243658156	1.25416384266572
C	-2.16531006010348	-0.26633155684440	-1.00329200338280
H	-0.70918451344215	-1.38382550576616	-2.11753352205945
C	-1.96700364907734	-0.40651215481287	1.38994095021276
H	-0.36809329564676	-1.62439595801886	2.13128020287276
C	-2.61937888430739	0.09124804128047	0.26209282581566
H	-2.66268692053078	0.09056849090332	-1.89496977583333
H	-2.34339211344622	-0.13432814970139	2.36871533454534
O	-3.68350289700005	0.89483218375502	0.49522672984924
C	2.26865987199603	2.90931847952728	0.13985898758851
H	1.20197409348969	3.11237148532021	0.21491031711815
H	2.77651222931863	3.28875188928908	1.02693301055992
H	2.67528274904292	3.40600484255825	-0.74165983246836
C	-4.39213947834274	1.37973564609050	-0.61818823331584
H	-4.81113364981653	0.56160972388318	-1.21402958952487
H	-5.20389036346597	1.98709363898623	-0.22227124885673
H	-3.75743106634198	2.00138823150405	-1.25952467054794

E[PWPB95-D3(BJ)/def2-QZVPP] = -720.374386 (Hartree)
Zero-point correction = 0.222987
Thermal correction to Energy = 0.237194
Thermal correction to Enthalpy= 0.238139
Thermal correction to Gibbs Free Energy= 0.182400

***trans*-S3 (1) in gas**

C	3.35515504810443	0.21142852274907	-0.00186743504966
C	2.51708696147249	-0.90729340564040	0.01123173187917
C	1.14549659406319	-0.75490655271075	0.01433016087667
C	0.57889162590537	0.51913872720635	0.00471560022449
C	1.41389805838119	1.63480016638693	-0.00732601098192
C	2.78466473387192	1.48727634250268	-0.01089817726015
H	2.93475890109897	-1.90532836972756	0.01902184886468
H	0.49158234033681	-1.61769595551909	0.02398941650777
H	0.95900901808838	2.61836140146371	-0.01448696167929
N	-0.80075806383345	0.77793827639879	0.00537280657506
N	-1.52605093253862	-0.23659873429138	0.00968661243937
C	5.44859338602680	-1.62033270186834	-0.00342049524657
H	5.05880790094421	-2.10930534121336	-0.89702027032048
H	6.53558475495029	-1.70219314014688	-0.00395056899666
H	5.05993038655578	-2.10469839173492	0.89317501258534
S	5.10040006966213	0.13616312002670	-0.00788344003172
H	3.42607707714071	2.36147496398439	-0.02095387221925
C	-2.88364137126148	0.04532866230905	0.00820676564624
C	-3.57440853365266	1.27783262762494	0.00957013119476
N	-4.93208222463383	-0.44188596313930	0.01132366918312
C	-4.89371551452675	0.91391341963212	0.01002734083696
H	-3.15056554012298	2.26653065895466	0.01498336924391
H	-5.79915176874194	1.50036108892750	0.01223190389917
N	-3.72587953508857	-0.98608122028449	0.00755294223380
C	-6.09675528515497	-1.28488510906632	-0.02887553941491
H	-6.02171604287657	-2.04743893348014	0.74589267000181
H	-6.98058608179064	-0.67381647278571	0.14693152227421
H	-6.18206405238017	-1.77366064655826	-1.00126183326591

E[PWPB95-D3(BJ)/def2-QZVPP] = -1043.354911 (Hartree)
Zero-point correction = 0.219031
Thermal correction to Energy = 0.234046
Thermal correction to Enthalpy= 0.234990
Thermal correction to Gibbs Free Energy= 0.177527

***trans*-S3 (2) in gas**

C	3.31361122986157	0.32351706050331	0.00000319618352
C	2.59961934148255	-0.87821049389342	0.00000910191340
C	1.21971345573036	-0.87180918244488	0.00001064642973
C	0.52089154372210	0.33457440123966	0.00000461155336
C	1.23303967166404	1.53254307926313	0.00000053081238
C	2.61173477562259	1.53150600165798	-0.00000087865046
H	3.12053923538976	-1.82644655272866	0.00001337127874
H	0.66137691295361	-1.79931364826744	0.00001428669311
H	0.67345603162747	2.46044989263321	-0.00000445908085
N	-0.87772300679234	0.45297662062455	-0.00000015483273
N	-1.49408465861052	-0.62976976428869	0.00000528830075
C	5.58291794124143	-1.27994995922241	-0.00000657717057
H	5.24385619159668	-1.80285955248746	-0.89503820345709
H	6.67260978280709	-1.25268395653339	-0.00000796941415
H	5.24385853571661	-1.80286856890202	0.89502066856300
S	5.05795371913554	0.43186447939125	0.00000244985022
H	3.15692953237246	2.46875494503817	-0.00000577896325
C	-2.88097868338079	-0.52736744666182	0.00000358925383
C	-3.72893383867546	-1.65193960321676	-0.00000082462138
N	-4.84552412066436	0.22432241923323	-0.00000136821043
C	-4.99334234790359	-1.12081628966439	-0.00000463777466
H	-3.43753273186363	-2.68854937572557	-0.00000329482188
H	-5.96850434992861	-1.58073248130533	-0.00001001935819
N	-3.57918954744128	0.60886270905261	0.00000462966828
C	-5.88767547135098	1.21621970756456	0.00000331843450
H	-5.80375853076655	1.84404786088594	-0.88766949678698
H	-6.85279089064946	0.71240395468870	-0.00001662734170
H	-5.80378018289629	1.84401828356594	0.88769932154952

E[PWPB95-D3(BJ)/def2-QZVPP] = -1043.350310 (Hartree)

Zero-point correction = 0.218841

Thermal correction to Energy = 0.233942

Thermal correction to Enthalpy= 0.234887

Thermal correction to Gibbs Free Energy= 0.177662

cis-S3 (1) in gas

C	-2.51100610804975	0.14434083406636	0.30414287804416
C	-2.38353202402056	-0.81966465285556	-0.69228451124210
C	-1.26277064610851	-1.63429630696455	-0.73858974221936
C	-0.22949563412441	-1.46261013589322	0.16954024921305
C	-0.36211914468132	-0.51532454544889	1.18438137323822
C	-1.49128129113122	0.27067727345377	1.25349551831490
H	-3.16082078024806	-0.95828920650527	-1.43177845050363
H	-1.17509181237835	-2.40220050091159	-1.49844787122653
H	0.42552091882502	-0.40399214559612	1.91982864323018
N	0.86864818213002	-2.35824938802218	0.12734023056557
N	2.04418462988195	-1.97114679008308	0.07684227609000
C	-4.94824798475817	0.74727644795412	-0.87819221366767
H	-5.27513115392623	-0.29002676038160	-0.79358229852853
H	-5.82096656262463	1.39652591434376	-0.80781622633169
H	-4.46495883144279	0.91193546248439	-1.84236272294871
S	-3.88400903239500	1.21665577216922	0.48208632492718
H	-1.59138645595274	0.99882614951612	2.05103824188099
C	2.39809438023266	-0.62631673077793	-0.13809779008647
C	1.78004563186769	0.43099859034644	-0.84828871034573
N	3.78458427875761	0.96513586151772	-0.14743608327633
C	2.71387582744499	1.43310344622643	-0.82970124746981
H	0.80818121108409	0.45688429928601	-1.30818143155361
H	2.69881221474936	2.42792234190572	-1.24633555979371
N	3.62143388175443	-0.28297271088078	0.26036568664951
C	4.99049307289220	1.67550975992422	0.18641848954930
H	5.84674617345005	1.02420250913566	0.01807268512253
H	5.07038532962239	2.55586730342561	-0.44942063750403
H	4.97845372914926	1.98473990856521	1.23355389987232

E[PWPB95-D3(BJ)/def2-QZVPP] = -1043.333479 (Hartree)
Zero-point correction = 0.218797
Thermal correction to Energy = 0.233732
Thermal correction to Enthalpy= 0.234676
Thermal correction to Gibbs Free Energy= 0.177225

***cis*-S3 (2) in gas**

C	-2.31629970482893	-0.01997938639750	0.32922020931659
C	-2.16820487342250	-0.80733116366364	-0.80870436514823
C	-1.02668016861876	-1.57197108720164	-0.98436561837427
C	0.00756064842920	-1.51571507660749	-0.06387591064714
C	-0.14538147685694	-0.74715514273505	1.08972991385080
C	-1.29679600382807	-0.01881178130919	1.28753979682218
H	-2.94592161594399	-0.84556259310986	-1.55942391392945
H	-0.91969174194385	-2.20749232950351	-1.85543002118241
H	0.64006700811950	-0.73163715257170	1.83573223099033
N	1.11400324077199	-2.38077483386223	-0.25728065811564
N	2.28865668461156	-2.01342000534853	-0.12445730515046
C	-4.78642828305625	0.67719193125266	-0.73004661193448
H	-5.08573463198639	-0.36990592398491	-0.79270291135505
H	-5.67375781387504	1.28628482563375	-0.55700615346067
H	-4.31997102584773	0.99268239004472	-1.66432818105226
S	-3.71678108974329	0.97264708516260	0.67408673369228
H	-1.41589553666398	0.56481605174675	2.19404046867086
C	2.64879194993354	-0.65923016056609	0.02120964373725
C	3.72487946509048	-0.20150297251895	0.80287263325026
N	2.79717981181321	1.40797306768941	-0.35377321455328
C	3.77265147396635	1.14640166762996	0.54381070563512
H	4.35328326952220	-0.77875241989903	1.45965327638307
H	4.42058337230137	1.92444240107055	0.91473801130117
N	2.10426572707985	0.32486947212884	-0.68663351294706
C	2.51272573810494	2.65890694844424	-1.00519838124867
H	1.43886164200276	2.84065398393712	-0.98327738946426
H	3.02902559257616	3.45802968399531	-0.47573287088440
H	2.84939256229260	2.63477283054341	-2.04344713420218

E[PWPB95-D3(BJ)/def2-QZVPP] = -1043.332727 (Hartree)
Zero-point correction = 0.218627
Thermal correction to Energy = 0.233609
Thermal correction to Enthalpy= 0.234553
Thermal correction to Gibbs Free Energy= 0.177148

***trans*-S4 (1) in gas**

C	3.38295695677506	0.21931013209550	-0.00107619377174
C	2.55843434061106	-0.90878814546315	0.00598537960104
C	1.18483750972143	-0.77254403439258	0.00788778709659
C	0.60123083282460	0.49360278345834	0.00284846732773
C	1.42420273078630	1.61851289382721	-0.00359121543496
C	2.79673331406685	1.48750065372036	-0.00568059764902
H	2.98776163751447	-1.90189594585647	0.00996235793831
H	0.54331953773194	-1.64438780462851	0.01312936961163
H	0.95785348988411	2.59668575098393	-0.00735070542183
N	-0.78084155122183	0.73920565665985	0.00312086084225
N	-1.49932683516373	-0.28254752661406	0.00580831423781
C	5.49750072133310	-1.58775396774221	-0.00111622304997
H	5.11064494271161	-2.08071905868924	-0.89380019515540
H	6.58524801506277	-1.65841688600621	-0.00414028998460
H	5.11592402753383	-2.07603159336121	0.89639466167220
S	5.13023898099312	0.16506836667399	-0.00475022565257
H	3.42701803583463	2.36980963681318	-0.01113421081525
C	-2.85691515333385	-0.02702505219587	0.00419333779082
C	-3.82087473586520	-1.02092334210682	0.00517462079247
C	-3.59708597983808	1.17959008886899	0.00462336236582
N	-5.00900047644920	-0.40109814272292	0.00774471066353
H	-3.72159044268697	-2.09438866707603	0.00571624973568
H	-3.22422932156513	2.19105700124056	0.00731036373839
N	-4.89222205568232	0.93667498306869	0.00571049157035
C	-6.31735438782349	-0.99640002613038	-0.01938913087112
H	-6.21911328215226	-2.07220949106913	0.11544133607174
H	-6.92365929174263	-0.58247611801704	0.78614788086150
H	-6.80786290986017	-0.79364739533876	-0.97299579411141

E[PWPB95-D3(BJ)/def2-QZVPP] = -1043.353267 (Hartree)

Zero-point correction = 0.219119

Thermal correction to Energy = 0.234178

Thermal correction to Enthalpy= 0.235122

Thermal correction to Gibbs Free Energy= 0.177528

***trans*-S4 (2) in gas**

C	-3.34436858083972	-0.32018849019304	0.00001142471103
C	-2.62292111790980	0.87668239789002	0.00001654085137
C	-1.24293019521271	0.86306827653454	0.00001344045286
C	-0.54995810224606	-0.34658106111088	0.00000375486149
C	-1.26978366124804	-1.53994001808541	-0.00000225121967
C	-2.64852353180856	-1.53144474360116	0.00000174897650
H	-3.13760385149011	1.82827040614641	0.00002438286818
H	-0.68069598898347	1.78809677694553	0.00001819143092
H	-0.71864401601801	-2.47303020742435	-0.00000936127423
N	0.84787667813068	-0.46712594526999	0.00000117394506
N	1.47261346954684	0.61452095918453	0.00000700813821
C	-5.60256635007642	1.29715399160095	-0.00001164689960
H	-5.25968871527716	1.81753924572391	-0.89506520197212
H	-6.69239214405188	1.27773486772087	0.00000762294426
H	-5.25965731128383	1.81757888351553	0.89500685359431
S	-5.08963268733837	-0.41829929664832	0.00001756188990
H	-3.19820986316730	-2.46606890414870	-0.00000198520079
C	2.84779261030248	0.48466953559629	0.00000472525494
C	3.65556741330211	-0.64585240720749	-0.00000105234254
C	3.75516364733101	1.56256864039686	0.00000698987429
N	4.91789839208564	-0.20074891650567	-0.00000357988176
H	3.40369934414628	-1.69292940100241	-0.00000455340783
H	3.53054609653366	2.61836987447176	0.00001003432692
N	5.00479189870643	1.13780500098025	0.00000186157213
C	6.12466168637332	-0.98368111333984	0.00000372705922
H	5.86013430210894	-2.03943371395594	-0.00005889699686
H	6.71544027935628	-0.75513805277120	-0.88767476702314
H	6.71537813902779	-0.75522878544304	0.88774776346693

E[PWPB95-D3(BJ)/def2-QZVPP] = -1043.352910 (Hartree)
Zero-point correction = 0.219186
Thermal correction to Energy = 0.234220
Thermal correction to Enthalpy= 0.235165
Thermal correction to Gibbs Free Energy= 0.177558

***cis*-S4 (1) in gas**

C	-2.51631902479956	0.03099672389725	-0.23999038932263
C	-2.06308582050010	0.29191171458918	1.04974001657361
C	-0.93385130082617	1.07150668271361	1.25386354418657
C	-0.23181506802243	1.58302880256639	0.17431965113266
C	-0.69515607217626	1.35153524453581	-1.11721017769546
C	-1.82113987754952	0.57979201385968	-1.32077115295839
H	-2.58439483607112	-0.10634646390813	1.91001503047892
H	-0.58535026326814	1.27197374488250	2.26034503822132
H	-0.15859112355985	1.77003093694620	-1.96064970461642
N	0.90005422990591	2.41543373213244	0.38617630579907
N	2.04981681426905	1.95080236255758	0.35326191268524
C	-4.50317370346256	-1.47917486204642	0.98139402721231
H	-4.80373048132822	-0.63851318303735	1.60837157666493
H	-5.37767605294759	-2.09970485799931	0.78542143010734
H	-3.75229423000969	-2.08249197617732	1.49365375587419
S	-3.92870367982327	-0.93533047417088	-0.62419476569672
H	-2.16981664199480	0.39445901311726	-2.33094233475349
C	2.34660272972818	0.60096578823450	0.14488295722398
C	3.68195500270232	0.22569760570006	0.09859764470392
C	1.64368478869927	-0.62670831415820	-0.02548787790965
N	3.71387972657795	-1.09465943877448	-0.08534667107273
H	4.56877804923801	0.83258067451728	0.18703928623681
H	0.58437866657041	-0.81837151833952	-0.05249944921444
N	2.48474268361186	-1.63167784072571	-0.15991307967796
C	4.86609924863781	-1.94828734078586	-0.19873886782877
H	5.76499001641663	-1.33858115594185	-0.12811874367745
H	4.86201781368209	-2.68655946271418	0.60377055661787
H	4.85050645629980	-2.46628578147055	-1.15796700929462

E[PWPB95-D3(BJ)/def2-QZVPP] = -1043.334492 (Hartree)

Zero-point correction = 0.219073

Thermal correction to Energy = 0.234039

Thermal correction to Enthalpy= 0.234983

Thermal correction to Gibbs Free Energy= 0.177019

***cis*-S4 (2) in gas**

C	-2.31596649342530	-0.19206613308797	0.25743918664816
C	-1.82191689710610	-0.51026347190429	-1.00460807339571
C	-0.66182532836834	-1.25904935998986	-1.13686270055224
C	0.02971580521628	-1.68524880654093	-0.01340285784474
C	-0.47443929836976	-1.39817335950192	1.25150228313771
C	-1.63021246101642	-0.65450043531783	1.38409256906362
H	-2.33611339968271	-0.18216487960939	-1.89820056931339
H	-0.28061650845934	-1.50364360640294	-2.12152468878129
H	0.05280261372755	-1.75317178978482	2.12926885331708
N	1.19862184053614	-2.47885315154697	-0.15181240474998
N	2.32250287651048	-1.95269526728048	-0.19270241717197
C	-4.40692366804911	1.03888890281128	-1.09640867116178
H	-4.63653160637787	0.10223586731368	-1.60620287848498
H	-5.33168650490327	1.59886673846240	-0.95677329184520
H	-3.72274662465231	1.63862053444953	-1.69826578893339
S	-3.76687309299912	0.74891437378305	0.55021281390100
H	-2.01499705916783	-0.43172769700292	2.37345522237683
C	2.55186121292627	-0.57854557777690	-0.11364535525491
C	1.77502273478955	0.57629755124842	0.02051178952783
C	3.87564955429082	-0.08893275392319	-0.17223466919160
N	2.63416671049422	1.60078476286435	0.03246728992694
H	0.71480429954846	0.73891750077343	0.10678048077591
H	4.77841738685923	-0.67160596465234	-0.27609786056502
N	3.91760606854308	1.22303232613902	-0.08354015899745
C	2.31950989421048	2.99999590010212	0.15416086013070
H	1.24092988804758	3.11143600779676	0.24863081602724
H	2.80878890662169	3.41305486230329	1.03660222918106
H	2.66802220025562	3.53632838627542	-0.72894906777040

E[PWPB95-D3(BJ)/def2-QZVPP] = -1043.334866 (Hartree)

Zero-point correction = 0.219127

Thermal correction to Energy = 0.234041

Thermal correction to Enthalpy= 0.234985

Thermal correction to Gibbs Free Energy= 0.177542

***trans*-S5 (1) in gas**

C	-3.17099957338921	-0.27925656075925	0.00001790105966
C	-2.32249224229359	0.83196968709446	0.00002998178544
C	-0.95303124208706	0.66653547009878	0.00002453499996
C	-0.39600832581344	-0.61275971775015	0.00000596993428
C	-1.24354083097113	-1.72079156298018	-0.00000608314280
C	-2.61195580226961	-1.56046309011171	-0.00000009808576
H	-2.73111323260536	1.83369636733617	0.00004415847532
H	-0.29591500033676	1.52653517475050	0.00003382198452
H	-0.79715786347901	-2.70811149753960	-0.00001989835759
N	0.97460108658772	-0.89205456730812	-0.00000174015272
N	1.72472246892096	0.10874311742845	0.00000429866023
C	3.06708836415253	-0.18542213916454	-0.00000006320927
C	3.80723201545134	-1.36070829138684	-0.00000302319902
H	3.41330538010878	-2.36211685828643	-0.00000270524734
C	-5.24859206829932	1.57079787417926	-0.00001954816413
H	-4.85600669735896	2.05367542411132	-0.89561474166629
H	-6.33487217381229	1.66075054475022	0.00002232019523
H	-4.85593221709474	2.05372957598442	0.89551393670786
S	-4.91442056563206	-0.18858041919005	0.00002001014470
H	-3.26108072393462	-2.42887665721549	-0.00000894373190
N	3.96481839038288	0.83737624051025	-0.00000204681179
N	5.21432459471711	0.40475322405759	-0.00000442197439
C	5.12932749747969	-0.92722401018808	-0.00000455887814
H	6.03442177859578	-1.51539508566634	-0.00000613659033
C	3.68751666726979	2.24974970607403	-0.00000391113080
H	4.12106407229254	2.71000437962436	-0.88841400629443
H	2.60904634464733	2.38494753586329	0.00000563156278
H	4.12108089877072	2.71000913568370	0.88839536112672

E[PWPB95-D3(BJ)/def2-QZVPP] = -1043.355661 (Hartree)

Zero-point correction = 0.219164

Thermal correction to Energy = 0.234142

Thermal correction to Enthalpy= 0.235086

Thermal correction to Gibbs Free Energy= 0.177599

***trans*-S5 (2) in gas**

C	3.13103369157739	0.25286891274655	0.00000302874644
C	2.40385819485202	-0.94114158628448	0.00000139648862
C	1.02447244080554	-0.92141487685127	-0.00000265643755
C	0.33728845701089	0.29217457097796	-0.00000608098129
C	1.06267818920875	1.48310742480640	-0.00000647415965
C	2.44062936678307	1.46801025322380	-0.00000171612144
H	2.91451572549266	-1.89482242602963	0.00000408046909
H	0.45865107296694	-1.84420760477054	-0.00000335182407
H	0.51778353736572	2.41982259488386	-0.00000994109428
N	-1.05791657655225	0.41870362420469	-0.00000903300291
N	-1.68533319331063	-0.66470850100528	-0.00000619505929
C	-3.05631146942154	-0.58523146025695	-0.00000112727789
C	-3.90583338459020	-1.68179274088725	0.00000736929224
H	-3.59939831798328	-2.71442443709078	0.00001038390584
C	5.38338224869663	-1.37298461042104	-0.00000566411863
H	5.03976904487825	-1.89227877992719	-0.89531690329090
H	6.47323089893963	-1.35541888491640	0.00000372545901
H	5.03975379957490	-1.89230008858452	0.89528739712079
S	4.87477181868499	0.34380774258666	0.00001022677240
H	2.99486790075941	2.39983857595649	-0.00000144516764
N	-3.85625779260626	0.52321062491796	0.00000326568080
N	-5.14232873077141	0.19346809908665	0.00000575857607
C	-5.18601298582995	-1.13618388794933	0.00000371367775
H	-6.14175001072061	-1.63696457208220	0.00000503531885
C	-3.50101498783928	1.92146078181397	0.00000380174614
H	-4.43801038052118	2.47388137281962	0.00000973269964
H	-2.91166980965888	2.16861841103445	0.88216554312973
H	-2.91167974779130	2.16862146799780	-0.88216387054788

E[PWPB95-D3(BJ)/def2-QZVPP] = -1043.353471 (Hartree)

Zero-point correction = 0.219319

Thermal correction to Energy = 0.234073

Thermal correction to Enthalpy= 0.235017

Thermal correction to Gibbs Free Energy= 0.178774

***cis*-S5 (1) in gas**

C	2.61149913603972	-0.26212483086171	-0.09511082550345
C	1.74954012105765	0.13460313408519	0.92596403919966
C	0.48151703068344	-0.40949113331194	1.02396547777338
C	0.05594204035689	-1.36950613834897	0.11439410807744
C	0.93122266830486	-1.81048756542682	-0.87299230109993
C	2.18377517850842	-1.24356837371793	-0.99317332908263
H	2.05803328106755	0.87302400683293	1.65377733409355
H	-0.18441152674813	-0.08687536216411	1.81551210463413
H	0.61123595327542	-2.58837848200916	-1.55611342810022
N	-1.19484682990358	-2.01680890349421	0.25695846352473
N	-2.26772700059029	-1.39220202133502	0.22085987342031
C	-2.38364425852308	-0.05426797280473	-0.13791895091719
C	-1.64859424181422	0.88372699859324	-0.86020686453232
H	-0.64824177437740	0.78278424727680	-1.24123238774627
C	4.39061955700872	1.57981738802109	0.97925308588218
H	4.33381737349736	1.11615560240709	1.96500782898637
H	5.38200173038722	2.01504198539496	0.85318393727941
H	3.64555612569338	2.37162729700198	0.89066724464891
S	4.22573224538057	0.37146583885358	-0.33113832882298
H	2.84934329505830	-1.57293420631966	-1.78345006084642
N	-3.59724958401137	0.52193077145742	0.09953235200830
N	-3.67836130593943	1.74640559941625	-0.38457268673988
C	-2.50854125934840	1.97046746249919	-0.98446847055278
H	-2.33546563344350	2.91258977418365	-1.48187032717046
C	-4.69751030736370	-0.04091623462216	0.84028186498504
H	-5.62492315918814	0.25120848887563	0.35167346768482
H	-4.58901173969789	-1.12299747061315	0.84802746758306
H	-4.69875411537035	0.33166810013056	1.86662631133325

E[PWPB95-D3(BJ)/def2-QZVPP] = -1043.336704 (Hartree)

Zero-point correction = 0.219134

Thermal correction to Energy = 0.233946

Thermal correction to Enthalpy= 0.234890

Thermal correction to Gibbs Free Energy= 0.177765

***cis*-S5 (2) in gas**

C	2.35498548027006	-0.25872814409152	0.03671349573909
C	1.37790147099590	0.45579511145625	0.73061799039330
C	0.11485788062239	-0.07477232052049	0.91677379702965
C	-0.19262868617770	-1.34442547026986	0.43095410071147
C	0.80470192749179	-2.08532426877591	-0.20406621438690
C	2.05127558981376	-1.54362199116030	-0.42519361879221
H	1.59232118338364	1.43792968111452	1.12951398433186
H	-0.62629345893918	0.50052177939116	1.45629856674235
H	0.57067500804992	-3.09205228408939	-0.52974988991466
N	-1.41325638177788	-2.02289044945051	0.64499337707819
N	-2.52266241111105	-1.46320711660196	0.67868820118848
C	-2.71843539350893	-0.11990554889034	0.32673717607656
C	-3.44303029709137	0.82737311744076	1.02354471256085
H	-3.90758682007910	0.69724578910091	1.98646800920264
C	3.97314658042709	1.96055435130788	0.45397931814528
H	3.81295615561958	1.91023490185995	1.53155990862197
H	4.96883591736958	2.36187023559964	0.26555624446087
H	3.23560945801113	2.61604203913973	-0.01025253615552
S	3.96558976446122	0.33157789614796	-0.28943426009454
H	2.80864753349814	-2.12068361178643	-0.94369605369247
N	-2.34052211180760	0.45575433627318	-0.84395246157568
N	-2.75959891155059	1.72068500533440	-0.93102496499362
C	-3.40392914696516	1.95957608789493	0.20205963044813
H	-3.83562684185761	2.93374518743333	0.37554356506807
C	-1.65851091164178	-0.14133736403589	-1.96146637048529
H	-2.05131260787936	0.30928923273751	-2.87047259751769
H	-0.58197904043229	0.03169267059307	-1.90130896845199
H	-1.84546692919462	-1.21408185315258	-1.97447414173819

E[PWPB95-D3(BJ)/def2-QZVPP] = -1043.332986 (Hartree)
Zero-point correction = 0.219256
Thermal correction to Energy = 0.233860
Thermal correction to Enthalpy= 0.234804
Thermal correction to Gibbs Free Energy= 0.178566

12. References

1. Trivella, A.; Coussan, S.; Chiavassa, T., Malonaldehyde Synthesis. *Synthetic Communications* **2008,** *38* (19), 3285-3290.
2. Zhang, Z.-Y.; He, Y.; Zhou, Y.; Yu, C.; Han, L.; Li, T., Pyrazolylazophenyl Ether-Based Photoswitches: Facile Synthesis, (Near-)Quantitative Photoconversion, Long Thermal Half-Life, Easy Functionalization, and Versatile Applications in Light-Responsive Systems. *Chemistry – A European Journal* **2019,** *25* (58), 13402-13410.
3. He, Y.; Shangguan, Z.; Zhang, Z.-Y.; Xie, M.; Yu, C.; Li, T., Azobispyrazole Family as Photoswitches Combining (Near-) Quantitative Bidirectional Isomerization and Widely Tunable Thermal Half-Lives from Hours to Years**. *Angewandte Chemie International Edition* **2021,** *60* (30), 16539-16546.
4. Neese, F., The ORCA program system. *WIREs Computational Molecular Science* **2012,** *2* (1), 73-78.
5. Lehtola, S.; Steigemann, C.; Oliveira, M. J. T.; Marques, M. A. L., Recent developments in libxc — A comprehensive library of functionals for density functional theory. *SoftwareX* **2018,** *7*, 1-5.
6. Grimme, S.; Ehrlich, S.; Goerigk, L., Effect of the damping function in dispersion corrected density functional theory. *Journal of Computational Chemistry* **2011,** *32* (7), 1456-1465.
7. Grimme, S.; Antony, J.; Ehrlich, S.; Krieg, H., A consistent and accurate ab initio parametrization of density functional dispersion correction (DFT-D) for the 94 elements H-Pu. *The Journal of Chemical Physics* **2010,** *132* (15), 154104.
8. Weigend, F.; Ahlrichs, R., Balanced basis sets of split valence, triple zeta valence and quadruple zeta valence quality for H to Rn: Design and assessment of accuracy. *Physical Chemistry Chemical Physics* **2005,** *7* (18), 3297-3305.
9. Weigend, F., Accurate Coulomb-fitting basis sets for H to Rn. *Physical Chemistry Chemical Physics* **2006,** *8* (9), 1057-1065.
10. Marenich, A. V.; Cramer, C. J.; Truhlar, D. G., Universal Solvation Model Based on Solute Electron Density and on a Continuum Model of the Solvent Defined by the Bulk Dielectric Constant and Atomic Surface Tensions. *The Journal of Physical Chemistry B* **2009,** *113* (18), 6378-6396.
11. Hehre, W. J.; Lathan, W. A., Self‐Consistent Molecular Orbital Methods. XIV. An Extended Gaussian‐Type Basis for Molecular Orbital Studies of Organic Molecules. Inclusion of Second Row Elements. *The Journal of Chemical Physics* **1972,** *56* (11), 5255-5257.
12. Francl, M. M.; Pietro, W. J.; Hehre, W. J.; Binkley, J. S.; Gordon, M. S.; DeFrees, D. J.; Pople, J. A., Self‐consistent molecular orbital methods. XXIII. A polarization‐type basis set for second‐row elements. *The Journal of Chemical Physics* **1982,** *77* (7), 3654-3665.
13. Zhao, Y.; Schultz, N. E.; Truhlar, D. G., Design of Density Functionals by Combining the Method of Constraint Satisfaction with Parametrization for Thermochemistry, Thermochemical Kinetics, and Noncovalent Interactions. *Journal of Chemical Theory and Computation* **2006,** *2* (2), 364-382.
14. Furche, F.; Ahlrichs, R., Adiabatic time-dependent density functional methods for excited state properties. *The Journal of Chemical Physics* **2002,** *117* (16), 7433-7447.
15. Lu, T.; Chen, F., Multiwfn: A multifunctional wavefunction analyzer. *Journal of Computational Chemistry* **2012,** *33* (5), 580-592.
16. Humphrey, W.; Dalke, A.; Schulten, K., VMD: Visual molecular dynamics. *Journal of Molecular Graphics* **1996,** *14* (1), 33-38.
17. Kuhn, H. J.; Braslavsky, S. E.; Schmidt, R., Chemical actinometry (IUPAC Technical Report). *Pure*

and Applied Chemistry **2004,** *76* (12), 2105-2146.
18. Zhang, Z.-Y.; He, Y.; Wang, Z.; Xu, J.; Xie, M.; Tao, P.; Ji, D.; Moth-Poulsen, K.; Li, T., Photochemical Phase Transitions Enable Coharvesting of Photon Energy and Ambient Heat for Energetic Molecular Solar Thermal Batteries That Upgrade Thermal Energy. *Journal of the American Chemical Society* **2020,** *142* (28), 12256-12264.
19. Li, Y.; Zhang, Z.; Wang, M,; Men, X.; Xue, Q., Environmentally safe, substrate-independent and repairable nanoporous coatings: large-scale preparation, high transparency and antifouling properties. *Journal of Materials Chemistry A* **2017,** *5* (38), 20277-20288.
20. Sheldrick, G., A short history of SHELX. *Acta Crystallographica Section A* **2008,** *64* (1), 112-122.
21. Sheldrick, G., SHELXT - Integrated space-group and crystal-structure determination. *Acta Crystallographica Section A* **2015,** *71* (1), 3-8.
22. Sheldrick, G., Crystal structure refinement with SHELXL. *Acta Crystallographica Section C* **2015,** *71* (1), 3-8.
23. Hubschle, C. B.; Sheldrick, G. M.; Dittrich, B., ShelXle: a Qt graphical user interface for SHELXL. *Journal of Applied Crystallography* **2011,** *44* (6), 1281-1284.
24. Dolomanov, O. V.; Bourhis, L. J.; Gildea, R. J.; Howard, J. A. K.; Puschmann, H., OLEX2: a complete structure solution, refinement and analysis program. *Journal of Applied Crystallography* **2009,** *42* (2), 339-341.
